# Supplementary material for: Sulfaguanidine Hybrid with Some New Pyridine-2-One Derivatives: Design, Synthesis, and Antimicrobial Activity against Multidrug-Resistant Bacteria as Dual DNA Gyrase and DHFR Inhibitors
Source: Antibiotics (Basel). 2021 Feb 5;10(2):162. doi: 10.3390/antibiotics10020162 (PMC7915026; doi:10.3390/antibiotics10020162)
Supplement: Supplementary file 1 [file antibiotics-10-00162-s001.pdf]

antibiotics

MDPI

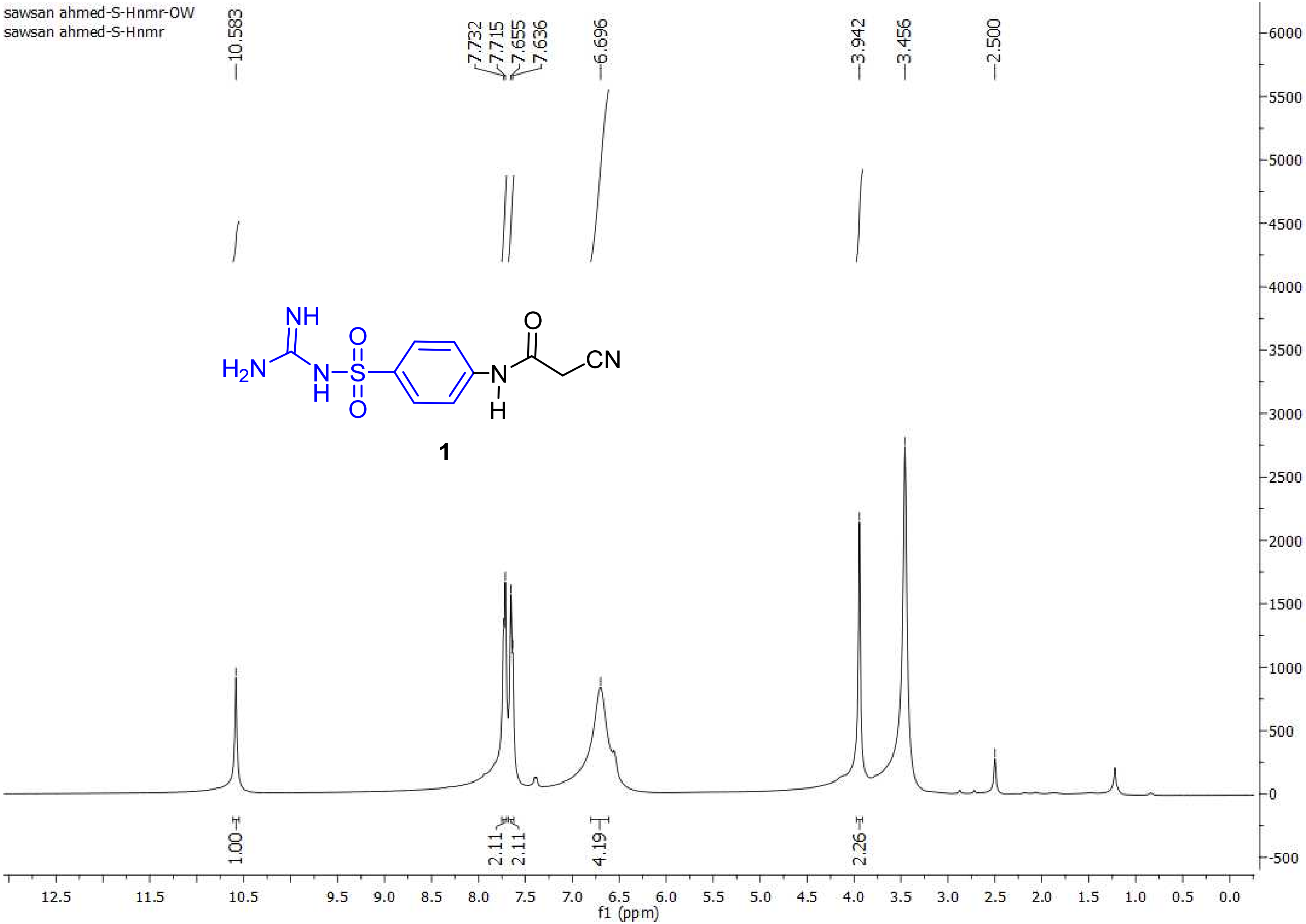

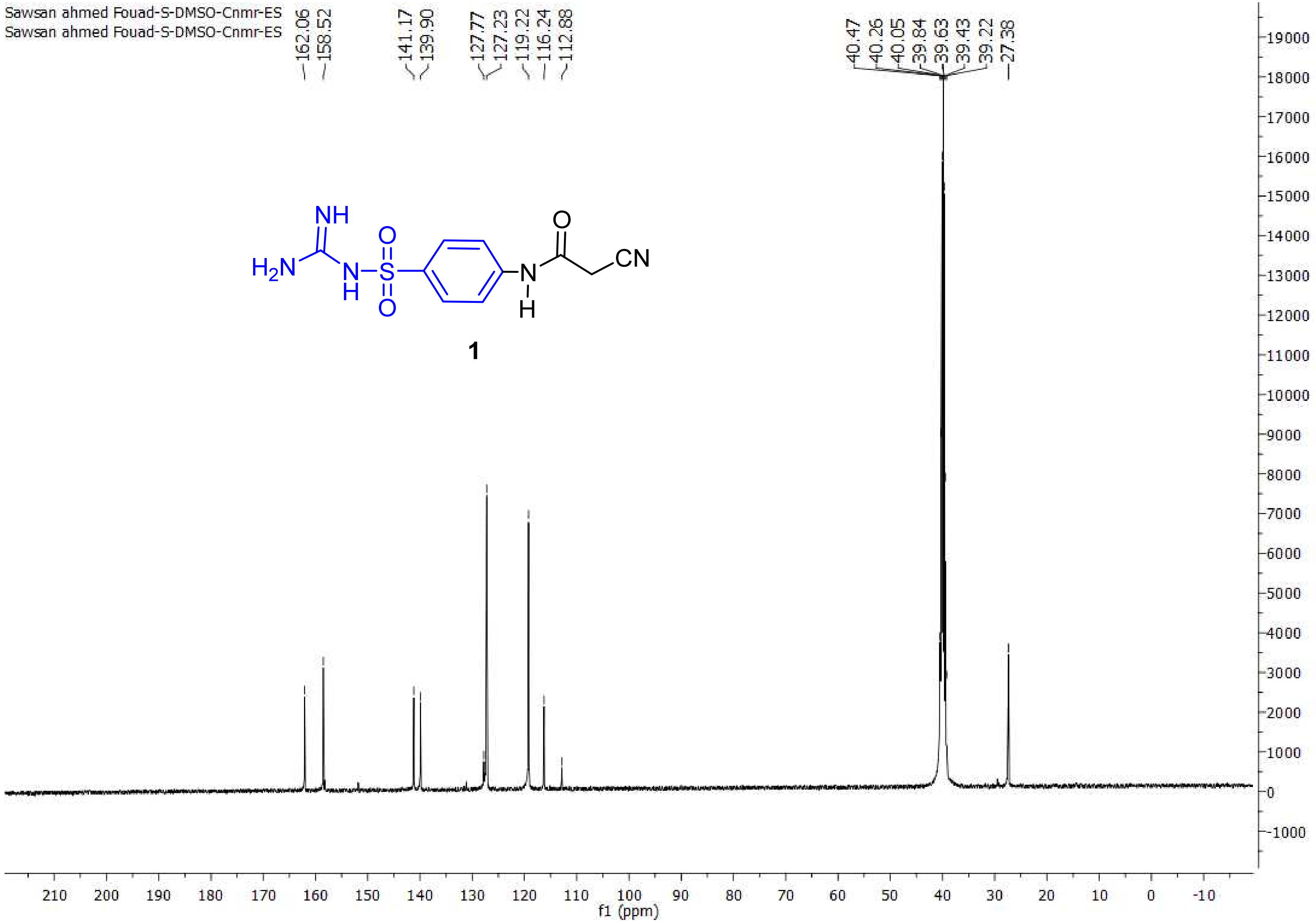
Sawsan ahmed Fouad-S-DMSO-Cnmr-ES
Sawsan ahmed Fouad-S-DMSO-Cnmr-ES
162.06
158.52
141.17
139.90
127.77
127.23
119.22
116.24
112.88
40.47
40.26
40.05
39.84
39.63
39.43
39.22
27.38
NH
H2N
N
H
O
S
O
N
H
O
CN
1
f1 (ppm)

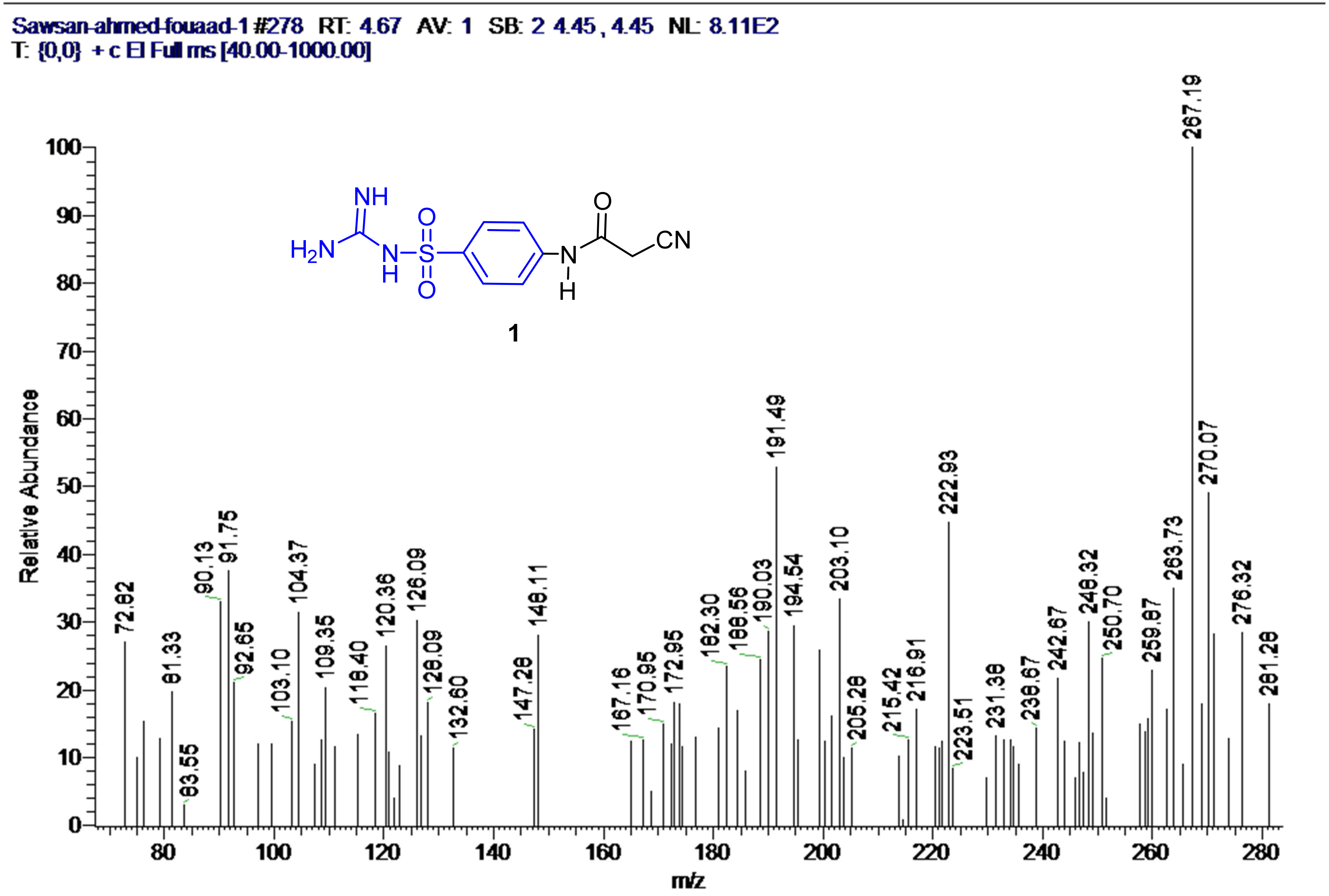
Sawsan-ahmed-fouaad-1 #278 RT: 4.67 AV: 1 SB: 2 4.45 , 4.45 NL: 8.11E2
T: {0,0} + c EI Full ms [40.00-1000.00]
NH
H2N
N
H
O
S
O
N
H
O
CN
1
Relative Abundance
m/z
267.19
270.07
191.49
222.93
263.73
246.32
203.10
194.54
91.75
90.13
276.32
250.70
104.37
126.09
148.11
72.82
120.36
182.30
186.56
190.03
242.67
259.67
172.95
109.35
81.33
92.65
116.40
128.09
216.91
281.28
231.38
238.67
215.42
103.10
170.95
167.16
147.28
132.60
205.28
223.51
83.55

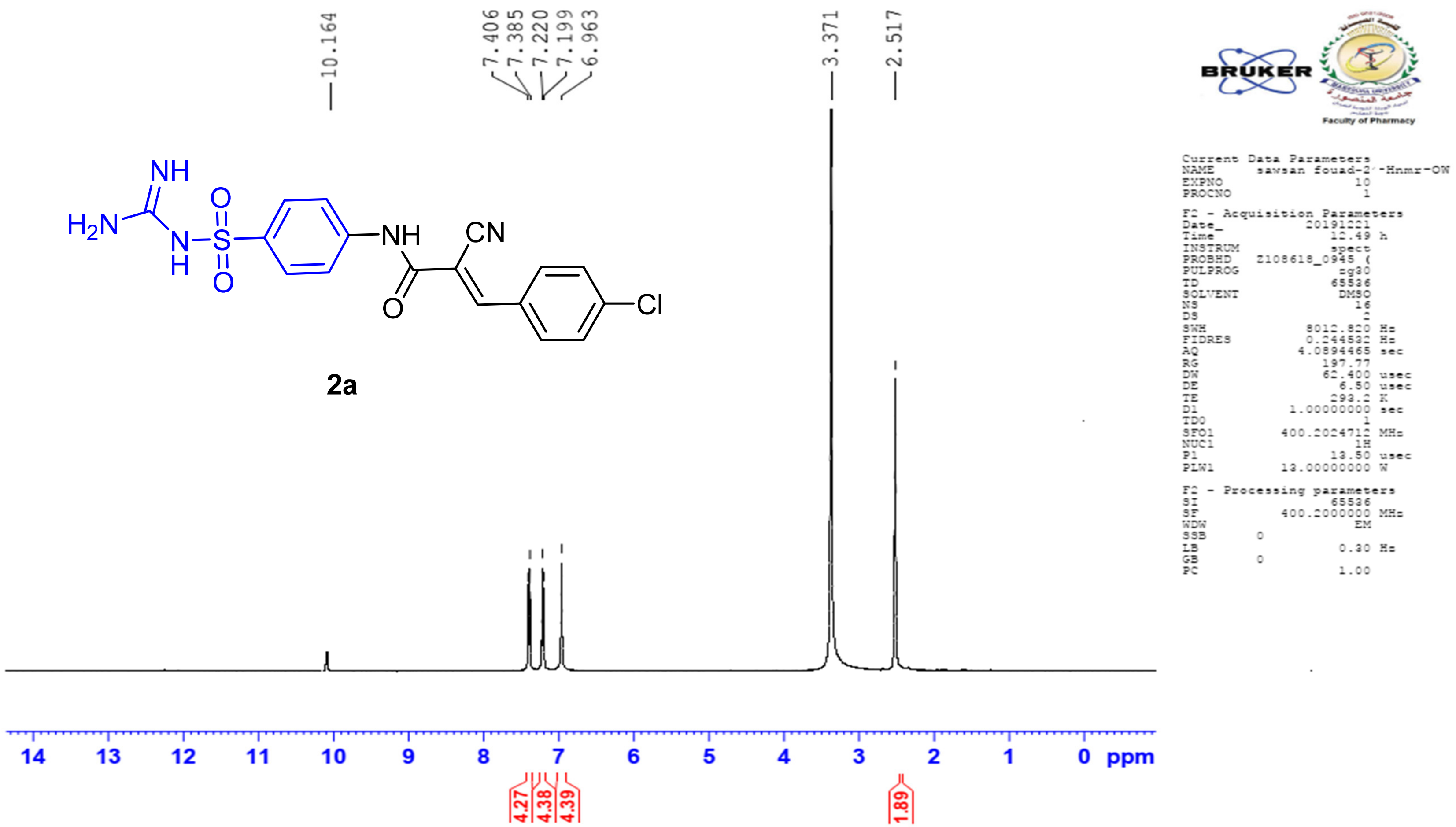
10.164
7.406
7.385
7.220
7.199
6.963
3.371
2.517
NH
$H_2N$
N
H
S
O
O
NH
CN
O
Cl
2a
BRUKER
Faculty of Pharmacy
Current Data Parameters
NAME sawsan fouad-2-Hnmr-OW
EXPNO 10
PROCNO 1
F2 - Acquisition Parameters
Date_ 20191221
Time 12.49 h
INSTRUM spect
PROBHD Z108618_0945 (
PULPROG zg30
TD 65536
SOLVENT DMSO
NS 16
DS 2
SWH 8012.820 Hz
FIDRES 0.244532 Hz
AQ 4.0894465 sec
RG 197.77
DW 62.400 usec
DE 6.50 usec
TE 293.2 K
D1 1.00000000 sec
TD0 1
SFO1 400.2024712 MHz
NUC1 1H
P1 13.50 usec
PLW1 13.00000000 W
F2 - Processing parameters
SI 65536
SF 400.2000000 MHz
WDW EM
SSB 0
LB 0.30 Hz
GB 0
PC 1.00
14
13
12
11
10
9
8
7
6
5
4
3
2
1
0
ppm
4.27
4.38
4.39
1.89

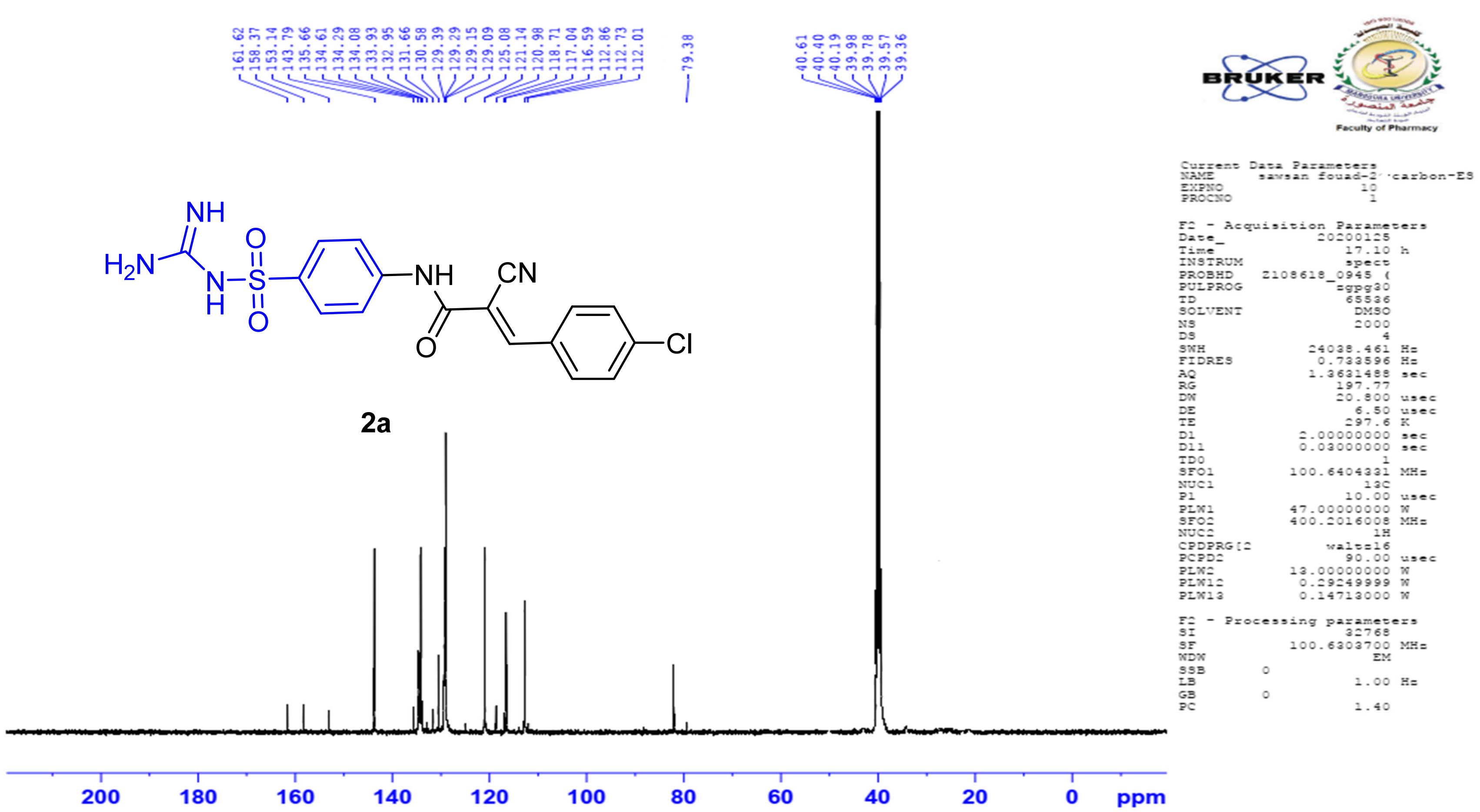
161.62
158.37
153.14
143.79
135.66
134.61
134.29
134.08
133.93
132.95
131.66
130.58
129.39
129.29
129.15
129.09
125.08
121.14
120.98
118.71
117.04
116.59
112.86
112.73
112.01
79.38
40.61
40.40
40.19
39.98
39.78
39.57
39.36
BRUKER
Faculty of Pharmacy
NH
H2N
N
H
O
S
O
NH
CN
O
Cl
2a
Current Data Parameters
EXPNO 10
PROCNO 1
F2 - Acquisition Parameters
Date_ 20200125
Time 17.10 h
INSTRUM spect
PULPROG zgpg30
TD 65536
SOLVENT DMSO
NS 2000
DS 4
SWH 24038.461 Hz
FIDRES 0.733596 Hz
AQ 1.3631488 sec
RG 197.77
DW 20.800 usec
DE 6.50 usec
TE 297.6 K
D1 2.00000000 sec
D11 0.03000000 sec
TD0 1
SFO1 100.6404331 MHz
NUC1 13C
P1 10.00 usec
PLW1 47.00000000 W
SFO2 400.2016008 MHz
NUC2 1H
CPDPRG[2 waltz16
PCPD2 90.00 usec
PLW2 13.00000000 W
PLW12 0.29249999 W
PLW13 0.14713000 W
F2 - Processing parameters
SI 32768
SF 100.6303700 MHz
WDW EM
SSB 0
LB 1.00 Hz
GB 0
PC 1.40
200
180
160
140
120
100
80
60
40
20
0
ppm

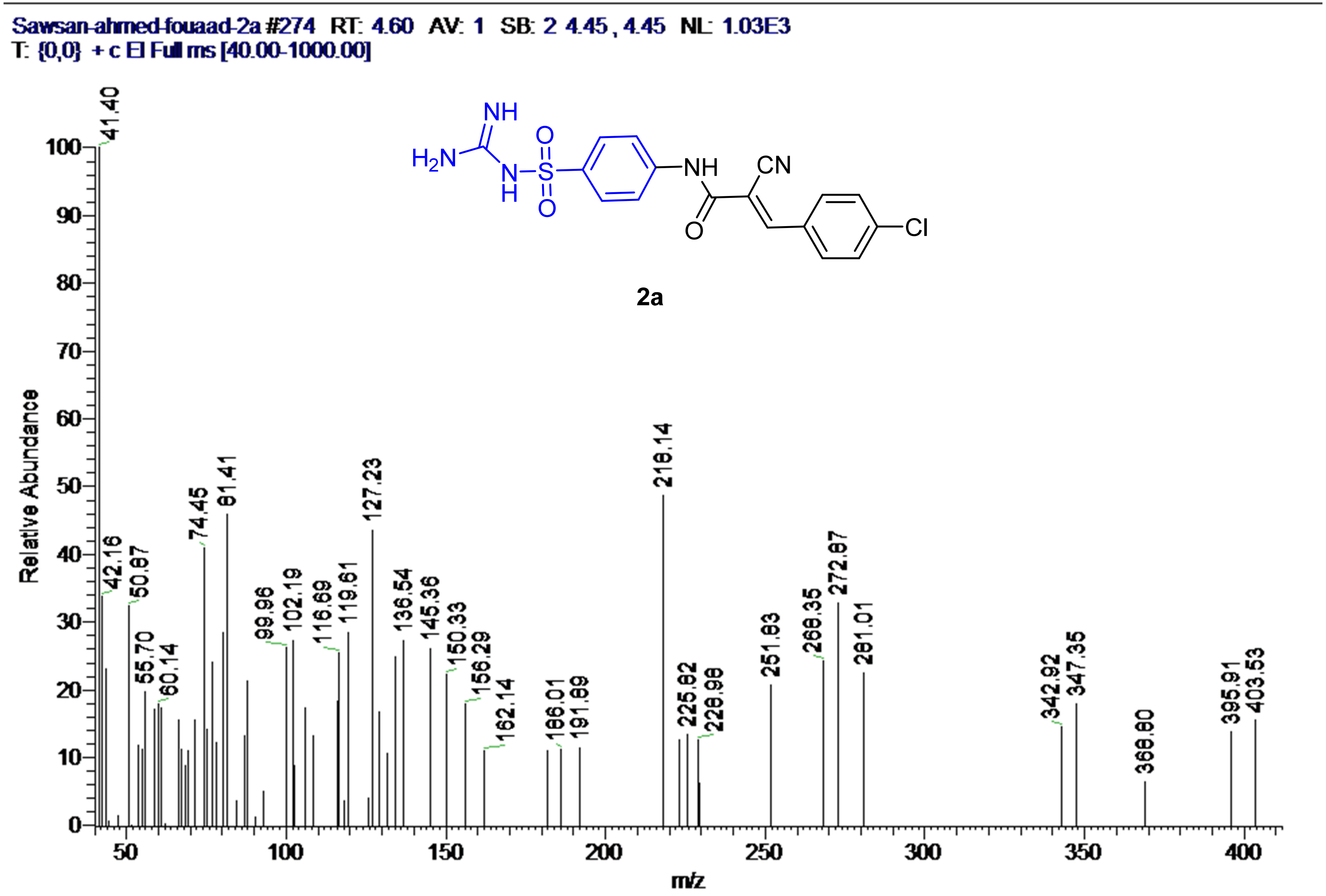
Sawsan-ahmed-fouaad-2a #274 RT: 4.60 AV: 1 SB: 2 4.45 , 4.45 NL: 1.03E3
T: {0,0} + c EI Full ms [40.00-1000.00]
Relative Abundance
m/z
2a
41.40
42.16
50.87
55.70
60.14
74.45
81.41
99.96
102.19
116.69
119.61
127.23
136.54
145.36
150.33
156.29
162.14
186.01
191.89
218.14
225.82
228.98
251.83
268.35
272.87
281.01
342.92
347.35
368.80
395.91
403.53

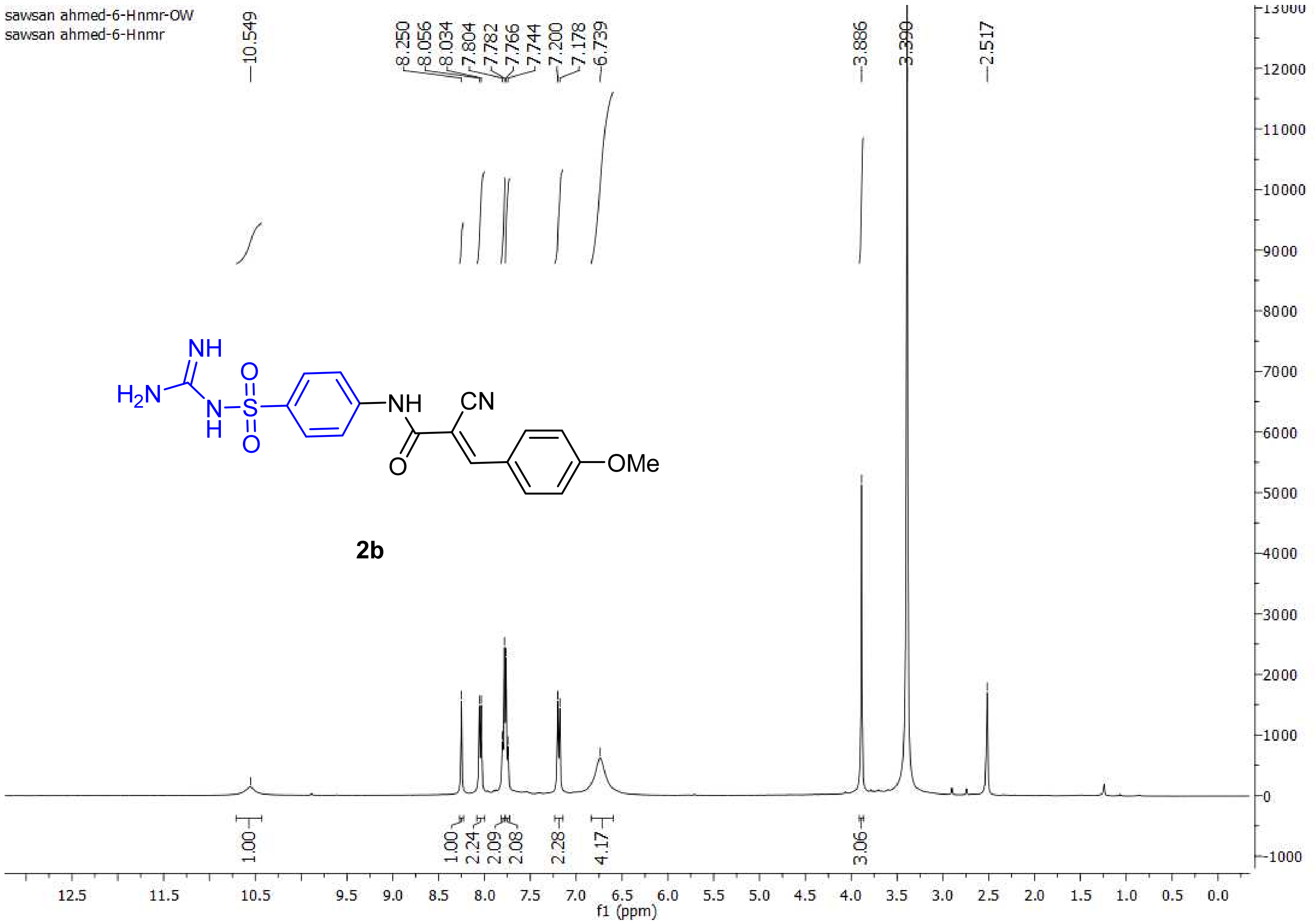
sawsan ahmed-6-Hnmr-OW
sawsan ahmed-6-Hnmr
10.549
8.250
8.056
8.034
7.804
7.782
7.766
7.744
7.200
7.178
6.739
3.886
3.390
2.517
2b
NH
H2N
N
H
S
O
O
NH
CN
O
OMe
1.00
1.00
2.24
2.09
2.08
2.28
4.17
3.06
f1 (ppm)

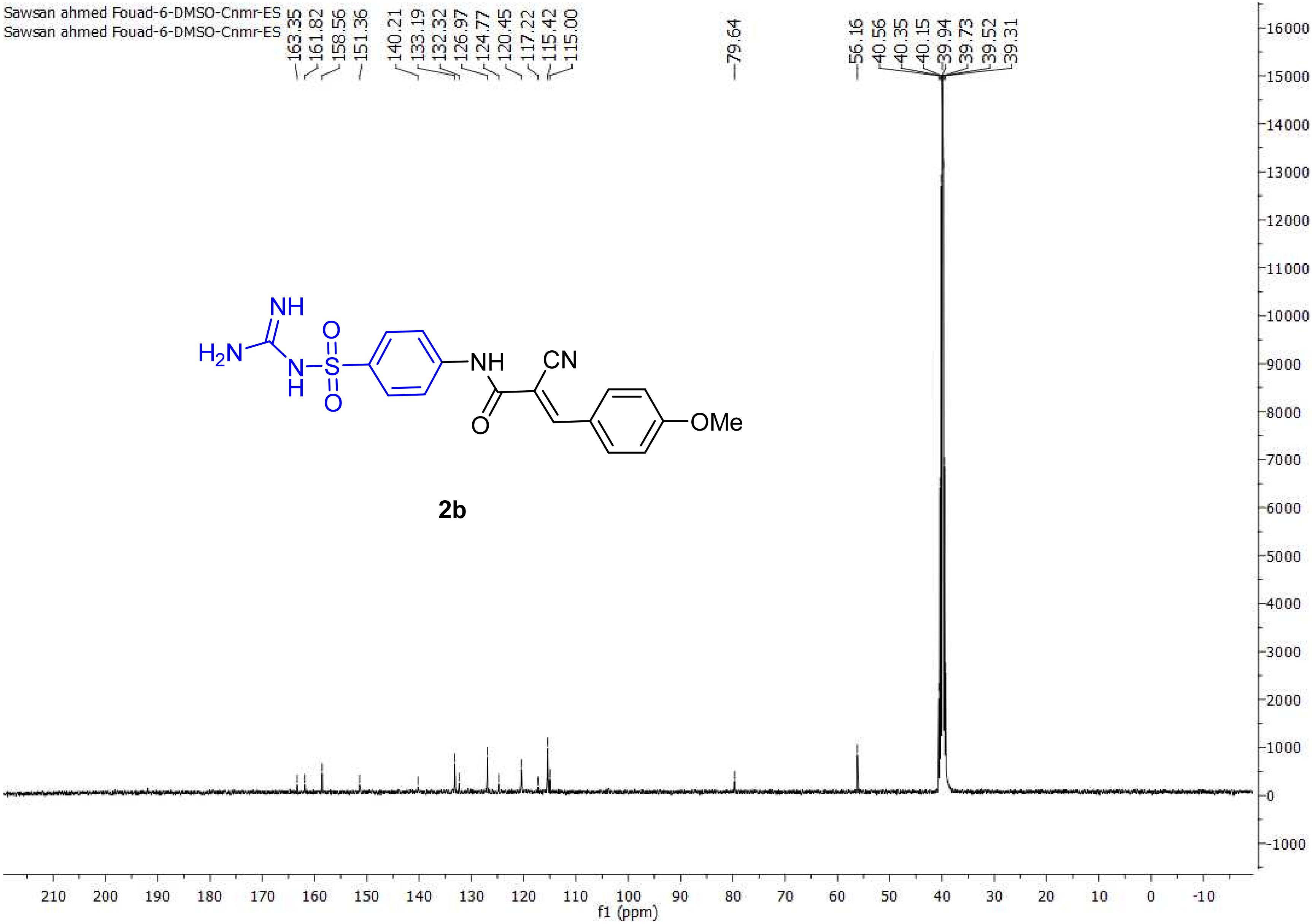
Sawsan ahmed Fouad-6-DMSO-Cnmr-ES
Sawsan ahmed Fouad-6-DMSO-Cnmr-ES
163.35
161.82
158.56
151.36
140.21
133.19
132.32
126.97
124.77
120.45
117.22
115.42
115.00
79.64
56.16
40.56
40.35
40.15
39.94
39.73
39.52
39.31
NH
$H_2N$
N
H
S
O
O
NH
CN
O
OMe
2b
f1 (ppm)

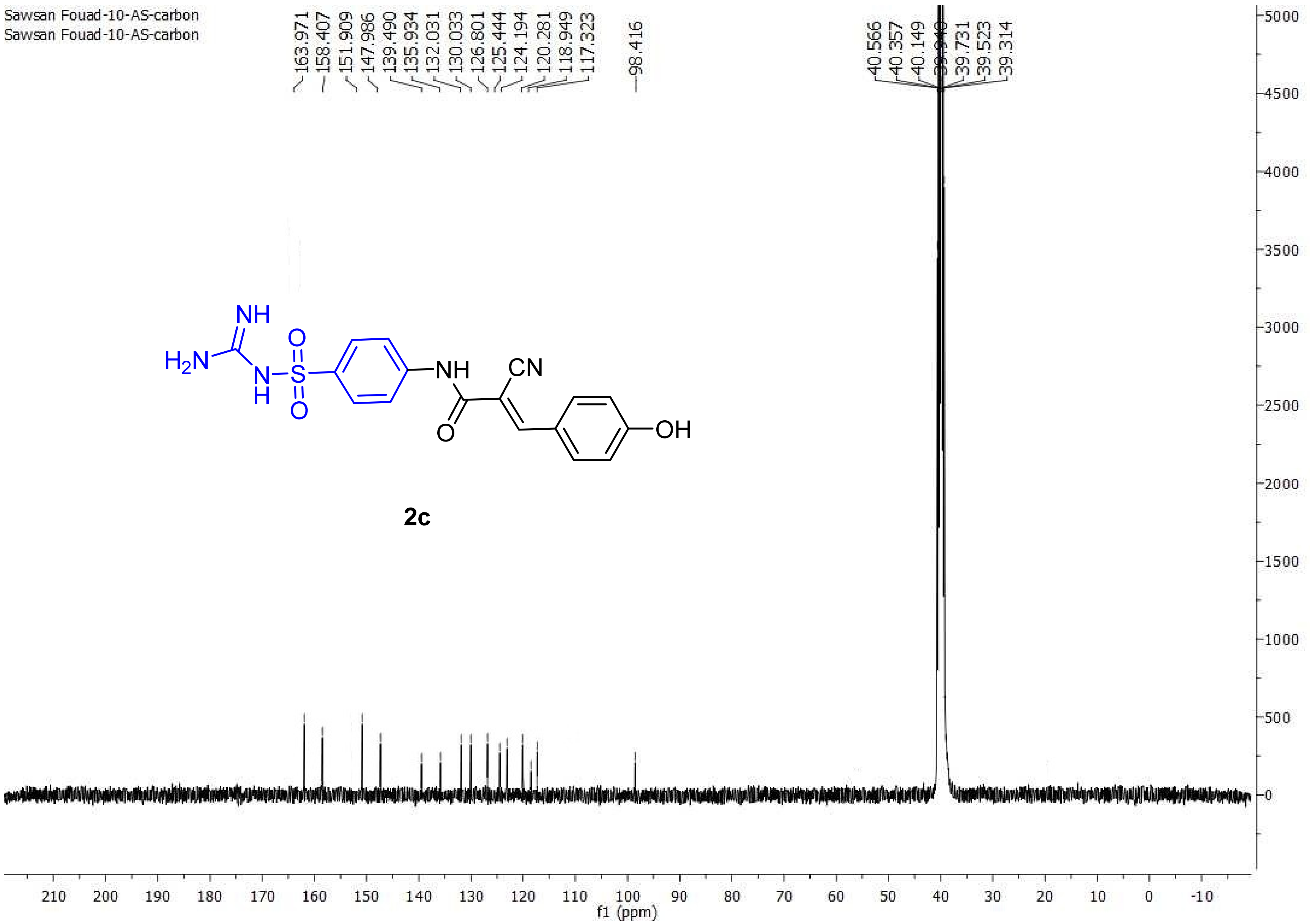
Sawsan Fouad-10-AS-carbon
Sawsan Fouad-10-AS-carbon
163.971
158.407
151.909
147.986
139.490
135.934
132.031
130.033
126.801
125.444
124.194
120.281
118.949
117.323
98.416
40.566
40.357
40.149
39.731
39.523
39.314
NH
$H_2N$
N
H
O
S
O
NH
CN
O
OH
2c
f1 (ppm)

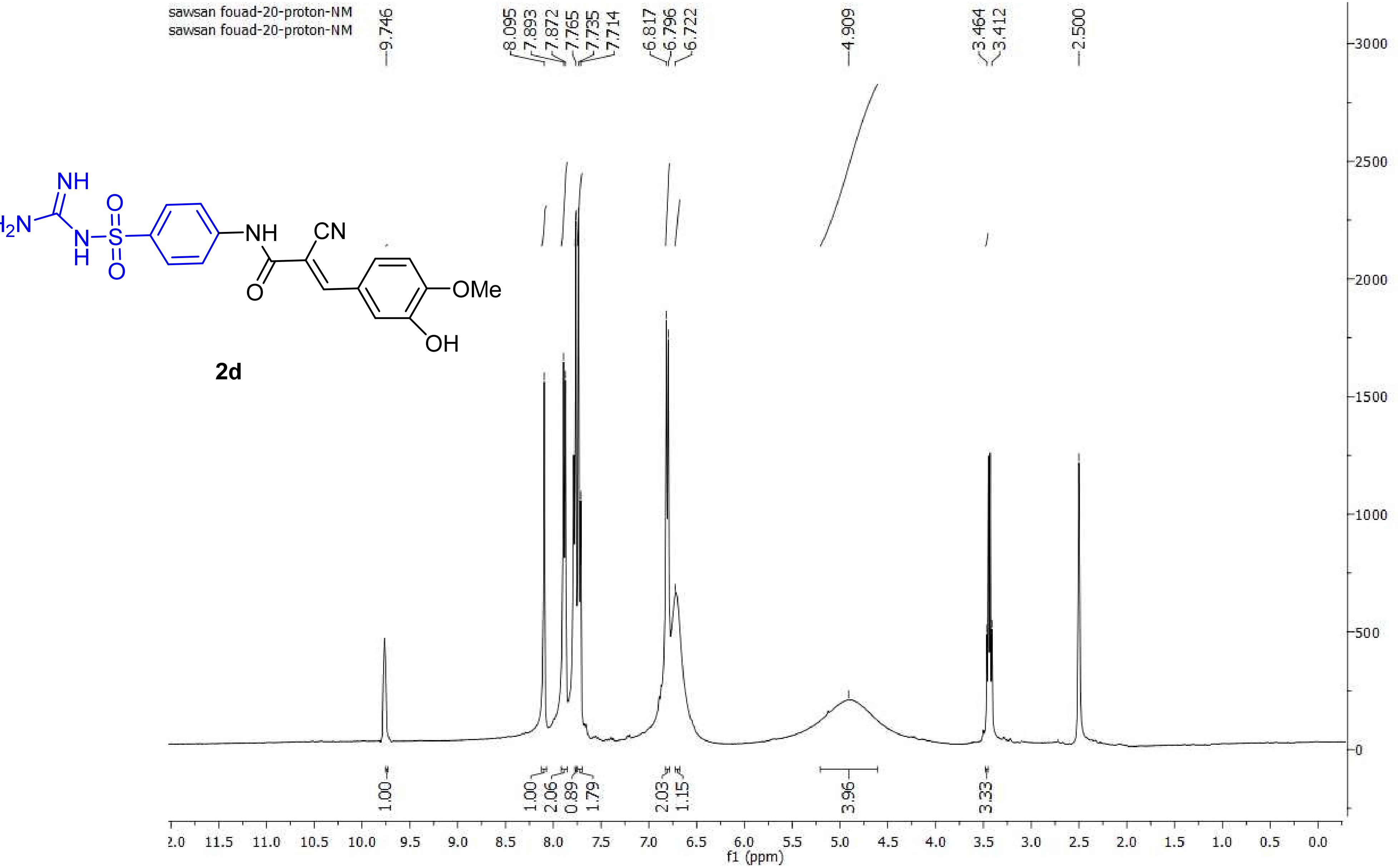
sawsan fouad-20-proton-NM
sawsan fouad-20-proton-NM
9.746
8.095
7.893
7.872
7.765
7.735
7.714
6.817
6.796
6.722
4.909
3.464
3.412
2.500
NH
H2N
N
H
S
O
O
NH
CN
O
OMe
OH
1.00
1.00
2.06
0.89
1.79
2.03
1.15
3.96
3.33
f1 (ppm)

**2d**

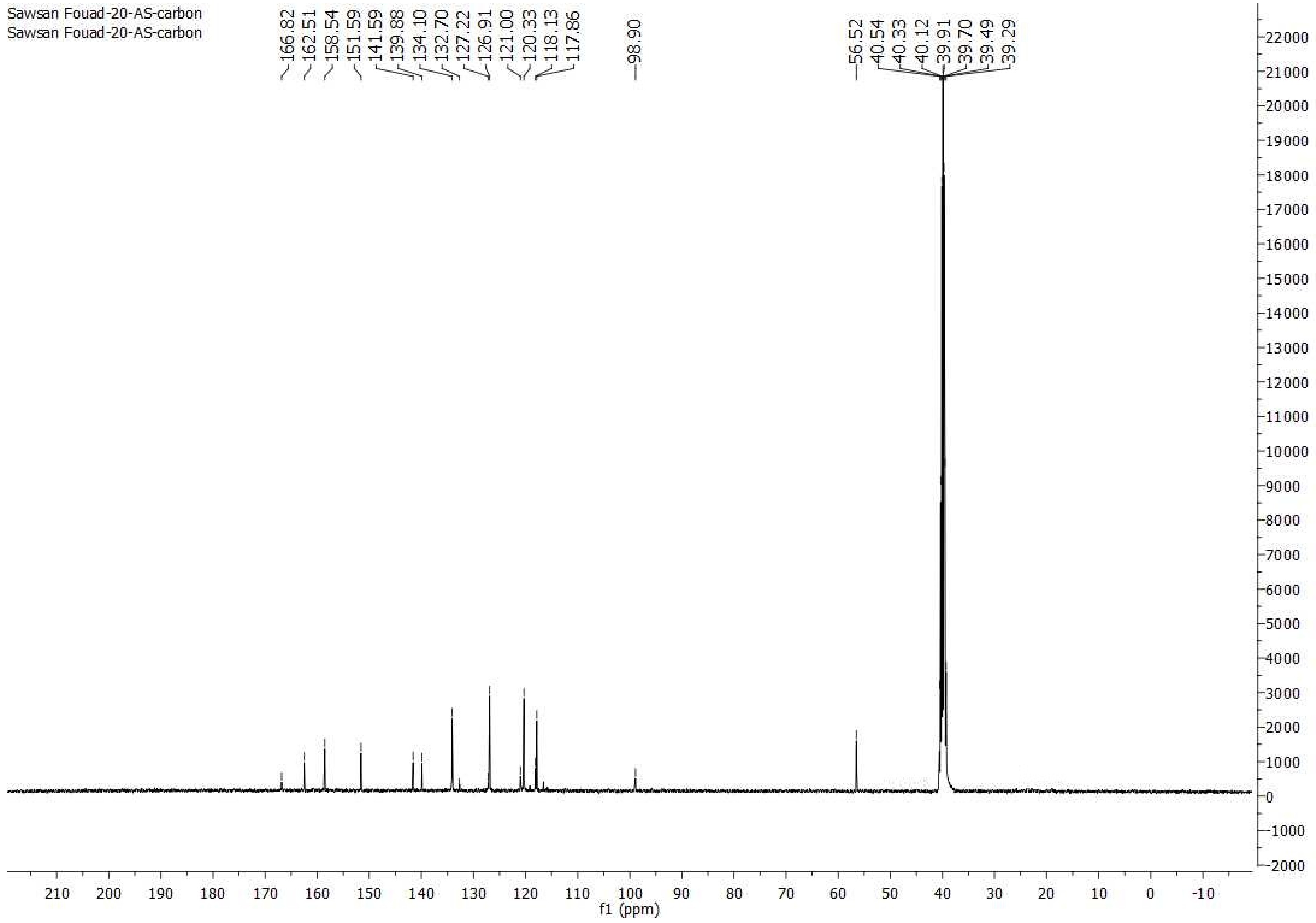
Sawsan Fouad-20-AS-carbon
Sawsan Fouad-20-AS-carbon
166.82
162.51
158.54
151.59
141.59
139.88
134.10
132.70
127.22
126.91
121.00
120.33
118.13
117.86
98.90
56.52
40.54
40.33
40.12
39.91
39.70
39.49
39.29
f1 (ppm)

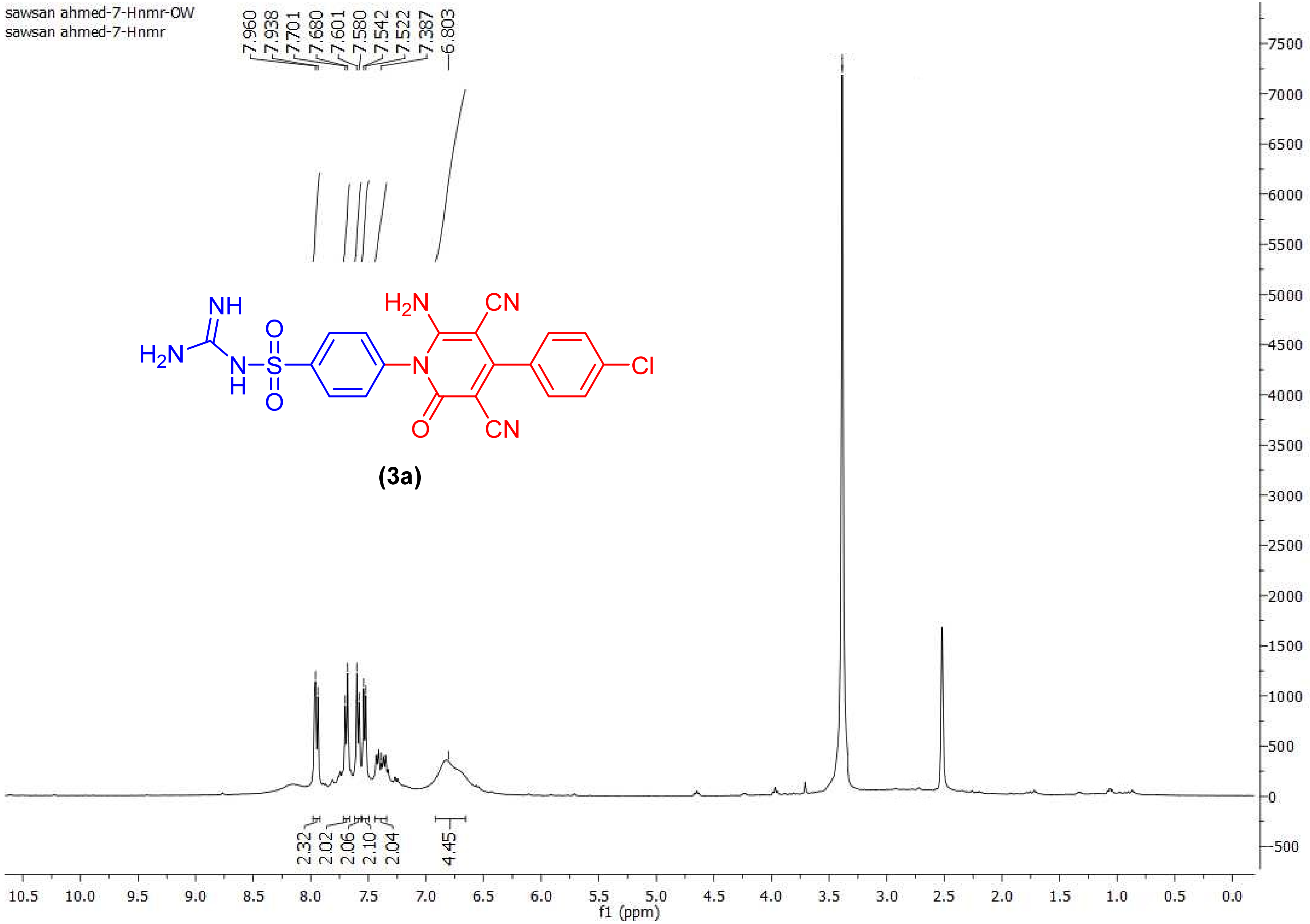
sawsan ahmed-7-Hnmr-OW
sawsan ahmed-7-Hnmr
7.960
7.938
7.701
7.680
7.601
7.580
7.542
7.522
7.387
6.803
NH
H2N
N-S
H
O
O
H2N
CN
N
Cl
O
CN
(3a)
2.32
2.02
2.06
2.10
2.04
4.45
f1 (ppm)

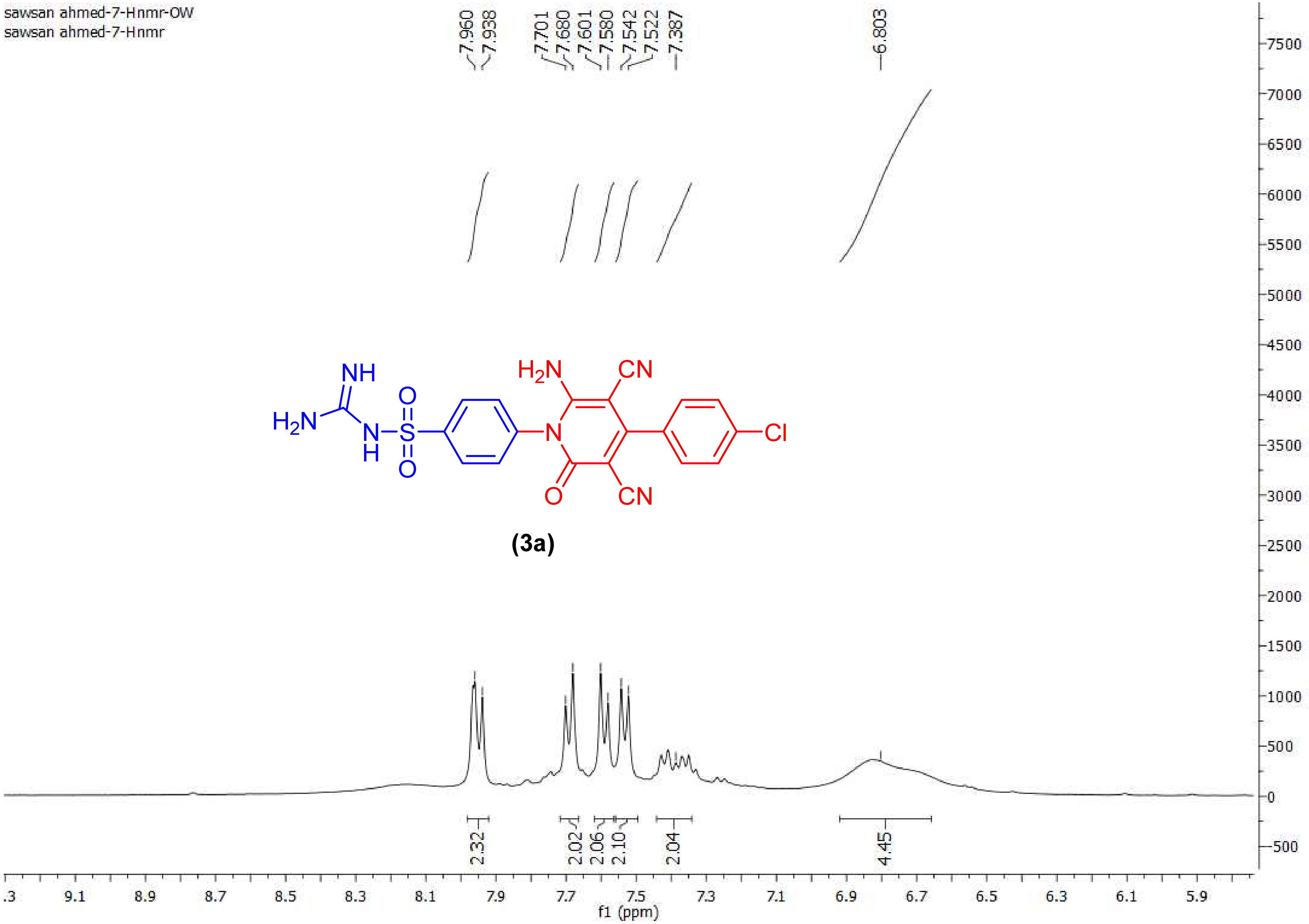
sawsan ahmed-7-Hnmr-OW
sawsan ahmed-7-Hnmr
7.960
7.938
7.701
7.680
7.601
7.580
7.542
7.522
7.387
6.803
NH
H2N
N
H
O
S
O
H2N
CN
N
Cl
O
CN
(3a)
2.32
2.02
2.06
2.10
2.04
4.45
f1 (ppm)

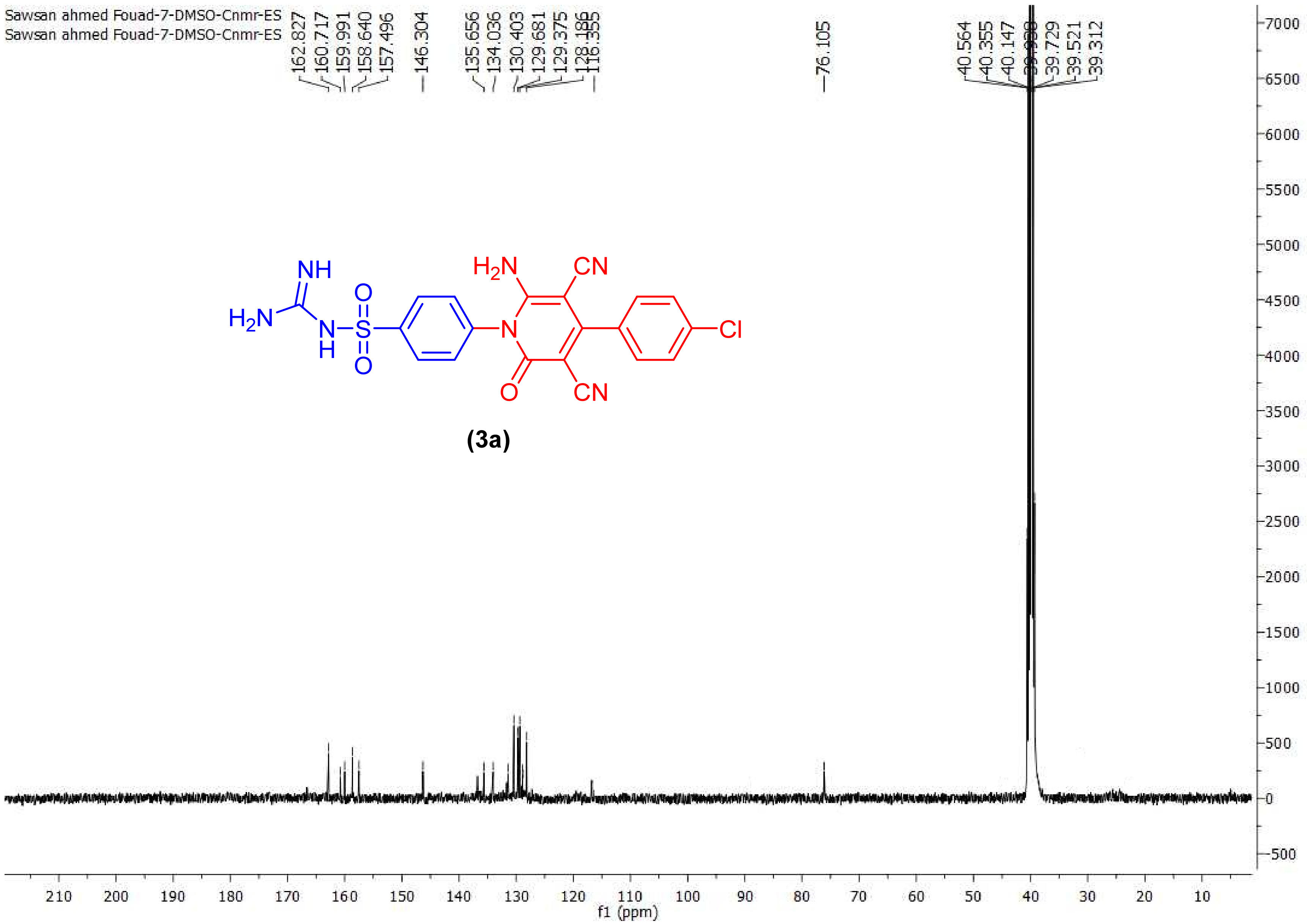
Sawsan ahmed Fouad-7-DMSO-Cnmr-ES
Sawsan ahmed Fouad-7-DMSO-Cnmr-ES
162.827
160.717
159.991
158.640
157.496
146.304
135.656
134.036
130.403
129.681
129.375
76.105
40.564
40.355
40.147
39.729
39.521
39.312
NH
$H_2N$
N
H
S
O
O
$H_2N$
CN
N
O
CN
Cl
(3a)
f1 (ppm)

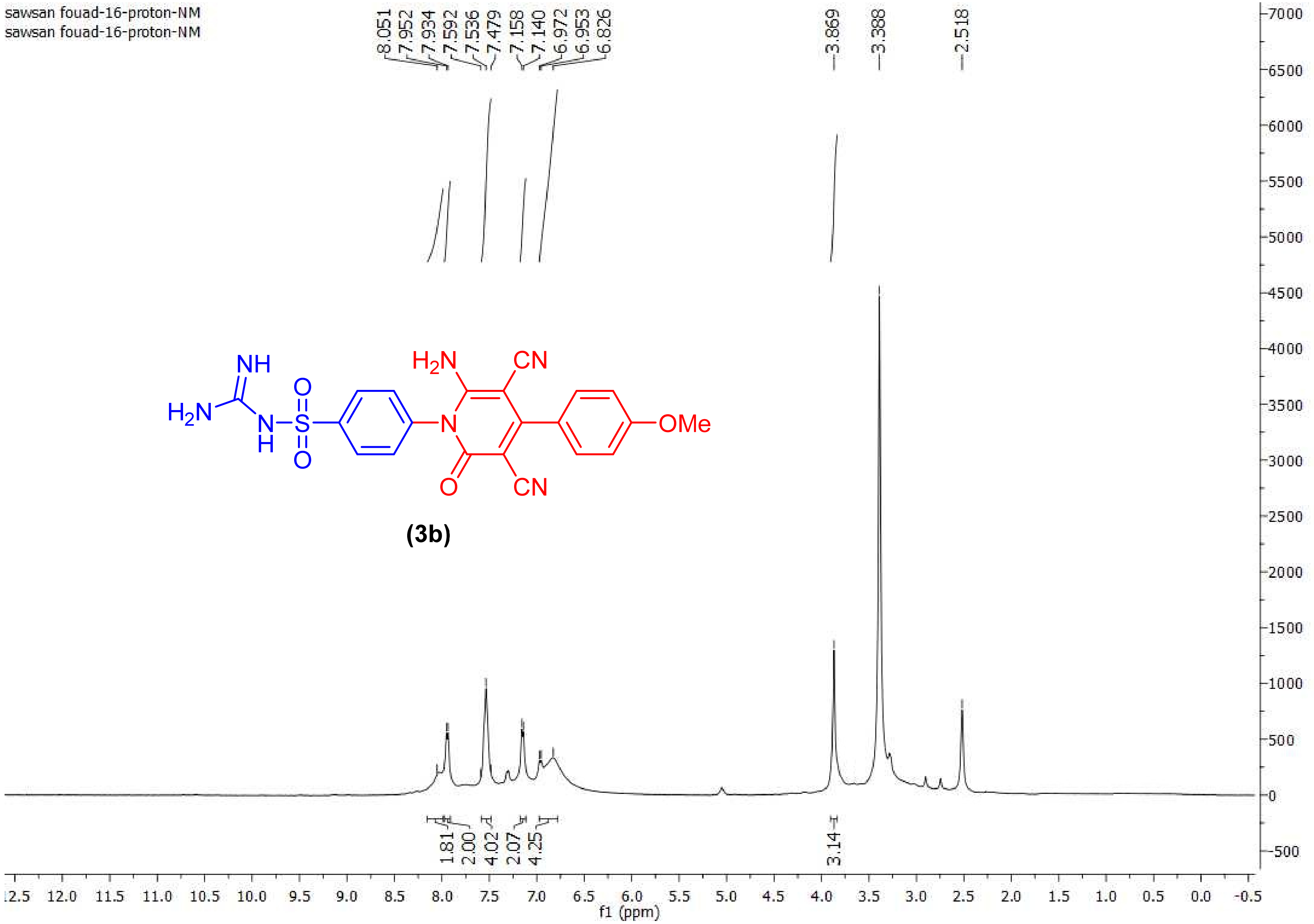

sawsan fouad-16-proton-NM
sawsan fouad-16-proton-NM
8.051
7.952
7.934
7.592
7.536
7.479
7.158
7.140
6.972
6.953
6.826
3.869
3.388
2.518
(3b)
1.81
2.00
4.02
2.07
4.25
3.14
f1 (ppm)

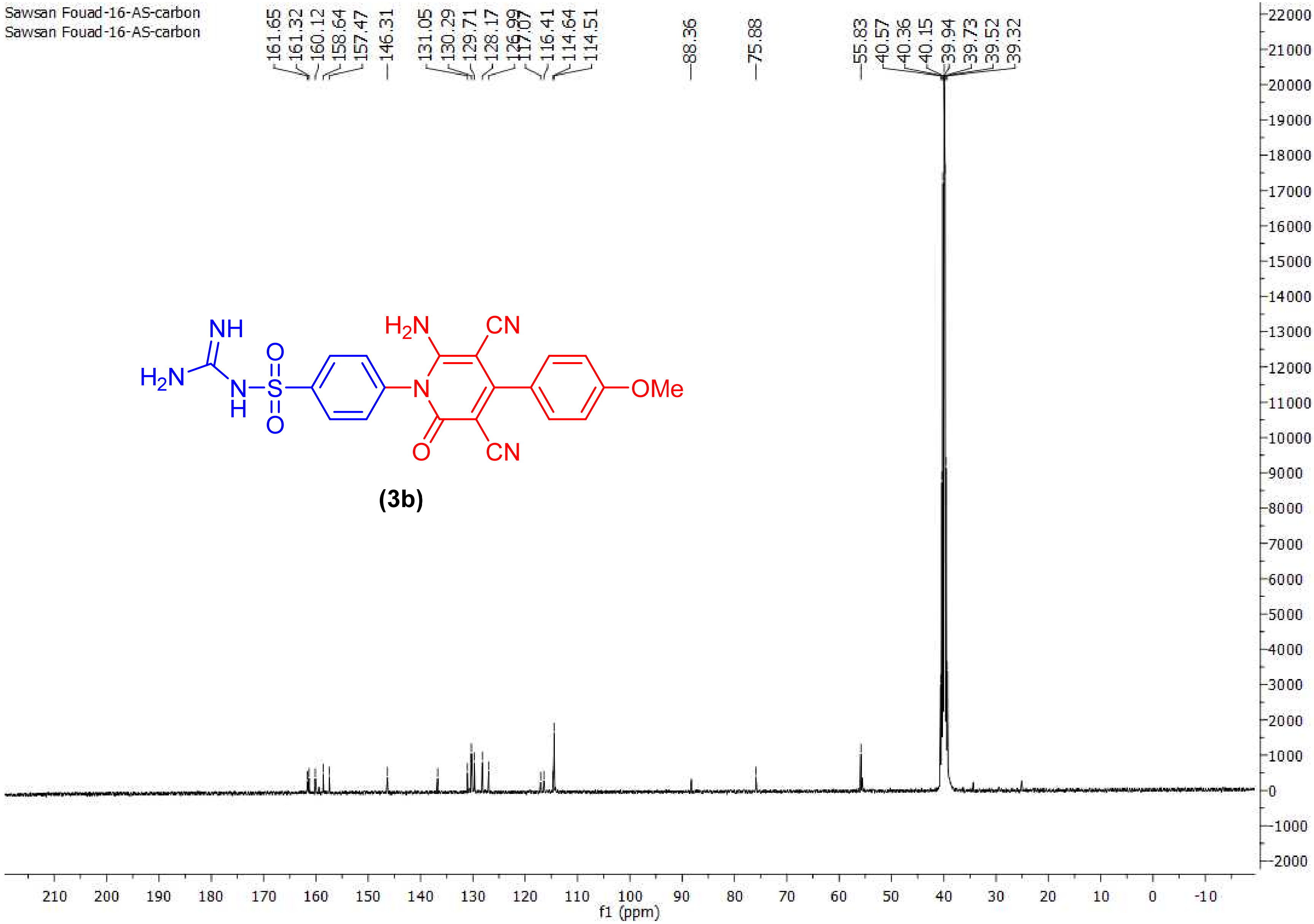
Sawsan Fouad-16-AS-carbon
Sawsan Fouad-16-AS-carbon
161.65
161.32
160.12
158.64
157.47
146.31
131.05
130.29
129.71
128.17
126.99
117.07
116.41
114.64
114.51
88.36
75.88
55.83
40.57
40.36
40.15
39.94
39.73
39.52
39.32
NH
H2N
N
H
O
S
O
H2N
CN
N
O
CN
OMe
(3b)
f1 (ppm)

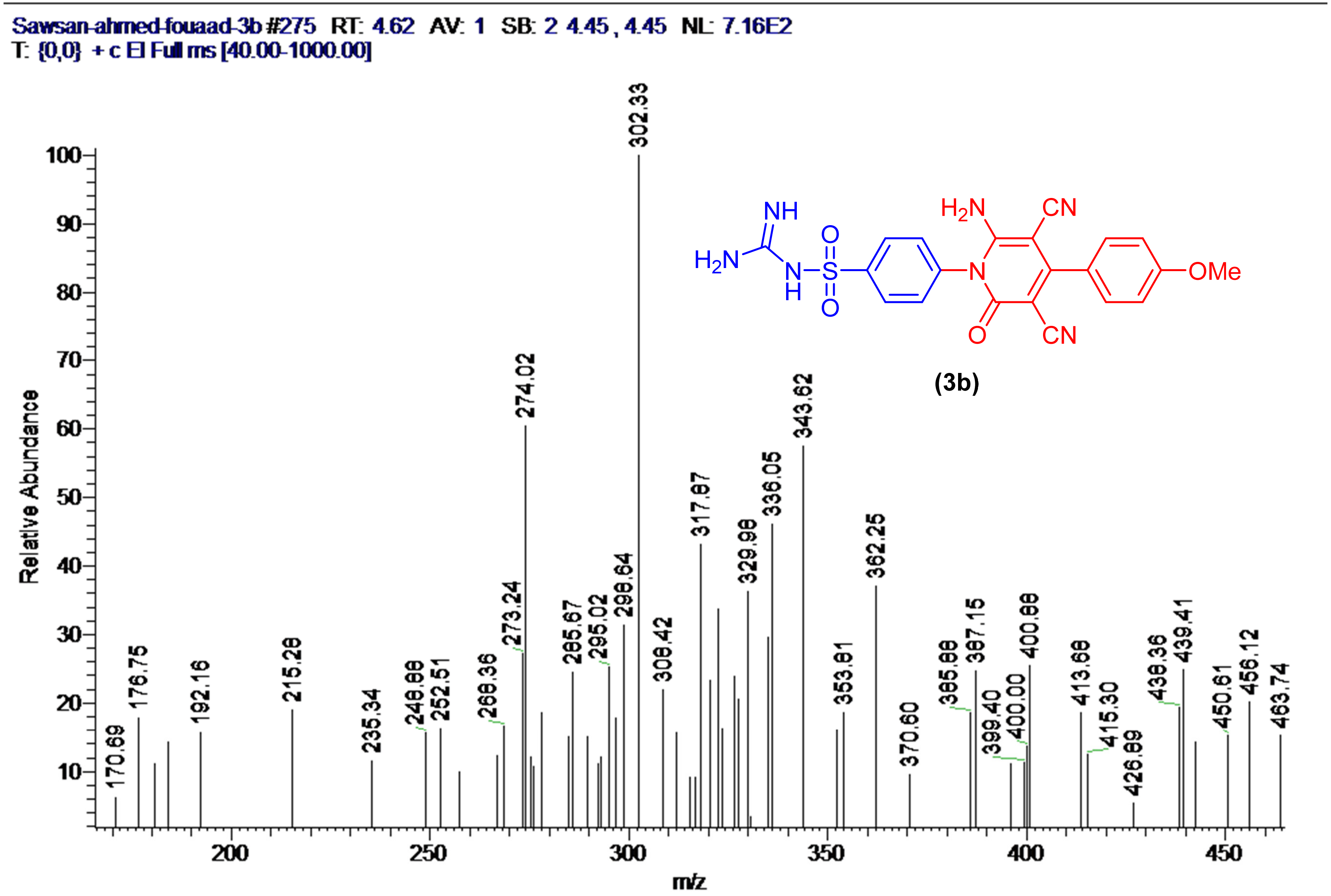
Sawsan-ahmed-fouaad-3b #275 RT: 4.62 AV: 1 SB: 2 4.45 , 4.45 NL: 7.16E2
T: {0,0} + c EI Full ms [40.00-1000.00]
Relative Abundance
m/z
302.33
274.02
343.62
336.05
317.87
362.25
329.98
298.64
400.88
439.41
387.15
456.12
463.74
450.61
438.36
413.68
385.88
415.30
399.40
400.00
426.69
370.60
353.81
308.42
295.02
285.67
273.24
268.36
252.51
248.88
235.34
215.28
192.16
176.75
170.69
NH
$H_2N$
N
H
S
O
O
$H_2N$
CN
CN
O
OMe
(3b)

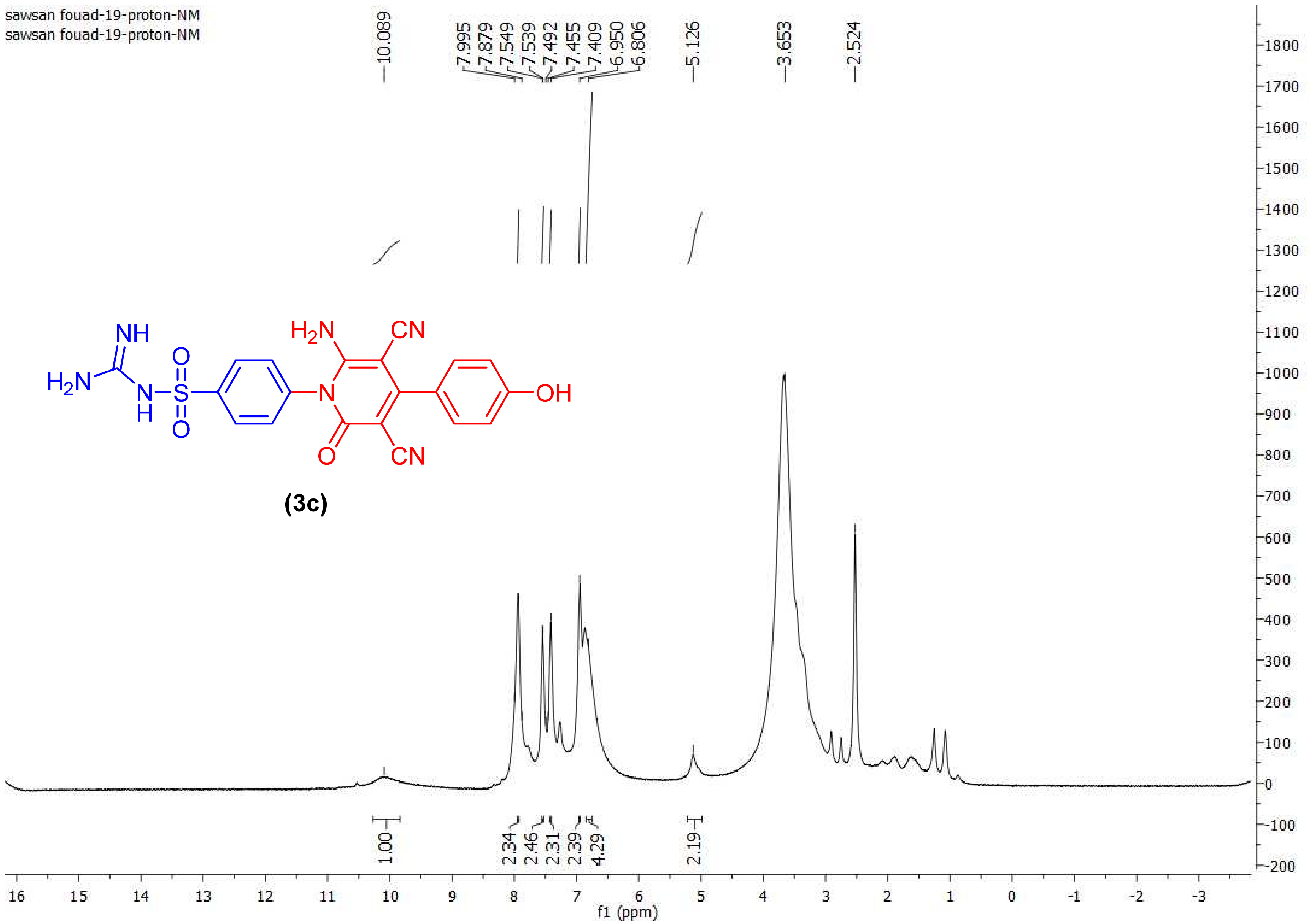

sawsan fouad-19-proton-NM
sawsan fouad-19-proton-NM
10.089
7.995
7.879
7.549
7.539
7.492
7.455
7.409
6.950
6.806
5.126
3.653
2.524
(3c)
1.00
2.34
2.46
2.31
2.39
4.29
2.19
f1 (ppm)

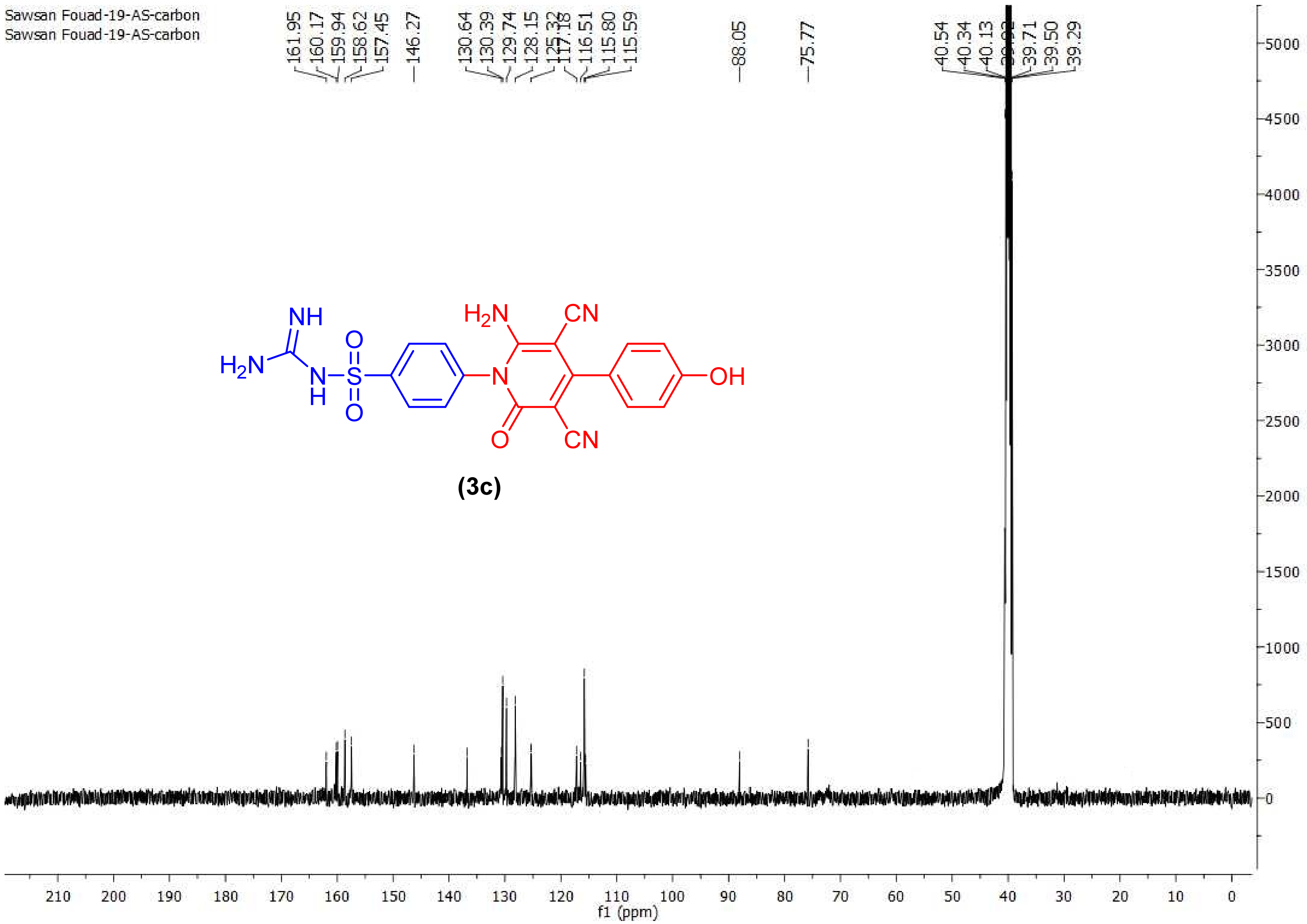
Sawsan Fouad-19-AS-carbon
Sawsan Fouad-19-AS-carbon
161.95
160.17
159.94
158.62
157.45
146.27
130.64
130.39
129.74
128.15
125.32
117.18
116.51
115.80
115.59
88.05
75.77
40.54
40.34
40.13
39.71
39.50
39.29
NH
$H_2N$
N
H
S
O
O
$H_2N$
CN
N
O
CN
OH
f1 (ppm)

**(3c)**

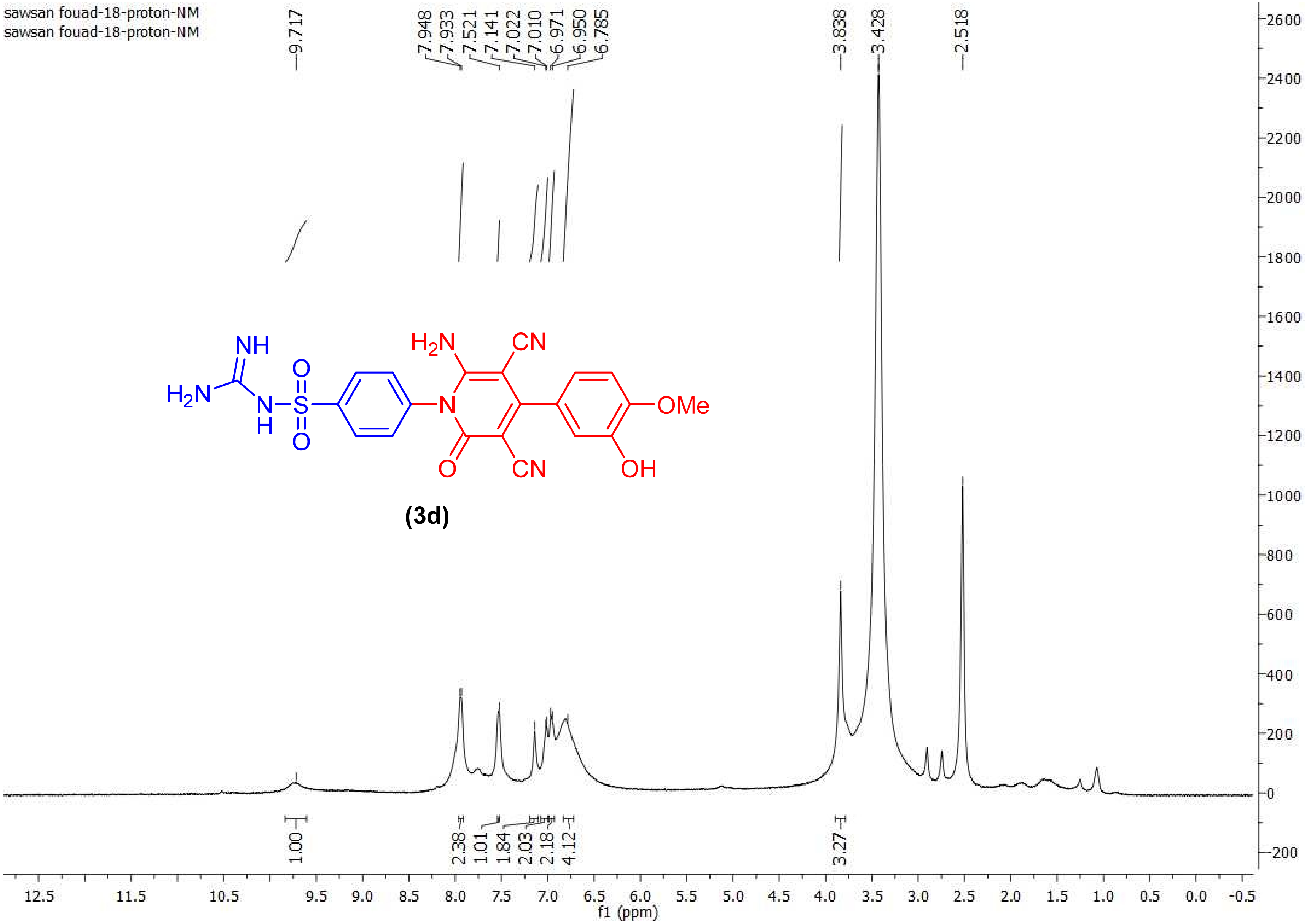
sawsan fouad-18-proton-NM
sawsan fouad-18-proton-NM
9.717
7.948
7.933
7.521
7.141
7.022
7.010
6.971
6.950
6.785
3.838
3.428
2.518
NH
H2N
N-S
H
O
O
H2N
CN
N
O
CN
OMe
OH
(3d)
1.00
2.38
1.01
1.84
2.03
2.18
4.12
3.27
f1 (ppm)

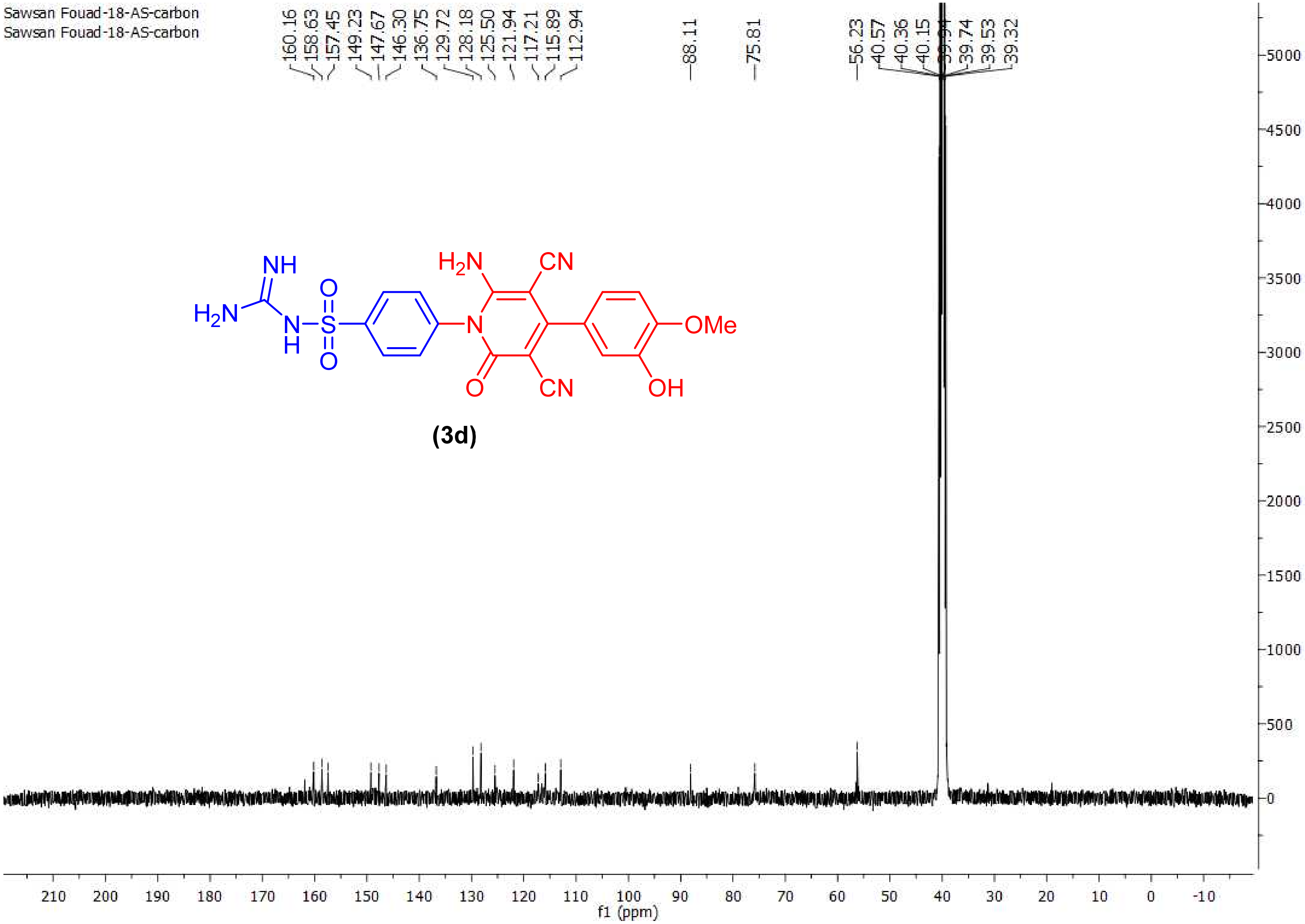

Sawsan Fouad-18-AS-carbon
Sawsan Fouad-18-AS-carbon
160.16
158.63
157.45
149.23
147.67
146.30
136.75
129.72
128.18
125.50
121.94
117.21
115.89
112.94
88.11
75.81
56.23
40.57
40.36
40.15
39.74
39.53
39.32
NH
H2N
N
H
S
O
O
H2N
CN
N
O
CN
OMe
OH
(3d)
f1 (ppm)

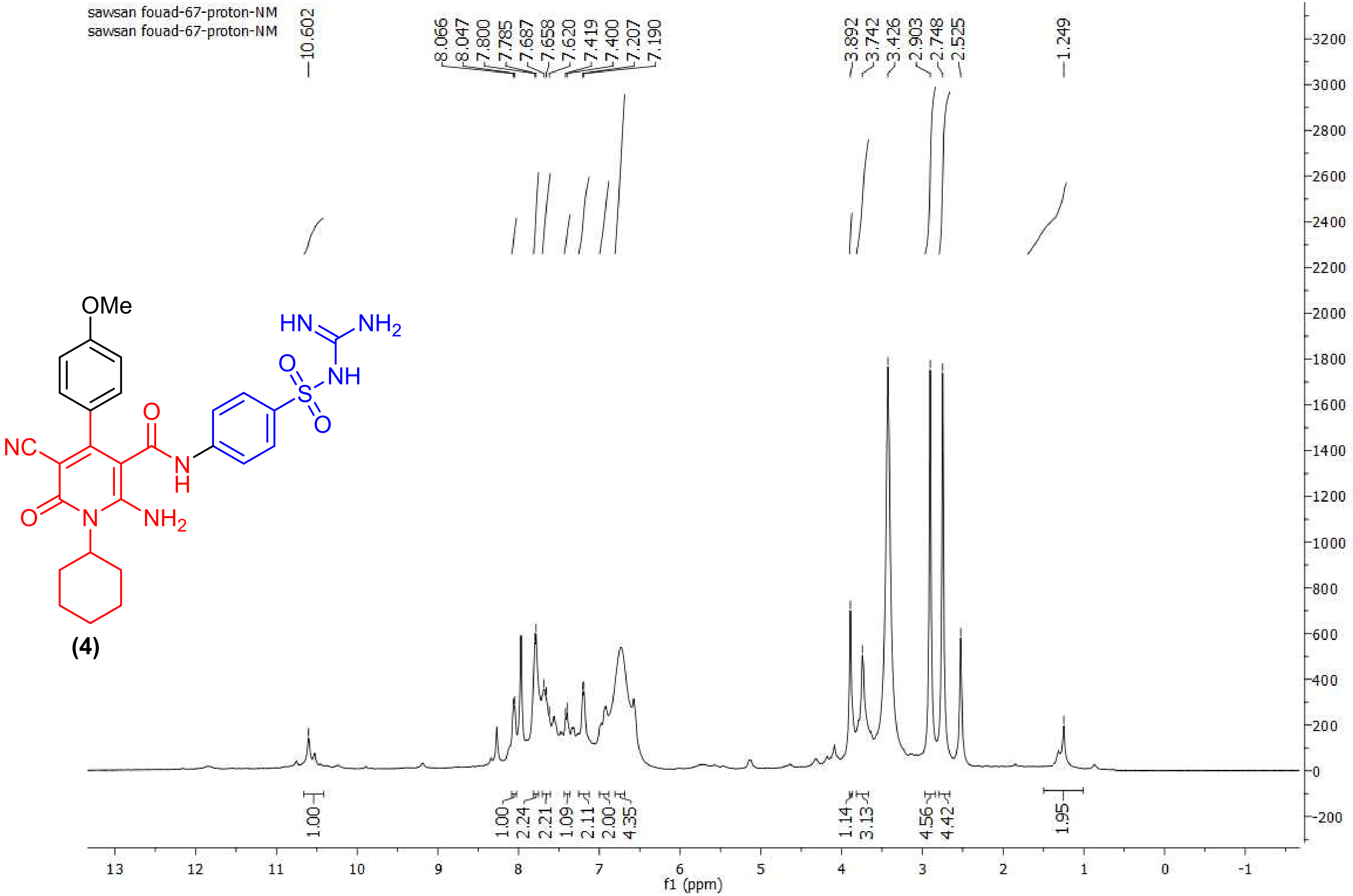
sawsan fouad-67-proton-NM
sawsan fouad-67-proton-NM
10.602
8.066
8.047
7.800
7.785
7.687
7.658
7.620
7.419
7.400
7.207
7.190
3.892
3.742
3.426
2.903
2.748
2.525
1.249
OMe
HN
NH2
O
S
NH
O
O
NC
N
H
O
N
NH2
1.00
1.00
2.24
2.21
1.09
2.11
2.00
4.35
1.14
3.13
4.56
4.42
1.95
f1 (ppm)

**(4)**

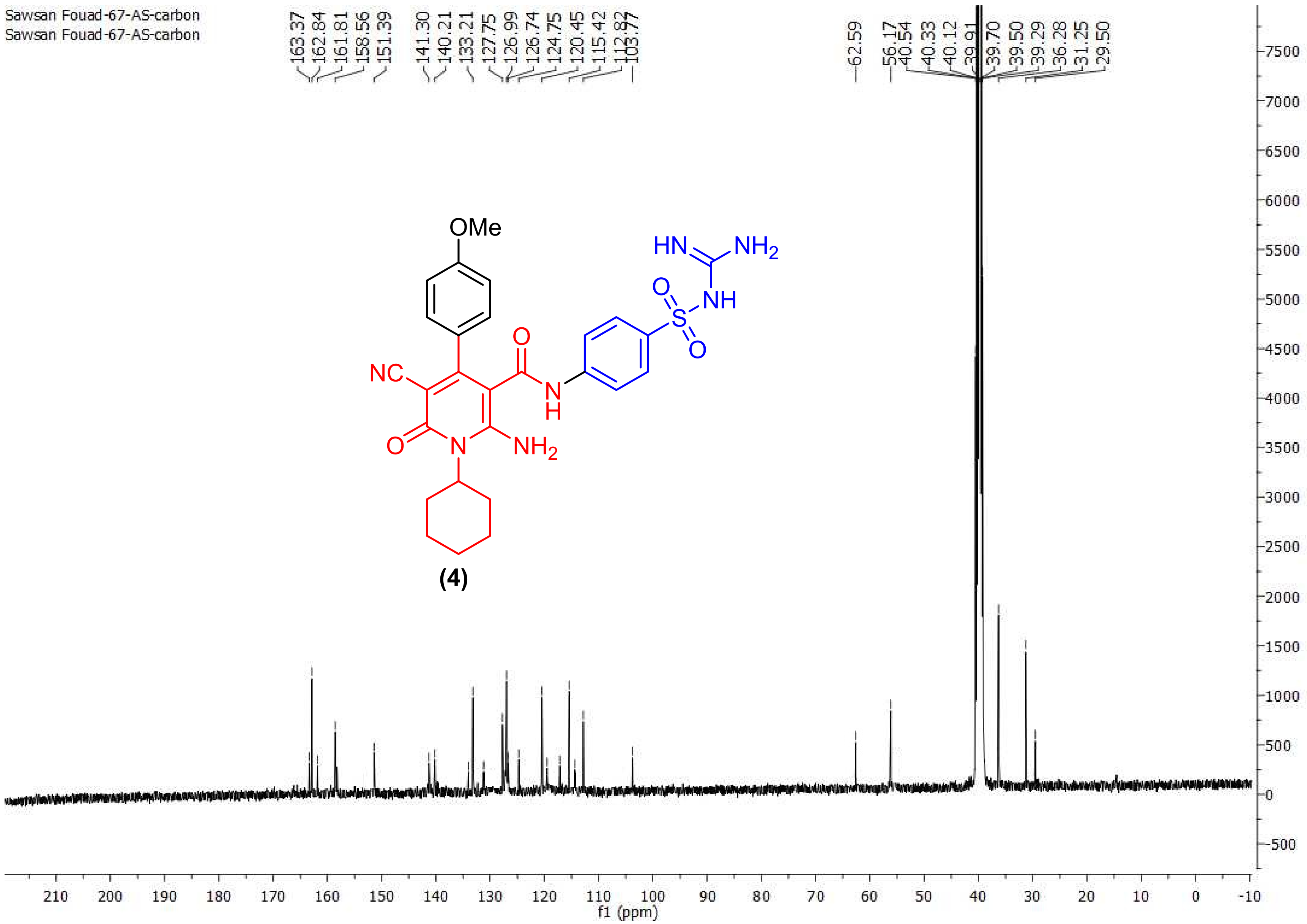
Sawsan Fouad-67-AS-carbon
Sawsan Fouad-67-AS-carbon
163.37
162.84
161.81
158.56
151.39
141.30
140.21
133.21
127.75
126.99
126.74
124.75
120.45
115.42
112.82
103.77
62.59
56.17
40.54
40.33
40.12
39.91
39.70
39.50
39.29
36.28
31.25
29.50
OMe
HN
NH2
O
S
NH
O
O
NC
N
H
O
N
NH2
(4)
f1 (ppm)

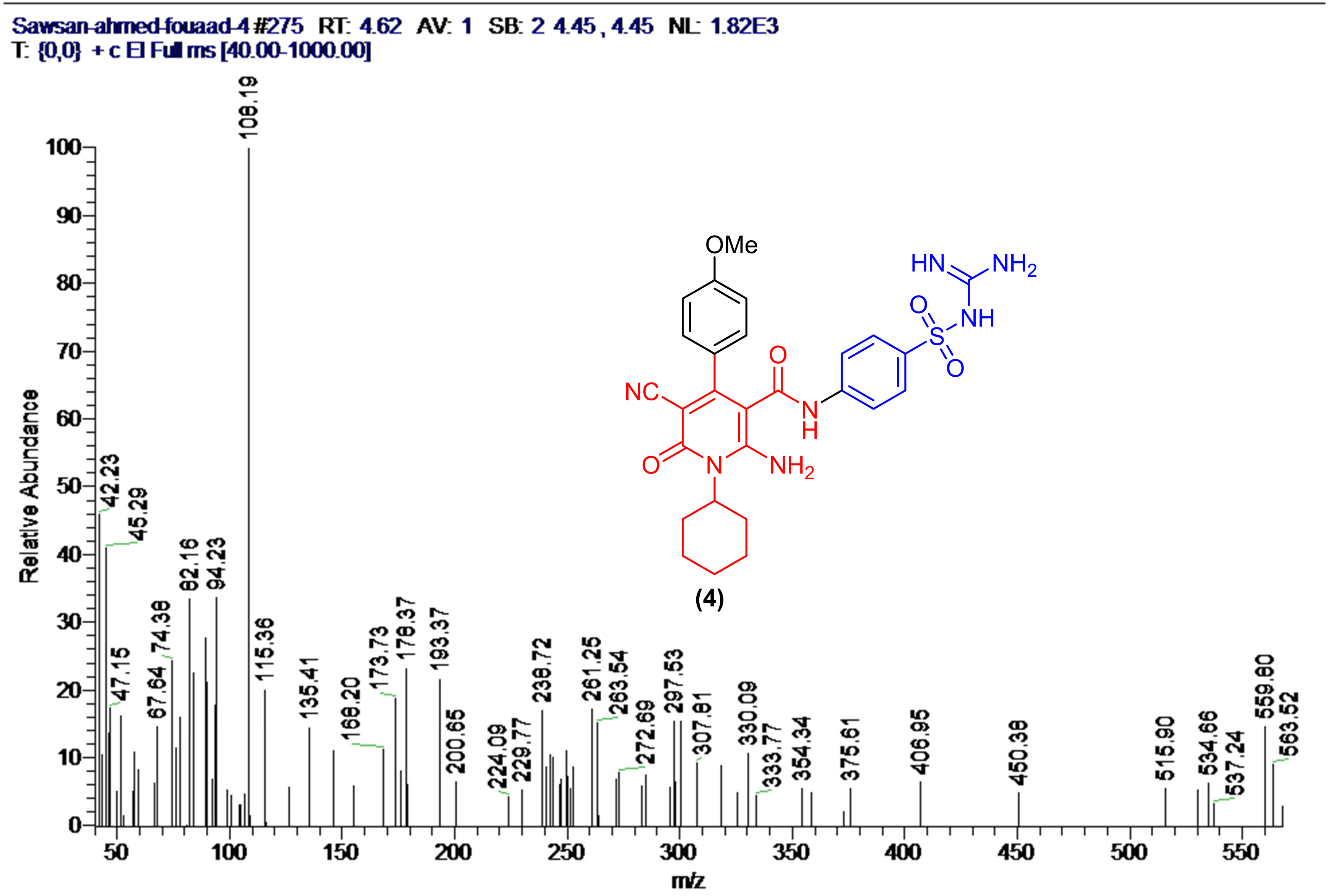
Sawsan-ahmed-fouaad-4 #275 RT: 4.62 AV: 1 SB: 2 4.45 , 4.45 NL: 1.82E3
T: {0,0} + c EI Full ms [40.00-1000.00]
Relative Abundance
m/z
108.19
42.23
45.29
47.15
67.64
74.38
82.16
94.23
115.36
135.41
168.20
173.73
178.37
193.37
200.65
224.09
229.77
238.72
261.25
263.54
272.69
297.53
307.61
330.09
333.77
354.34
375.61
406.95
450.38
515.90
534.66
537.24
559.80
563.52
OMe
NC
O
N
NH
H
NH2
HN
NH2
O
S
NH
O
(4)

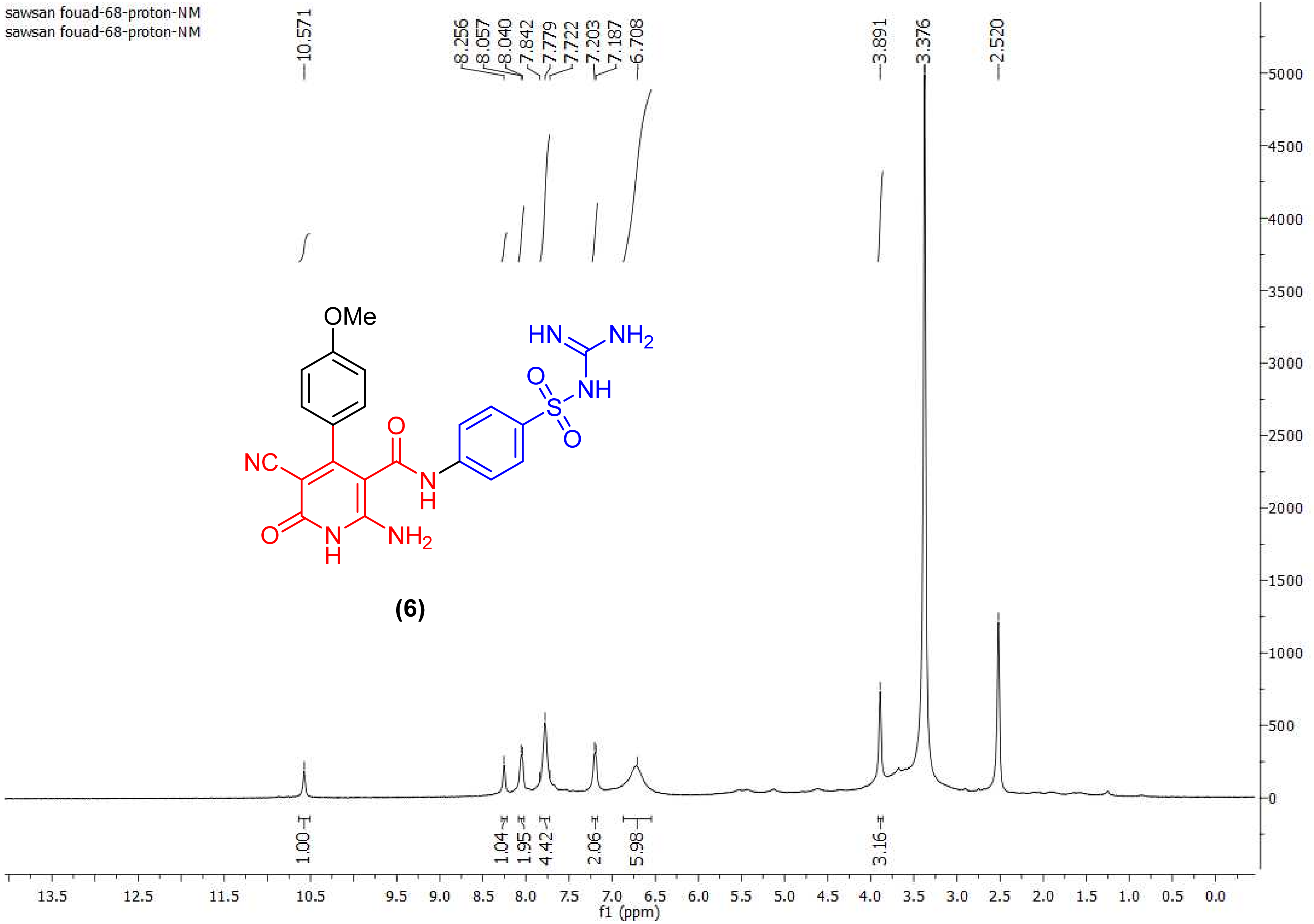
sawsan fouad-68-proton-NM
sawsan fouad-68-proton-NM
10.571
8.256
8.057
8.040
7.842
7.779
7.722
7.203
7.187
6.708
3.891
3.376
2.520
OMe
HN
NH2
O
S
NH
O
O
NC
N
H
O
N
H
NH2
1.00
1.04
1.95
4.42
2.06
5.98
3.16
f1 (ppm)

**(6)**

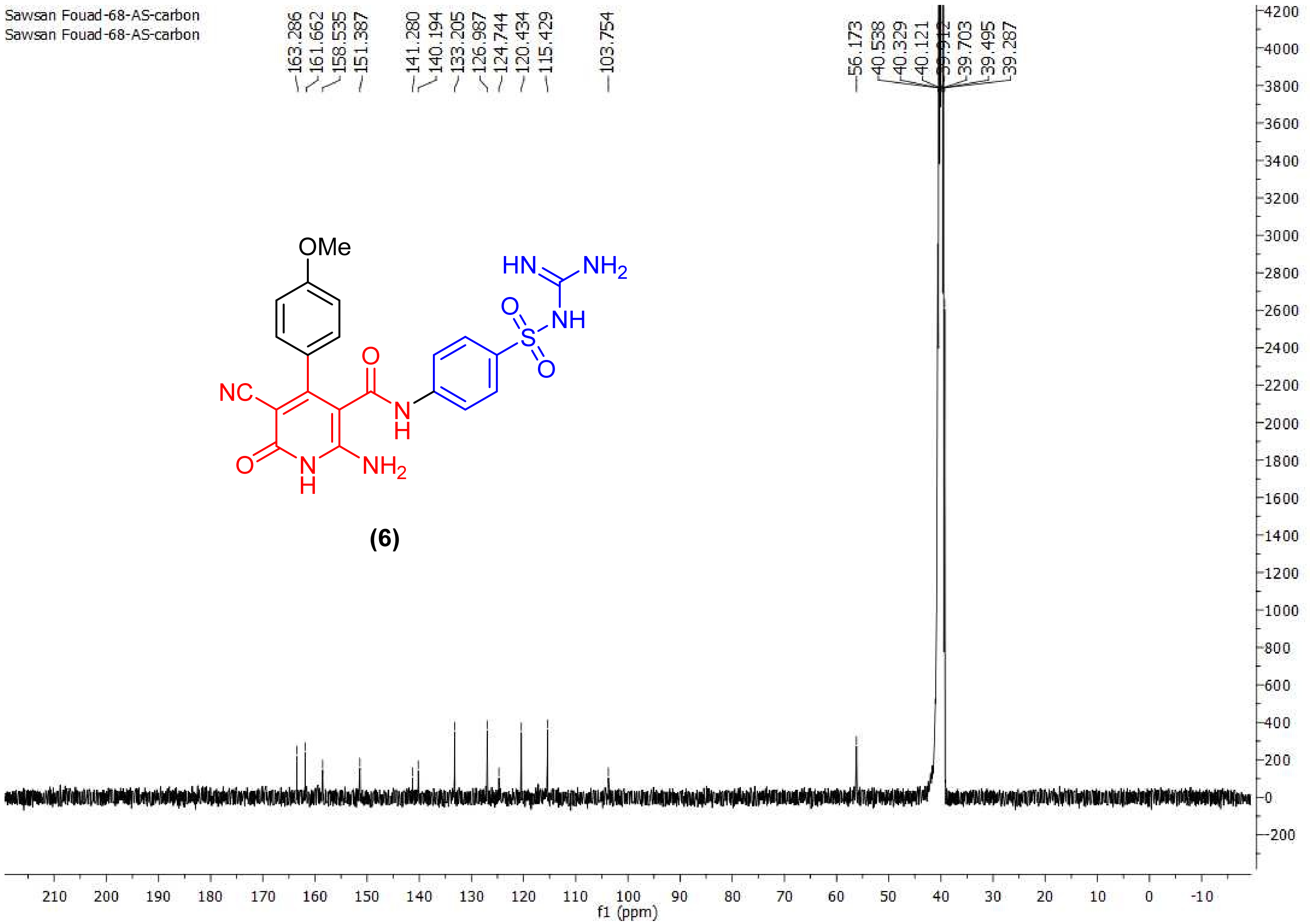
Sawsan Fouad-68-AS-carbon
Sawsan Fouad-68-AS-carbon
163.286
161.662
158.535
151.387
141.280
140.194
133.205
126.987
124.744
120.434
115.429
103.754
56.173
40.538
40.329
40.121
39.912
39.703
39.495
39.287
OMe
HN
NH2
O
S
NH
O
O
NC
N
H
O
N
H
NH2
(6)
f1 (ppm)

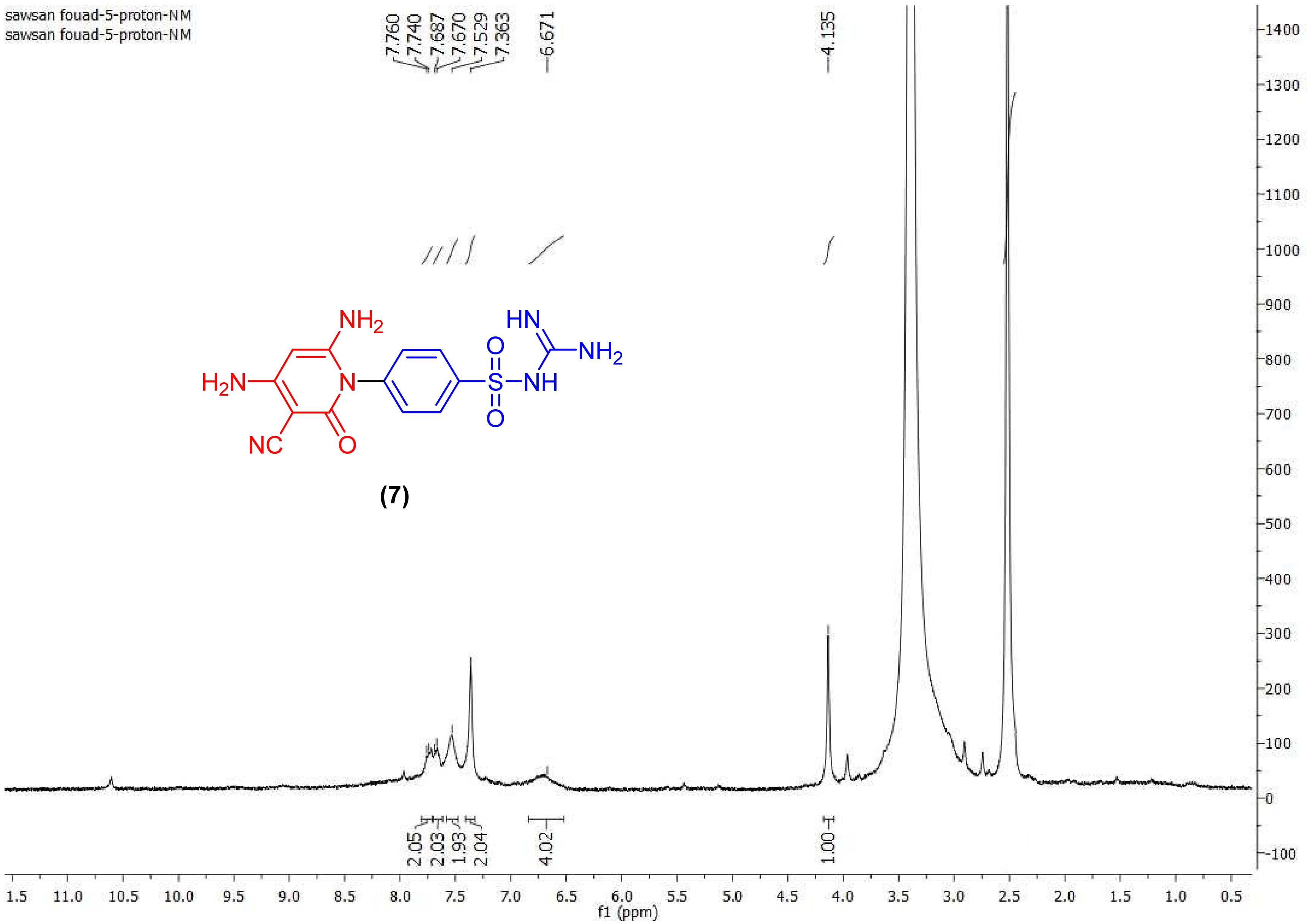
sawsan fouad-5-proton-NM
sawsan fouad-5-proton-NM
7.760
7.740
7.687
7.670
7.529
7.363
6.671
4.135
$NH_2$
HN
$NH_2$
$H_2N$
N
O
S
NH
O
NC
O
(7)
2.05
2.03
1.93
2.04
4.02
1.00
f1 (ppm)

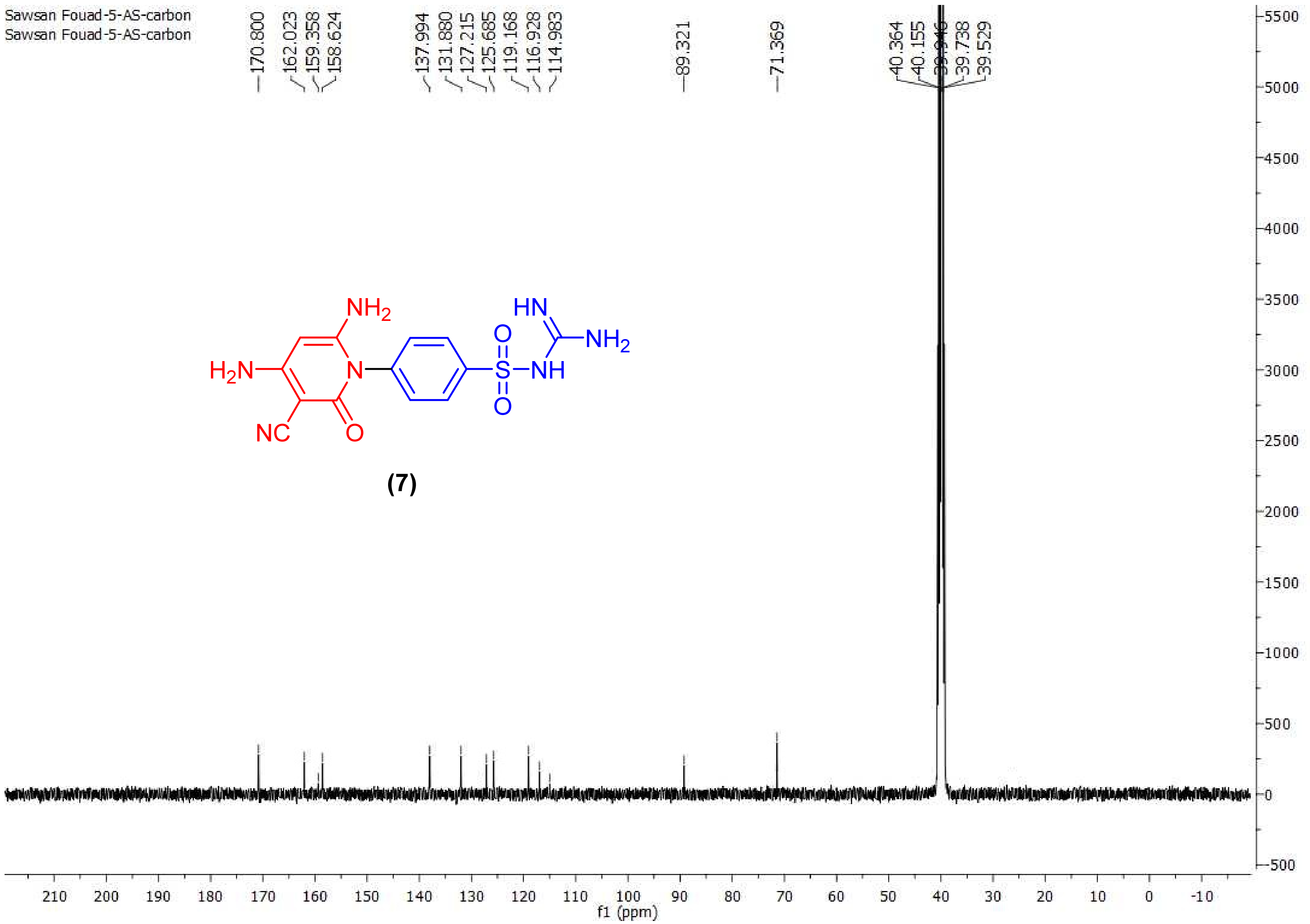
Sawsan Fouad-5-AS-carbon
Sawsan Fouad-5-AS-carbon
170.800
162.023
159.358
158.624
137.994
131.880
127.215
125.685
119.168
116.928
114.983
89.321
71.369
40.364
40.155
39.946
39.738
39.529
NH2
H2N
N
NC
O
HN
NH2
O
S
NH
O
(7)
f1 (ppm)

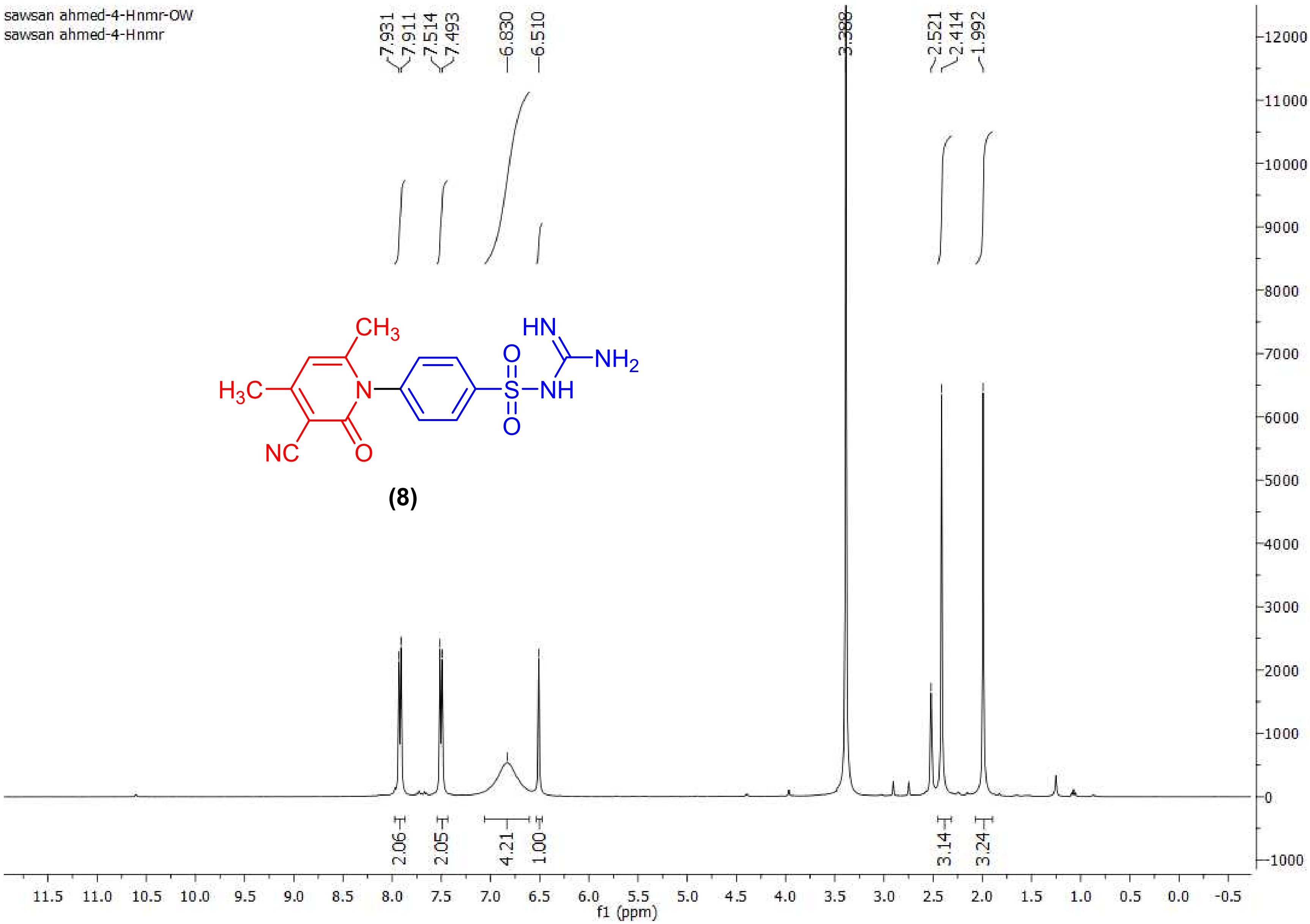
sawsan ahmed-4-Hnmr-OW
sawsan ahmed-4-Hnmr
7.931
7.911
7.514
7.493
6.830
6.510
3.388
2.521
2.414
1.992
2.06
2.05
4.21
1.00
3.14
3.24
f1 (ppm)
(8)

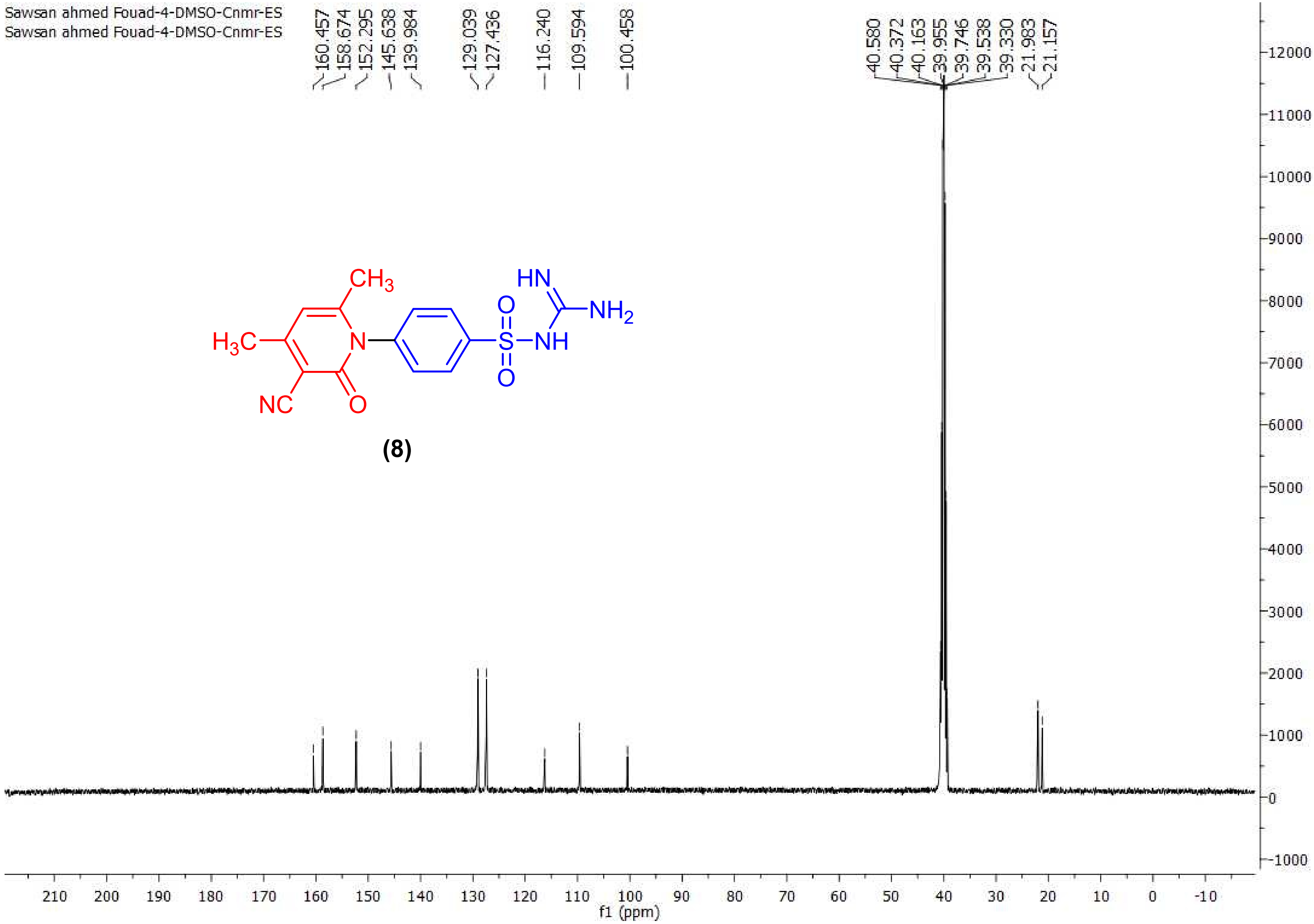
Sawsan ahmed Fouad-4-DMSO-Cnmr-ES
Sawsan ahmed Fouad-4-DMSO-Cnmr-ES
160.457
158.674
152.295
145.638
139.984
129.039
127.436
116.240
109.594
100.458
40.580
40.372
40.163
39.955
39.746
39.538
39.330
21.983
21.157
CH3
H3C
N
NC
O
HN
NH2
O
S
NH
O
(8)
f1 (ppm)

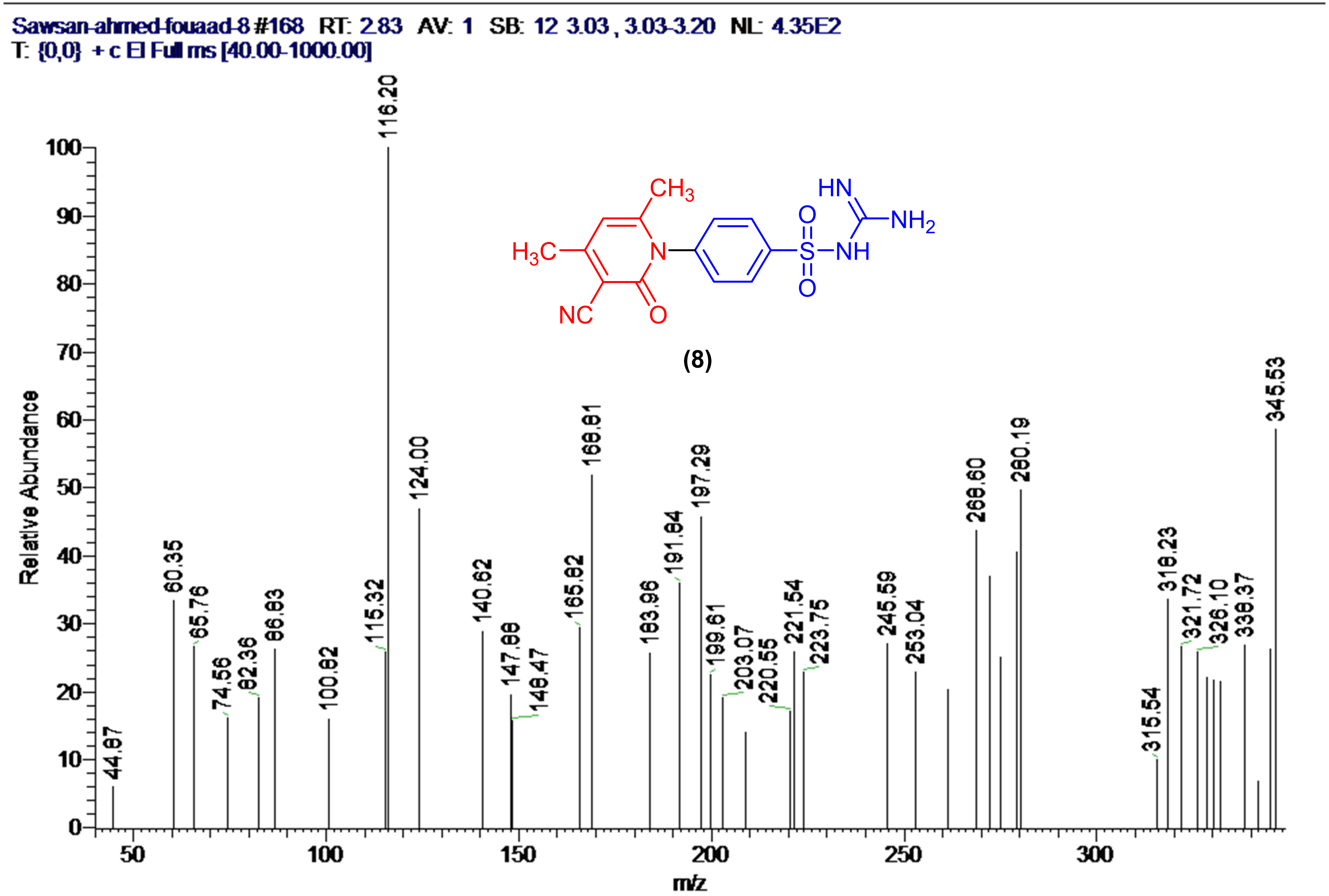
Sawsan-ahmed-fouaad-8 #168 RT: 2.83 AV: 1 SB: 12 3.03 , 3.03-3.20 NL: 4.35E2
T: {0,0} + c EI Full ms [40.00-1000.00]
Relative Abundance
m/z
(8)
116.20
124.00
168.81
197.29
280.19
268.60
345.53
191.84
318.23
60.35
65.76
74.56
82.36
86.83
100.82
115.32
140.62
147.88
148.47
165.82
183.96
199.61
203.07
220.55
221.54
223.75
245.59
253.04
315.54
321.72
326.10
338.37

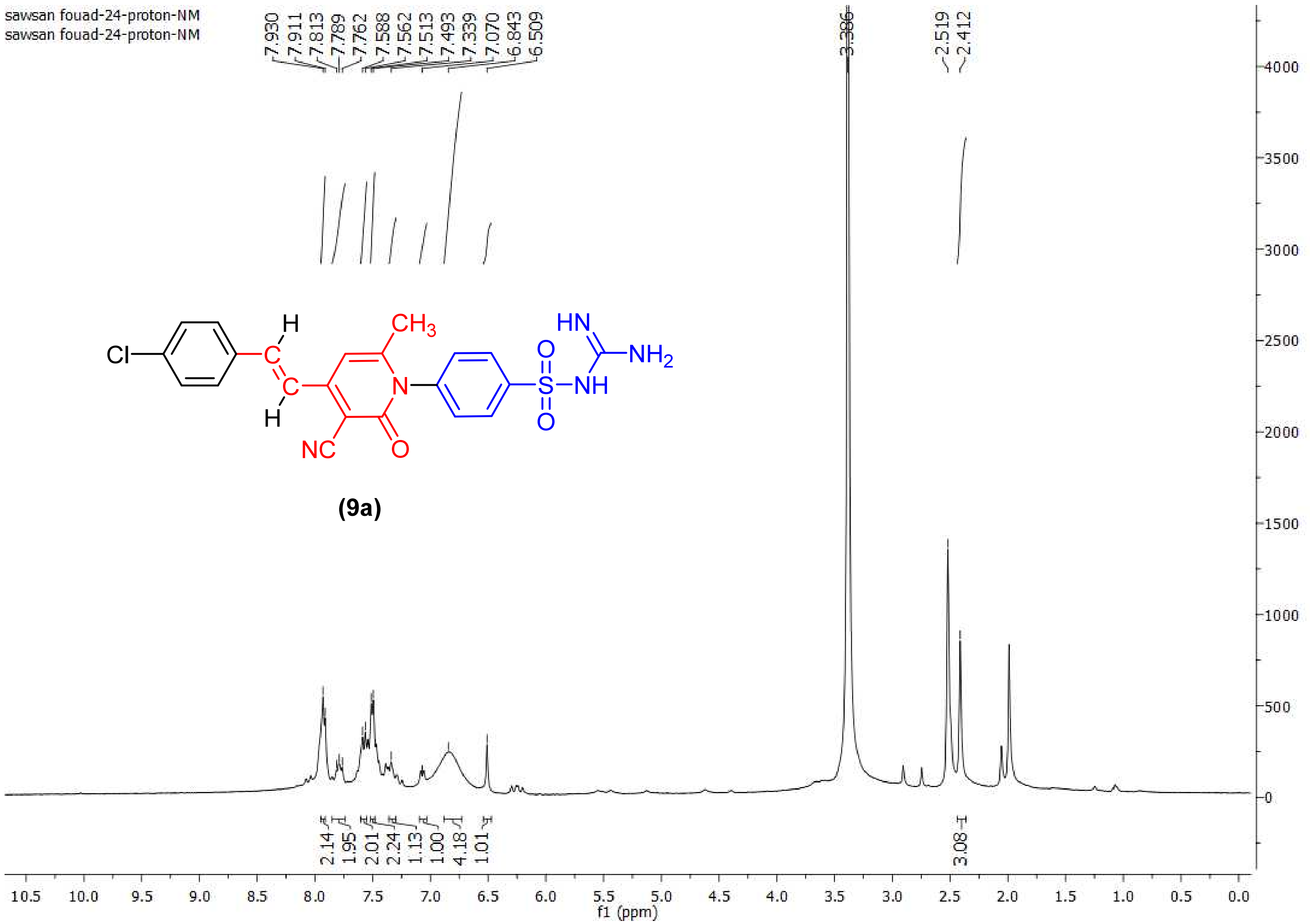
sawsan fouad-24-proton-NM
sawsan fouad-24-proton-NM
7.930
7.911
7.813
7.789
7.762
7.588
7.562
7.513
7.493
7.339
7.070
6.843
6.509
3.386
2.519
2.412
2.14
1.95
2.01
2.24
1.13
1.00
4.18
1.01
3.08
f1 (ppm)

**(9a)**

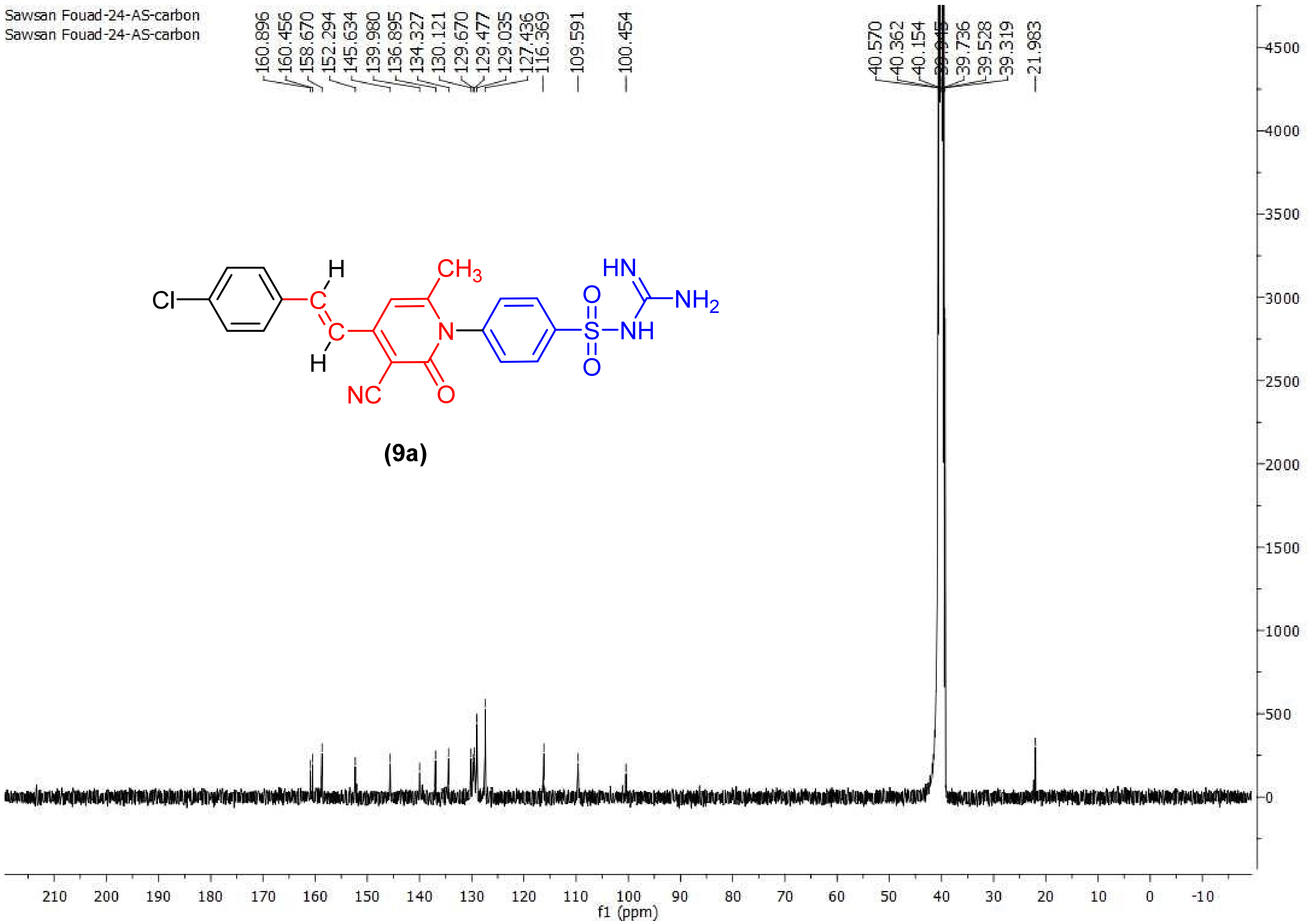
Sawsan Fouad-24-AS-carbon
Sawsan Fouad-24-AS-carbon
160.896
160.456
158.670
152.294
145.634
139.980
136.895
134.327
130.121
129.670
129.477
129.035
127.436
116.369
109.591
100.454
40.570
40.362
40.154
39.736
39.528
39.319
21.983
(9a)
f1 (ppm)

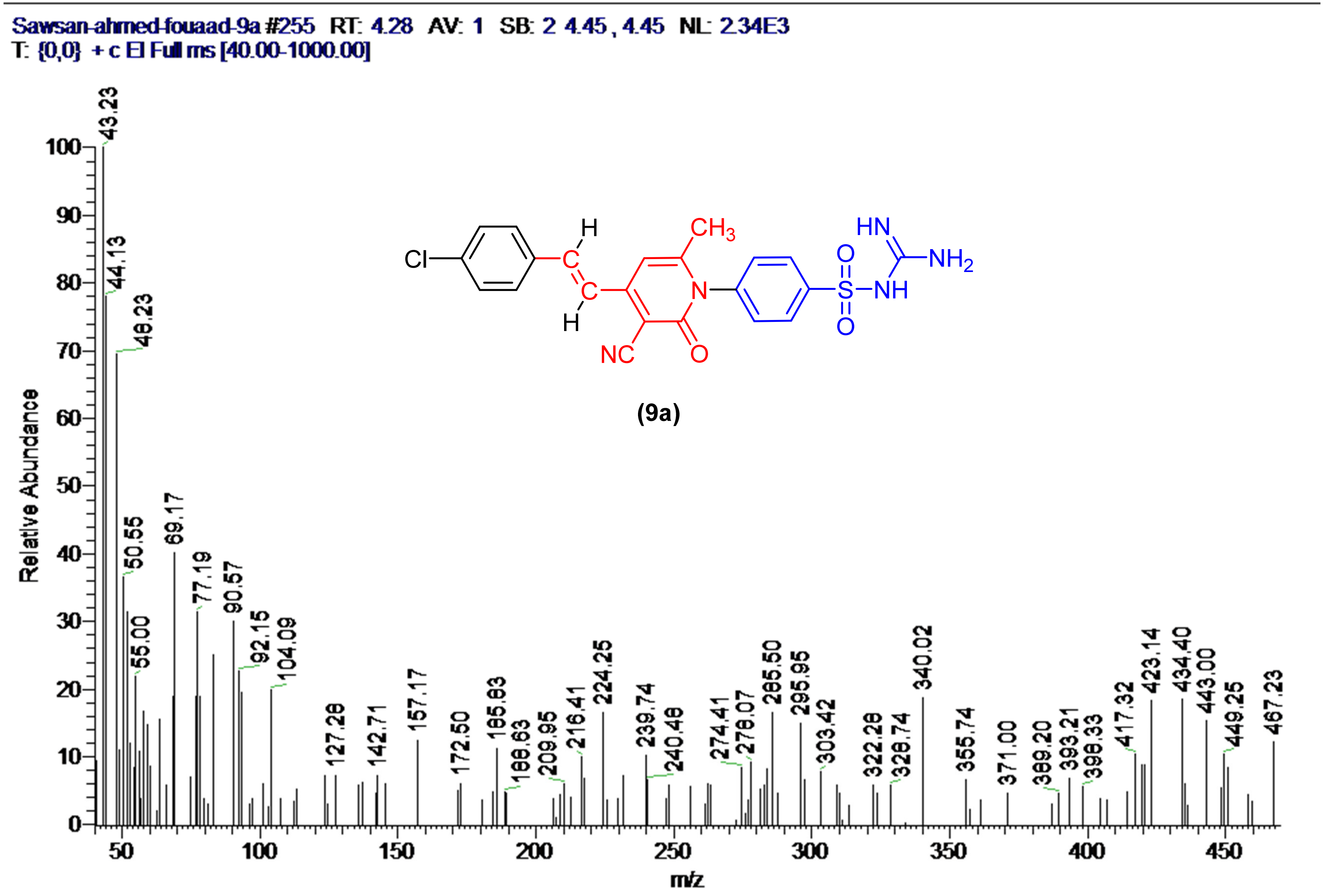
Sawsan-ahmed-fouaad-9a #255 RT: 4.28 AV: 1 SB: 2 4.45 , 4.45 NL: 2.34E3
T: {0,0} + c EI Full ms [40.00-1000.00]
Relative Abundance
m/z
43.23
44.13
46.23
50.55
55.00
69.17
77.19
90.57
92.15
104.09
127.28
142.71
157.17
172.50
185.83
188.63
209.95
216.41
224.25
239.74
240.48
274.41
276.07
285.50
295.95
303.42
322.28
326.74
340.02
355.74
371.00
389.20
393.21
398.33
417.32
423.14
434.40
443.00
449.25
467.23
Cl
H
C
C
H
CH3
NC
N
O
O
S
O
NH
HN
NH2
(9a)

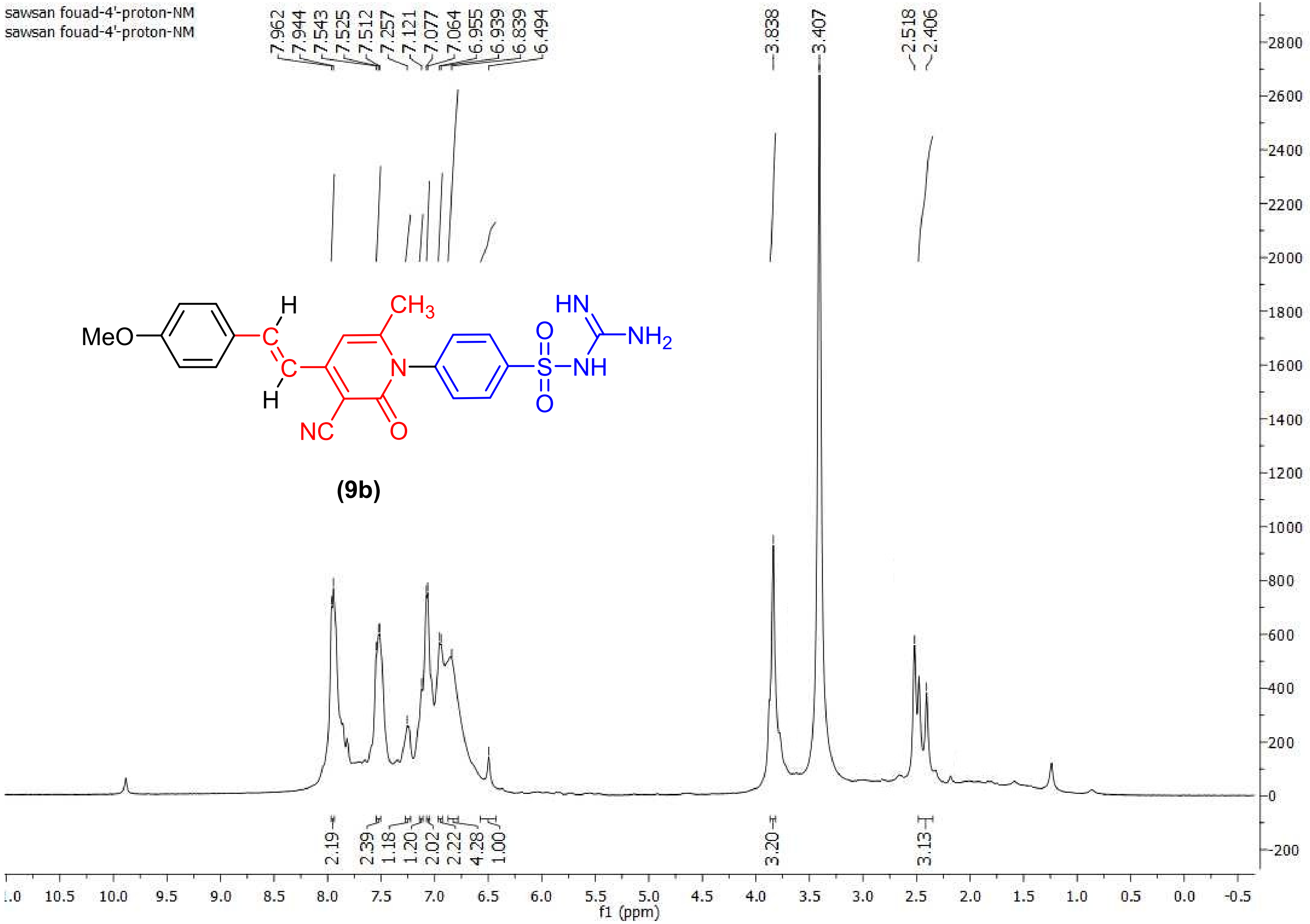
sawsan fouad-4'-proton-NM
sawsan fouad-4'-proton-NM
7.962 7.944 7.543 7.525 7.512 7.257 7.121 7.077 7.064 6.955 6.939 6.839 6.494
3.838
3.407
2.518 2.406
MeO
H
CH3
HN
NH2
O
S
NH
N
C
H
NC
O
(9b)
2.19 2.39 1.18 1.20 2.02 2.22 4.28 1.00
3.20
3.13
f1 (ppm)

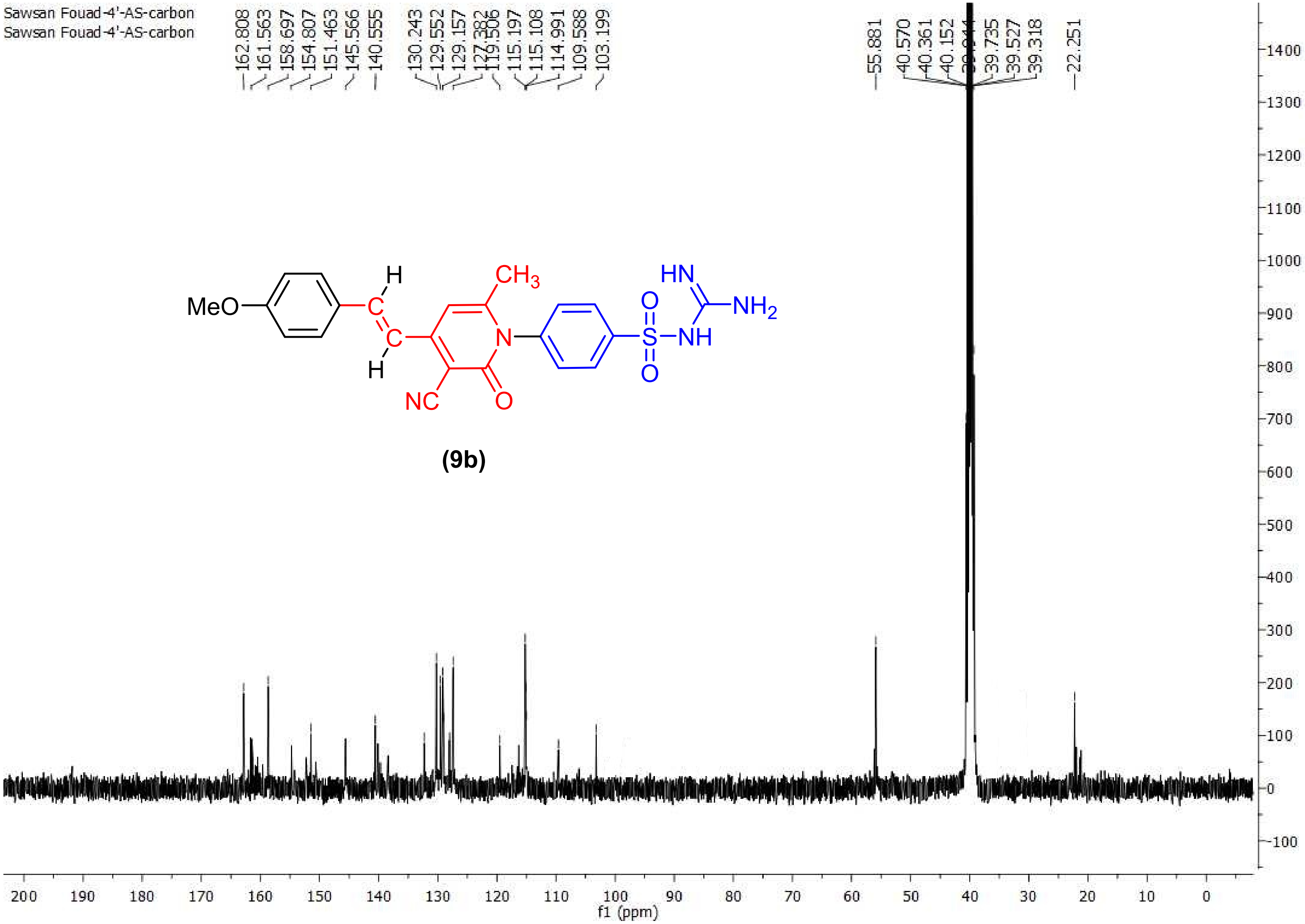
Sawsan Fouad-4'-AS-carbon
Sawsan Fouad-4'-AS-carbon
162.808
161.563
158.697
154.807
151.463
145.566
140.555
130.243
129.552
129.157
127.382
119.506
115.197
115.108
114.991
109.588
103.199
55.881
40.570
40.361
40.152
39.735
39.527
39.318
22.251
MeO
H
C
C
H
CH3
N
NC
O
O
S
O
NH
HN
NH2
f1 (ppm)

**(9b)**

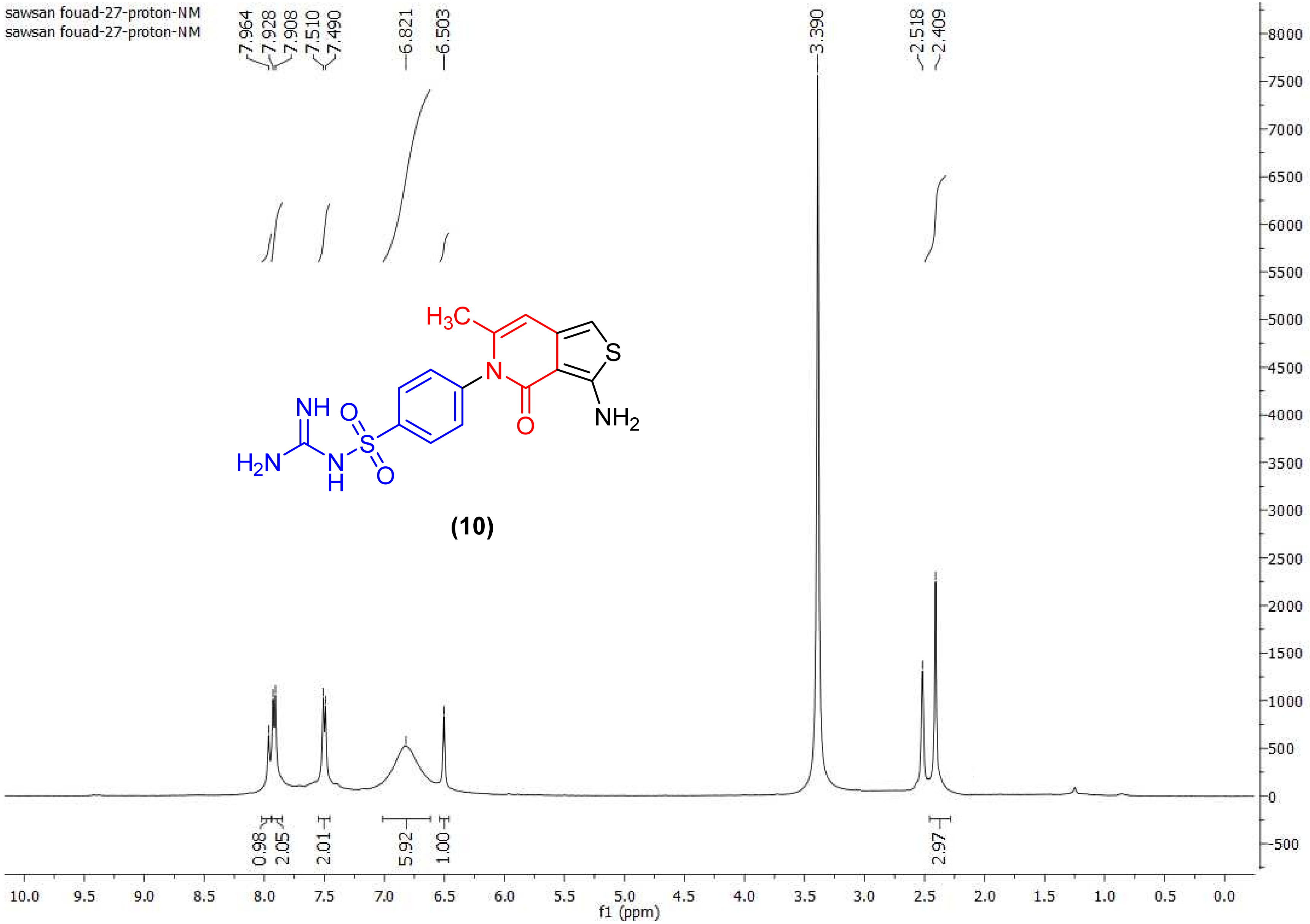
sawsan fouad-27-proton-NM
sawsan fouad-27-proton-NM
7.964
7.928
7.908
7.510
7.490
6.821
6.503
3.390
2.518
2.409
H3C
N
S
NH2
O
NH
O
S
H2N
N
H
O
(10)
0.98
2.05
2.01
5.92
1.00
2.97
f1 (ppm)

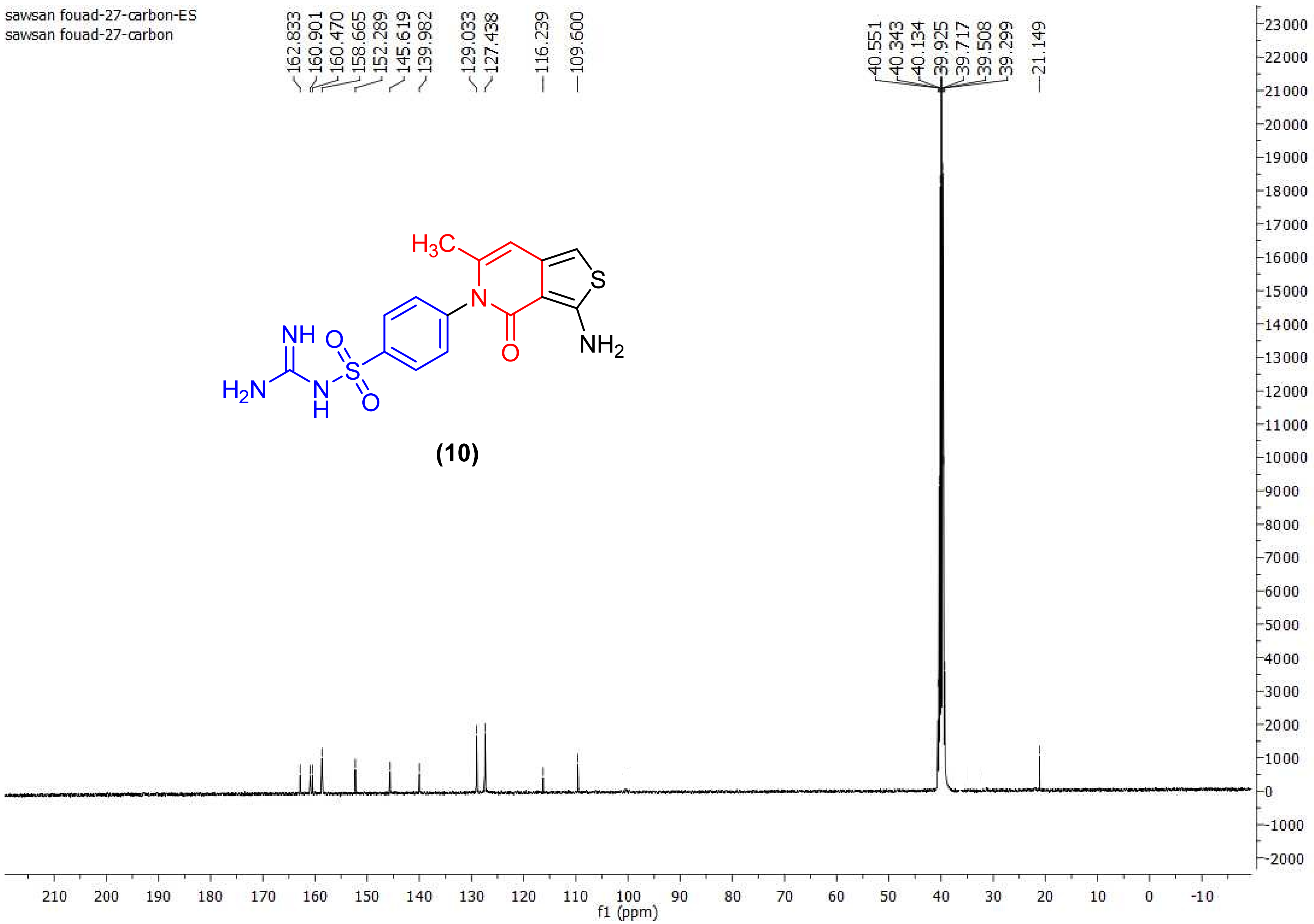

sawsan fouad-27-carbon-ES
sawsan fouad-27-carbon
162.833
160.901
160.470
158.665
152.289
145.619
139.982
129.033
127.438
116.239
109.600
40.551
40.343
40.134
39.925
39.717
39.508
39.299
21.149
(10)
f1 (ppm)

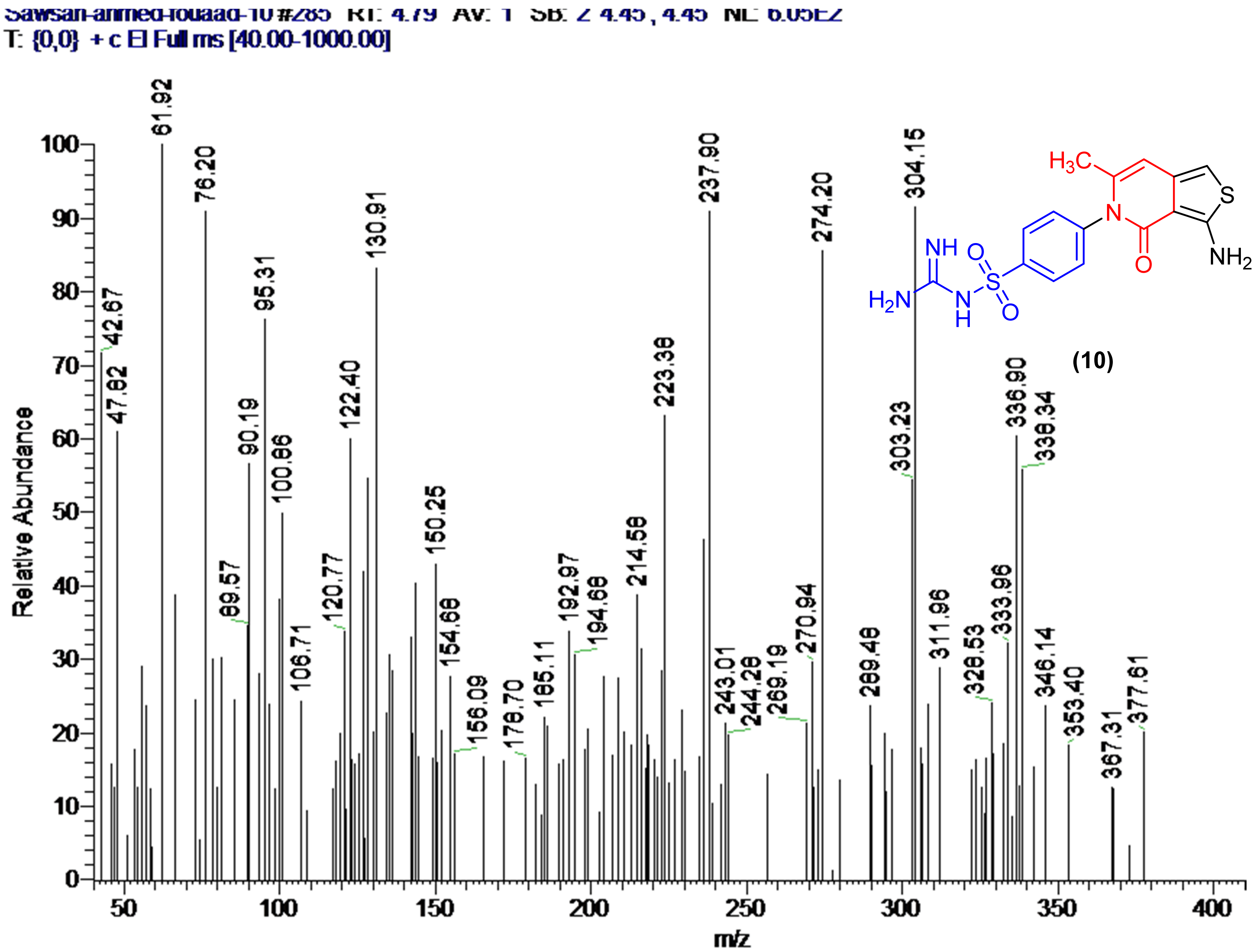
Sawsan-ahmed-fouaad-10 #285 RT: 4.79 AV: 1 SB: 2 4.45 , 4.45 NL: 6.05E2
T: {0,0} + c EI Full ms [40.00-1000.00]
Relative Abundance
m/z
61.92
76.20
95.31
130.91
237.90
274.20
304.15
223.38
336.90
338.34
303.23
42.67
47.82
90.19
100.86
122.40
150.25
H3C
N
S
O
NH2
NH
O
S
H2N
N
H
O
(10)

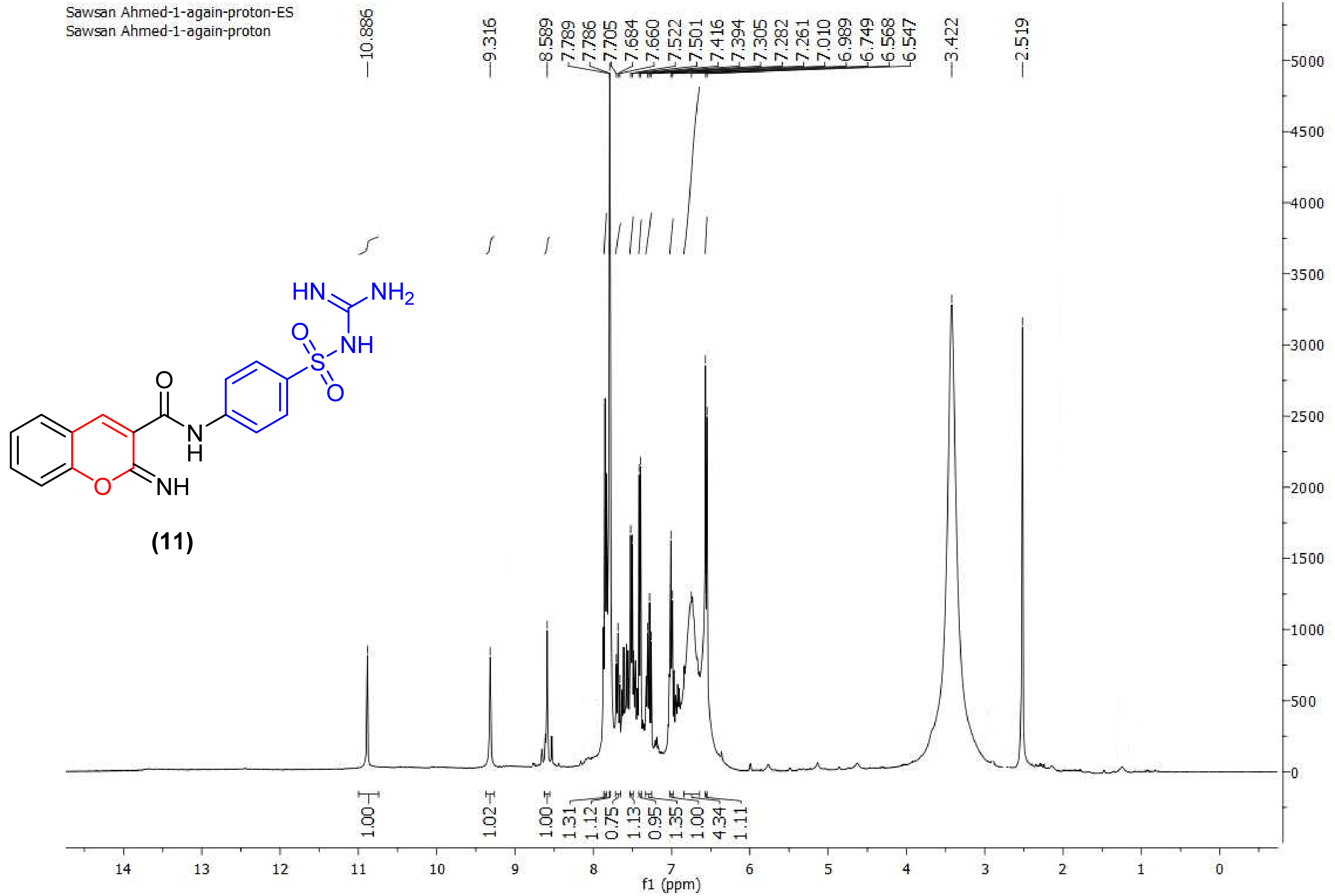
Sawsan Ahmed-1-again-proton-ES
Sawsan Ahmed-1-again-proton
10.886
9.316
8.589
7.789
7.786
7.705
7.684
7.660
7.522
7.501
7.416
7.394
7.305
7.282
7.261
7.010
6.989
6.749
6.568
6.547
3.422
2.519
HN
NH2
O
S
NH
O
O
N
H
O
NH
(11)
1.00
1.02
1.00
1.31
1.12
0.75
1.13
0.95
1.35
1.00
4.34
1.11
f1 (ppm)

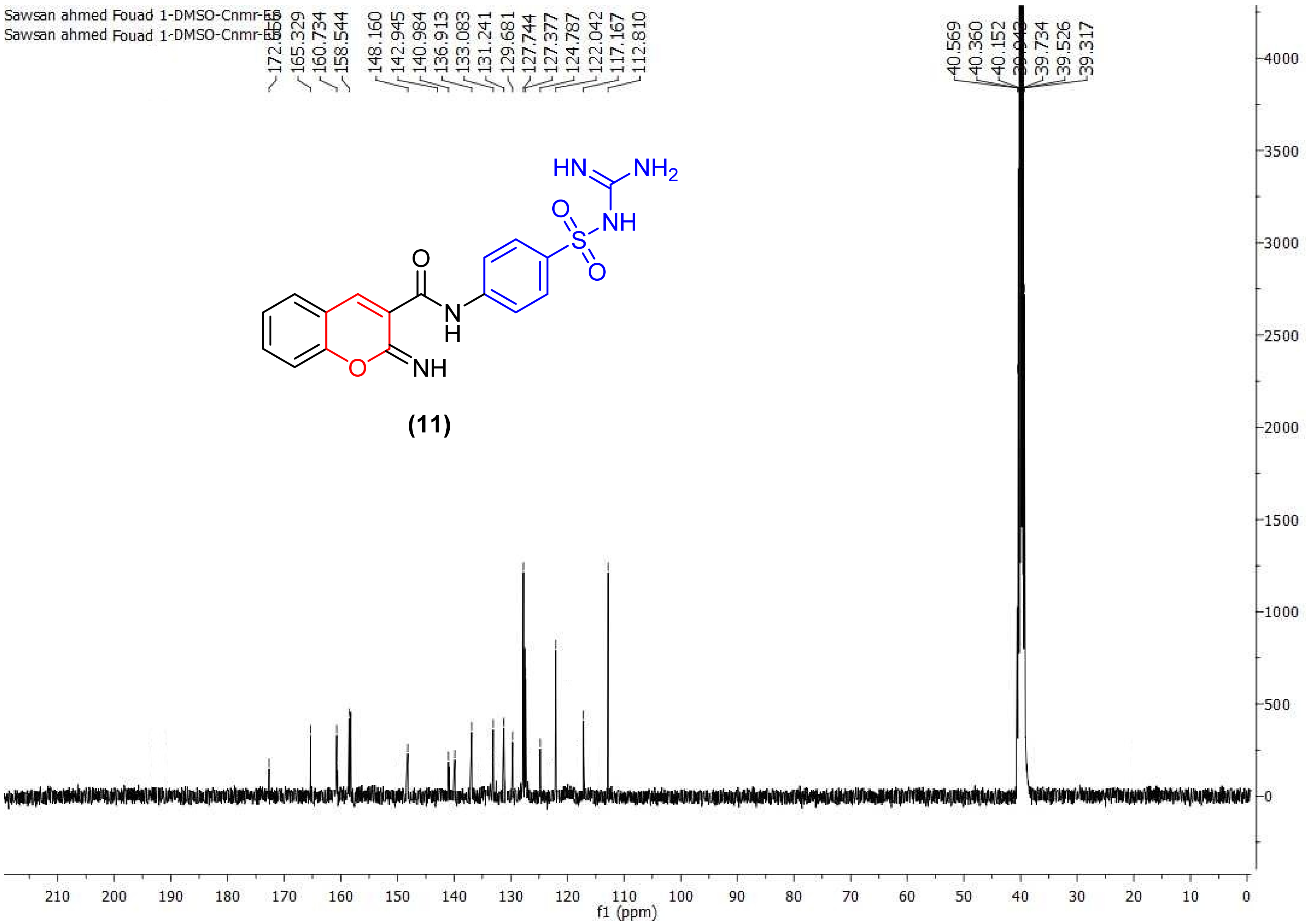
Sawsan ahmed Fouad 1-DMSO-Cnmr-E6
172.565
165.329
160.734
158.544
148.160
142.945
140.984
136.913
133.083
131.241
129.681
127.744
127.377
124.787
122.042
117.167
112.810
40.569
40.360
40.152
39.943
39.734
39.526
39.317
HN
NH2
O
S
NH
O
O
N
H
O
NH
(11)
f1 (ppm)

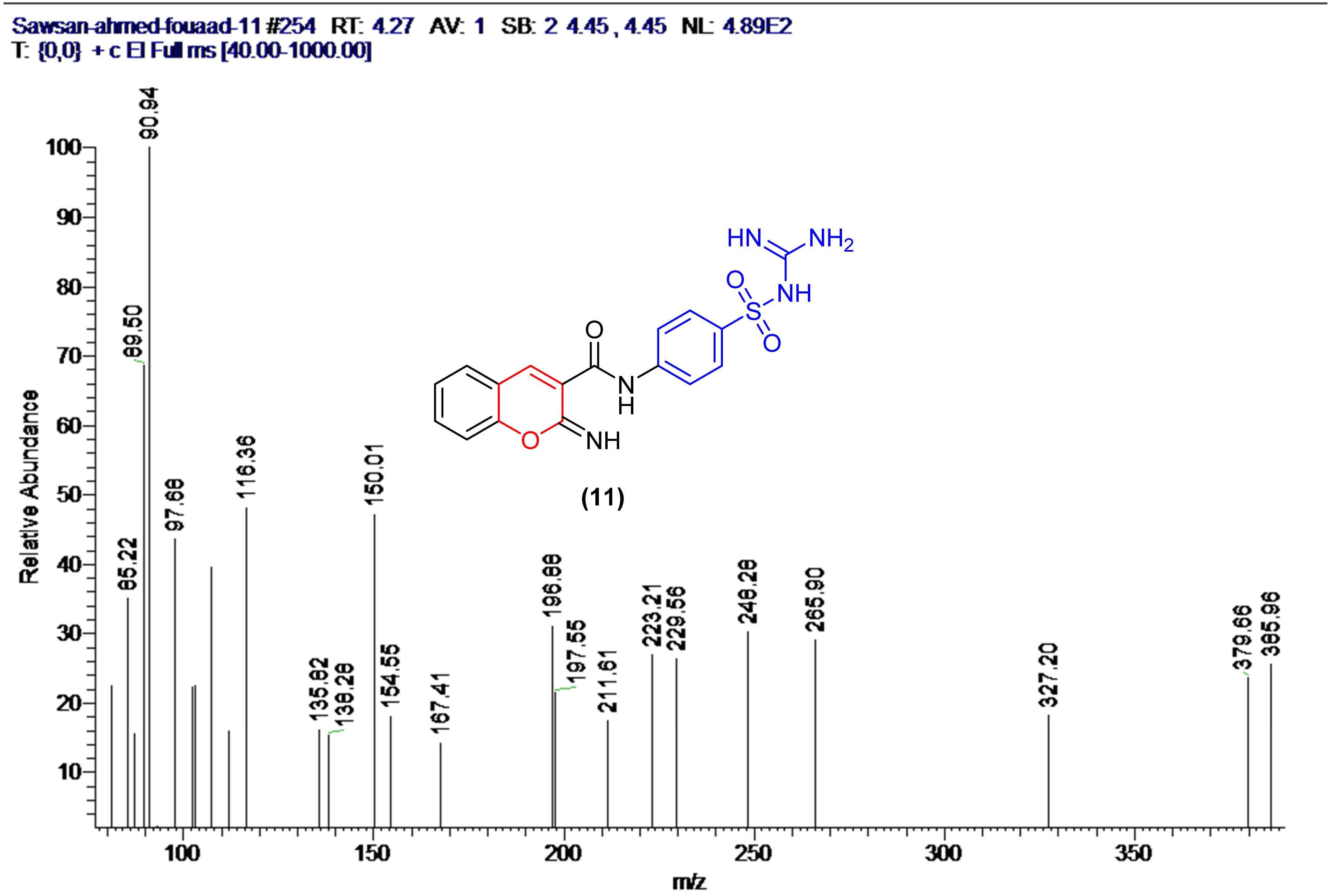

Sawsan-ahmed-fouaad-11 #254 RT: 4.27 AV: 1 SB: 2 4.45 , 4.45 NL: 4.89E2
T: {0,0} + c EI Full ms [40.00-1000.00]
Relative Abundance
m/z
90.94
89.50
85.22
97.68
116.36
150.01
135.82
138.28
154.55
167.41
196.88
197.55
211.61
223.21
229.56
248.28
265.90
327.20
379.66
385.96
(11)

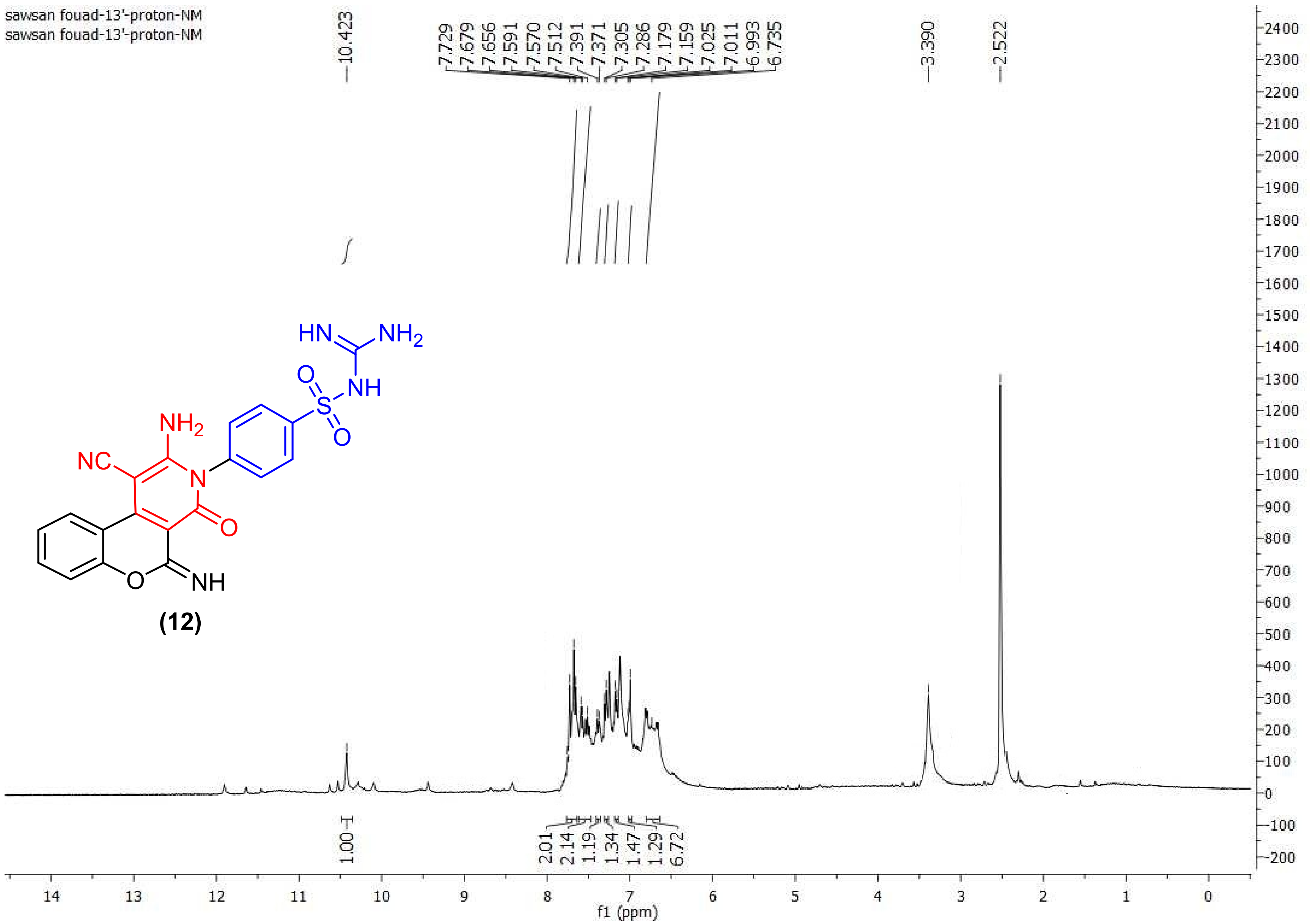
sawsan fouad-13'-proton-NM
sawsan fouad-13'-proton-NM
10.423
7.729 7.679 7.656 7.591 7.570 7.512 7.391 7.371 7.305 7.286 7.179 7.159 7.025 7.011 6.993 6.735
3.390
2.522
HN NH2 O S NH O NH2 NC N O O NH
(12)
1.00
2.01 2.14 1.19 1.34 1.47 1.29 6.72
14 13 12 11 10 9 8 7 6 5 4 3 2 1 0
f1 (ppm)
2400 2300 2200 2100 2000 1900 1800 1700 1600 1500 1400 1300 1200 1100 1000 900 800 700 600 500 400 300 200 100 0 -100 -200

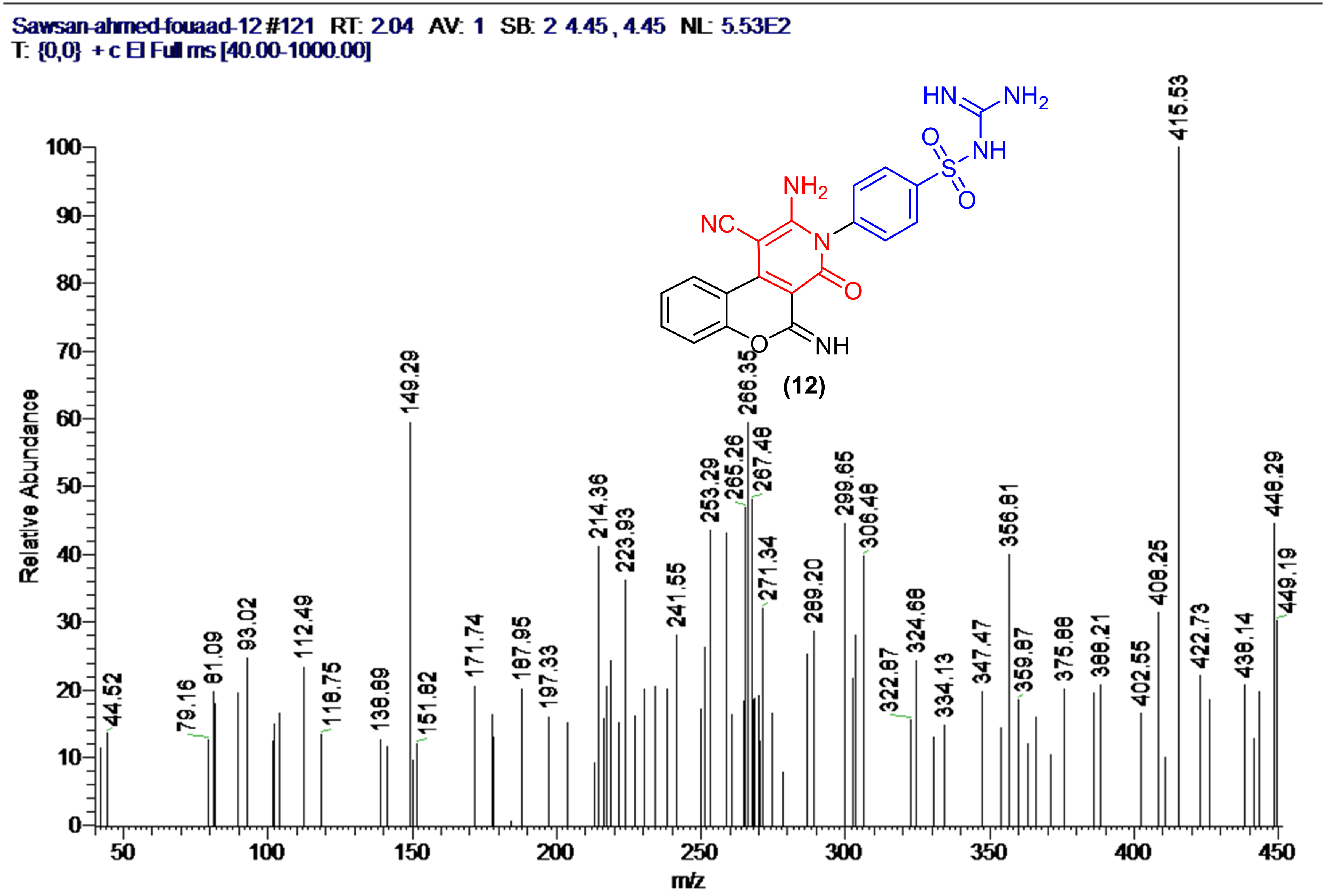

Sawsan-ahmed-fouaad-12 #121 RT: 2.04 AV: 1 SB: 2 4.45 , 4.45 NL: 5.53E2
T: {0,0} + c EI Full ms [40.00-1000.00]
(12)
Relative Abundance
m/z
415.53
149.29
266.35
265.26
267.48
253.29
299.65
446.29
214.36
306.48
356.81
223.93
449.19
271.34
408.25
289.20
241.55
93.02
112.49
324.68
422.73
438.14
347.47
375.88
388.21
81.09
359.87
118.75
79.16
44.52
138.89
151.82
171.74
187.95
197.33
322.87
334.13
402.55

## IR spectra of the newly designed compounds

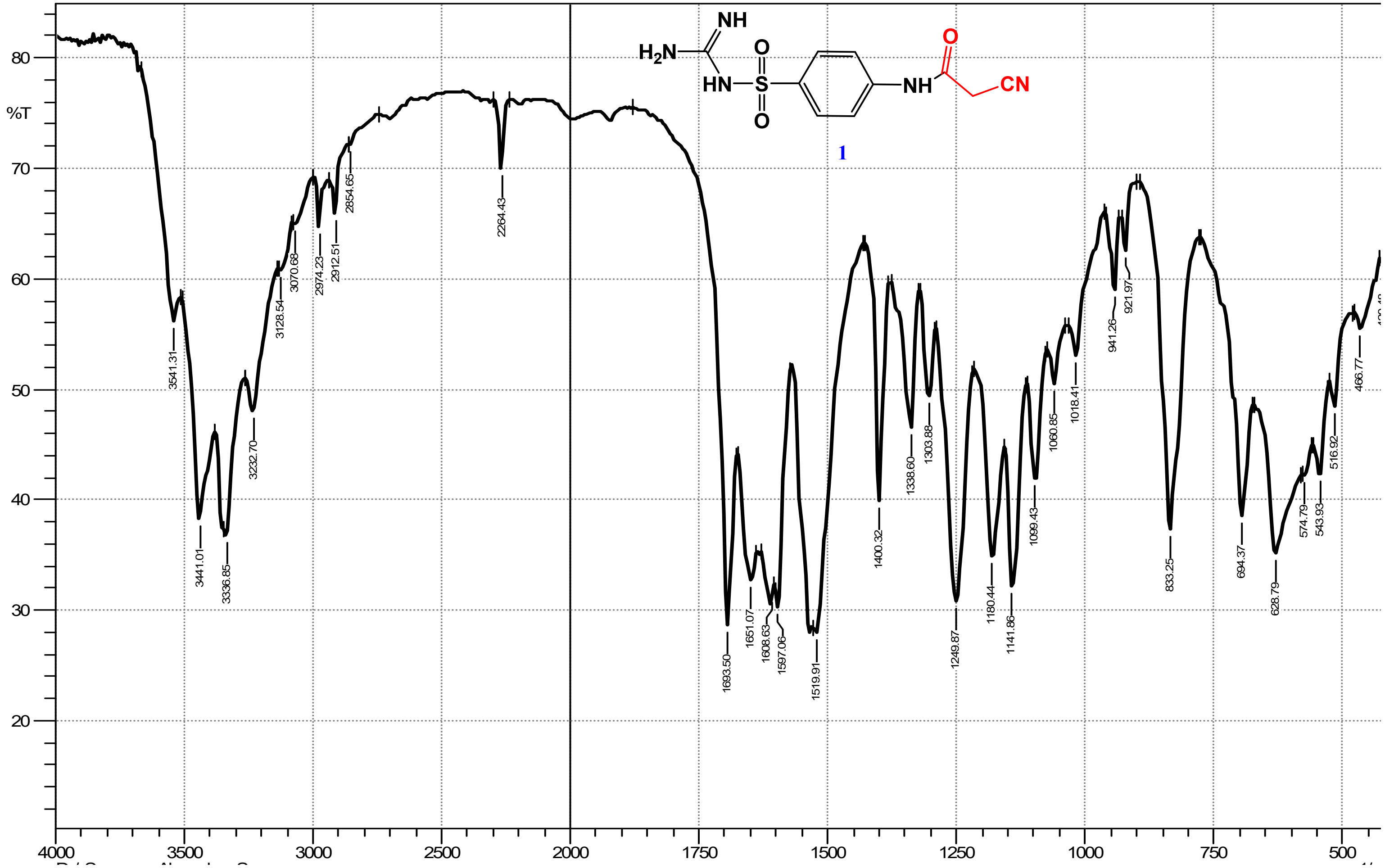


[1]

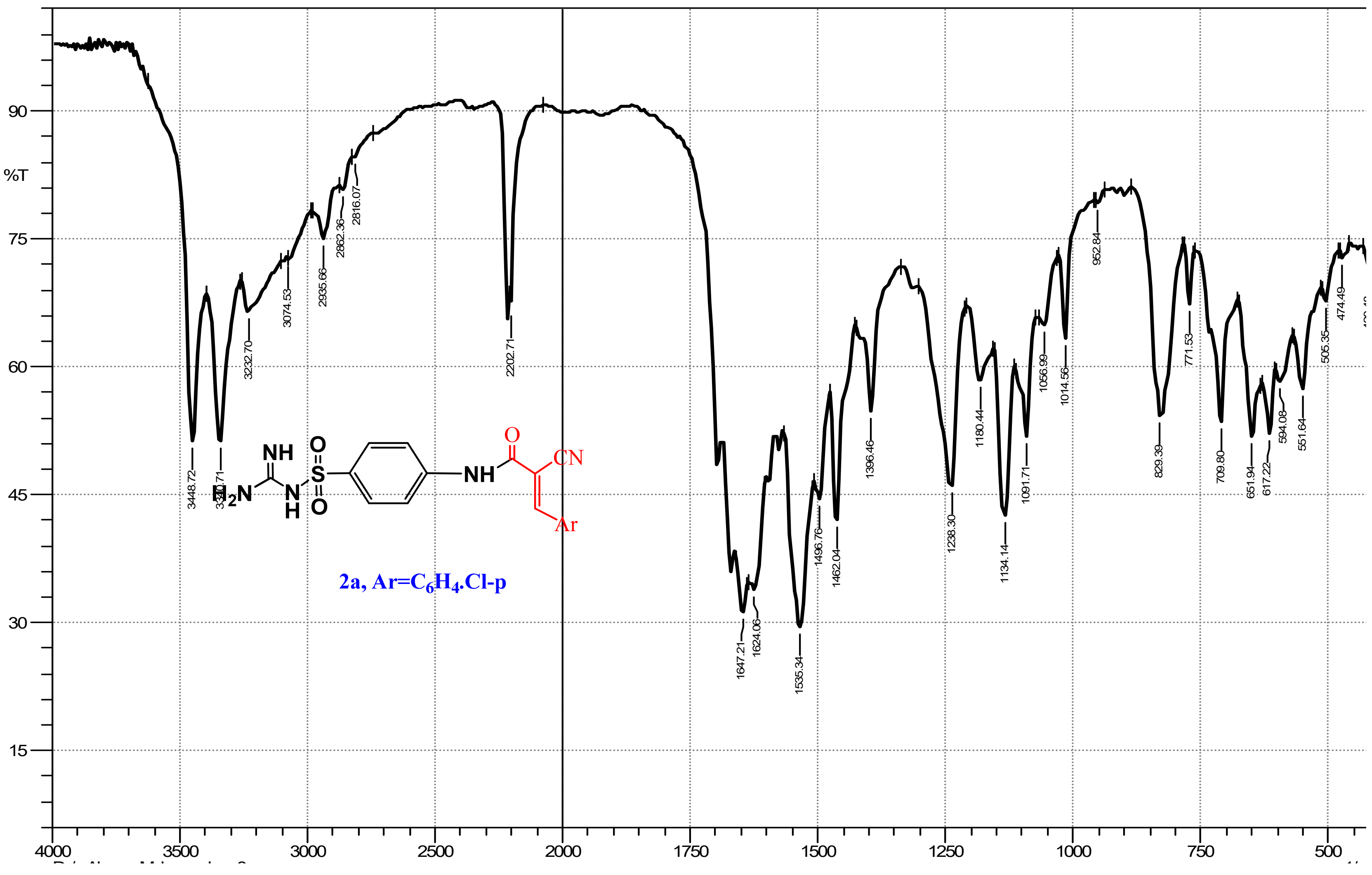
%T
90
75
60
45
30
15
4000
3500
3000
2500
2000
1750
1500
1250
1000
750
500
3448.72
3232.70
3074.53
2935.66
2862.36
2816.07
2202.71
1647.21
1624.06
1535.34
1496.76
1462.04
1396.46
1238.30
1180.44
1134.14
1091.71
1056.99
1014.56
952.84
829.39
771.53
709.80
651.94
617.22
594.08
551.64
505.35
474.49
NH
O
S
O
H2N
N
H
NH
O
CN
Ar
2a, Ar=C6H4.Cl-p

**[2a]**

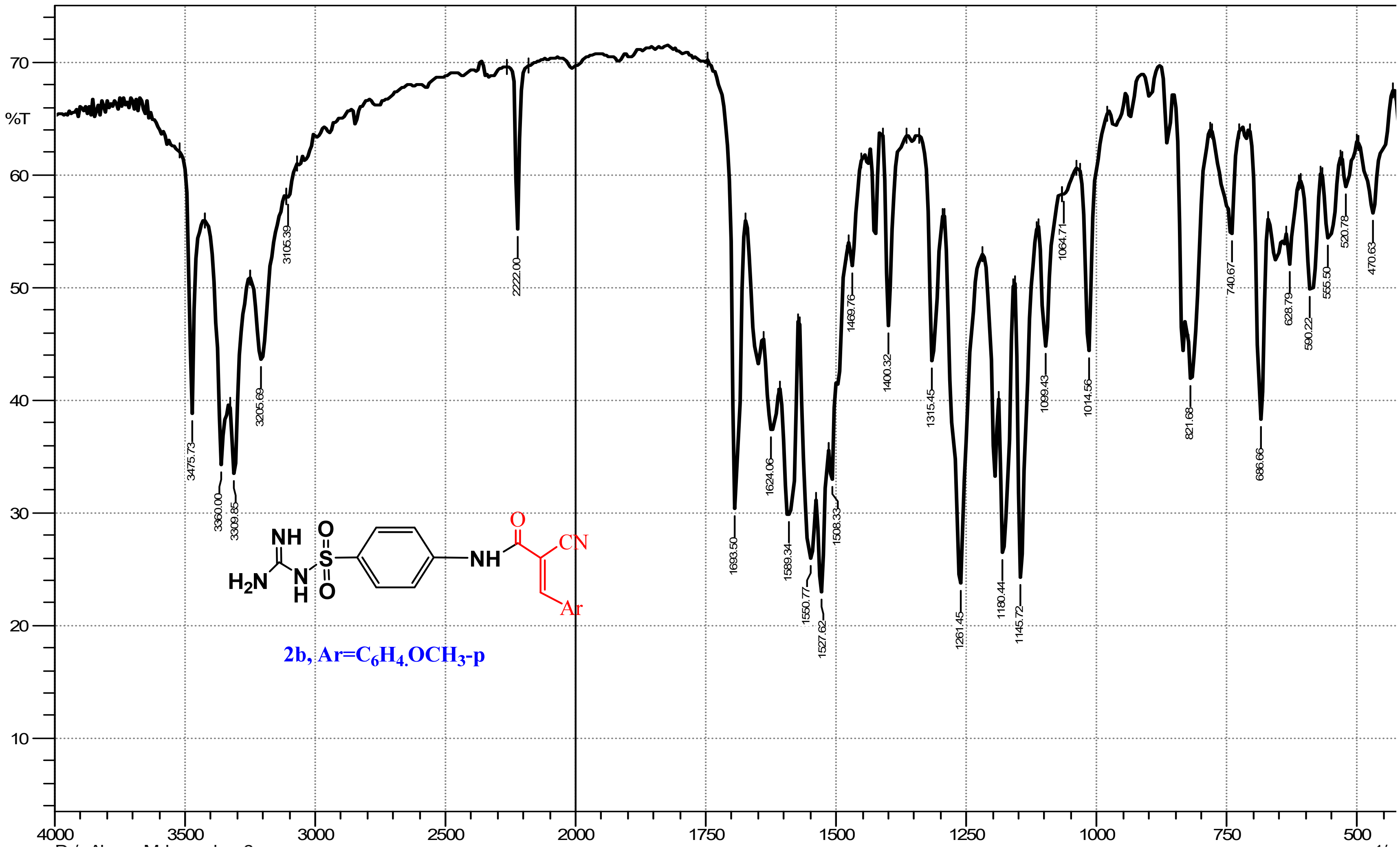
%T
70
60
50
40
30
20
10
4000
3500
3000
2500
2000
1750
1500
1250
1000
750
500
3475.73
3360.00
3309.85
3205.69
3105.39
2222.00
1693.50
1624.06
1589.34
1550.77
1527.62
1508.33
1469.76
1400.32
1315.45
1261.45
1180.44
1145.72
1099.43
1064.71
1014.56
821.68
740.67
686.66
628.79
590.22
555.50
520.78
470.63
NH
H2N
N
H
S
O
O
NH
O
CN
Ar
2b, Ar=C6H4.OCH3-p

**[2b]**

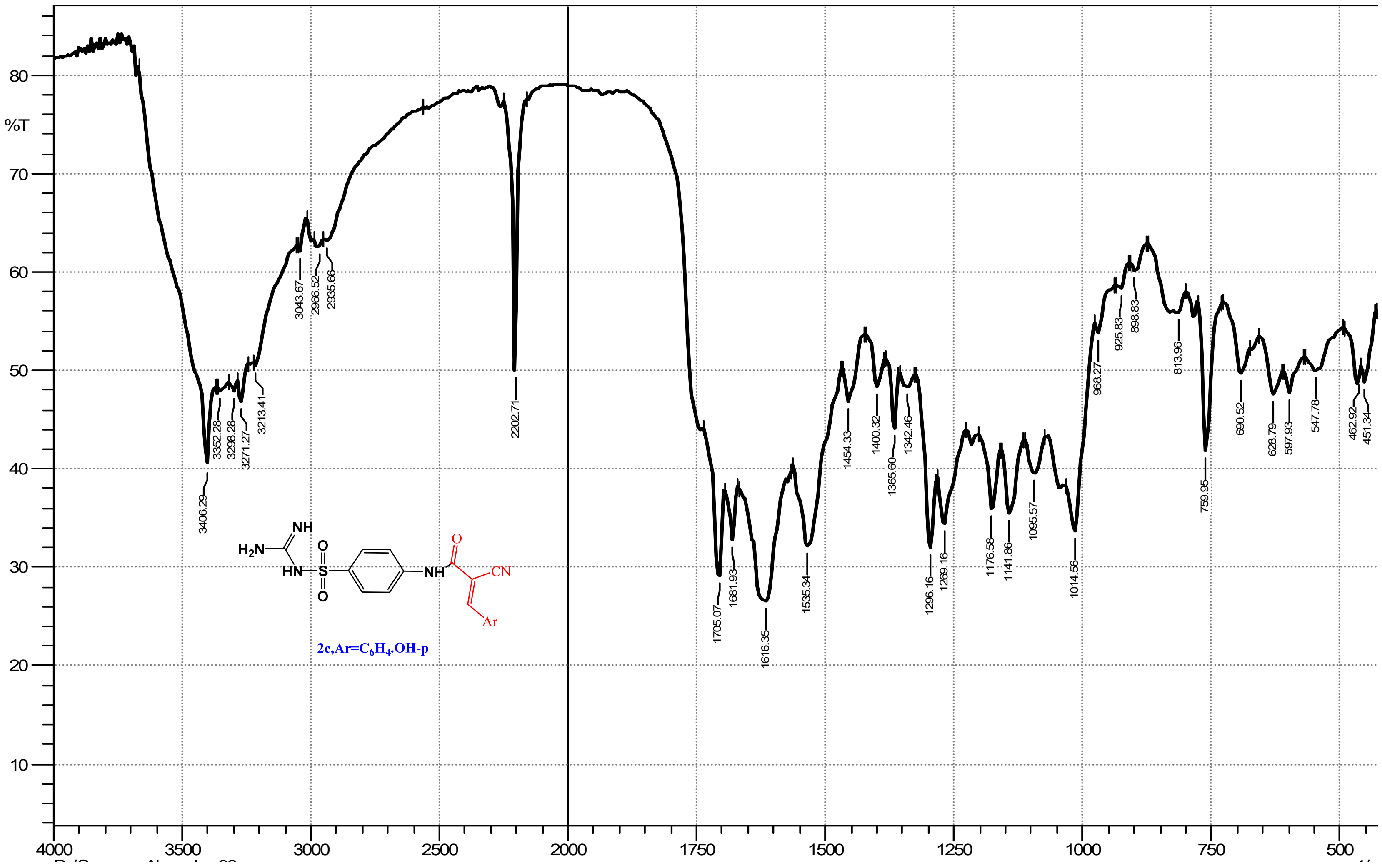
%T
80
70
60
50
40
30
20
10
4000
3500
3000
2500
2000
1750
1500
1250
1000
750
500
3406.29
3352.28
3298.28
3271.27
3213.41
3043.67
2966.52
2935.66
2202.71
1705.07
1681.93
1616.35
1535.34
1454.33
1400.32
1365.60
1342.46
1296.16
1269.16
1176.58
1141.86
1095.57
1014.56
968.27
925.83
898.83
813.96
759.95
690.52
628.79
597.93
547.78
462.92
451.34
NH
$H_2N$
HN
S
O
O
NH
O
CN
Ar
2c,Ar=$C_6H_4$.OH-p

**[2C]**

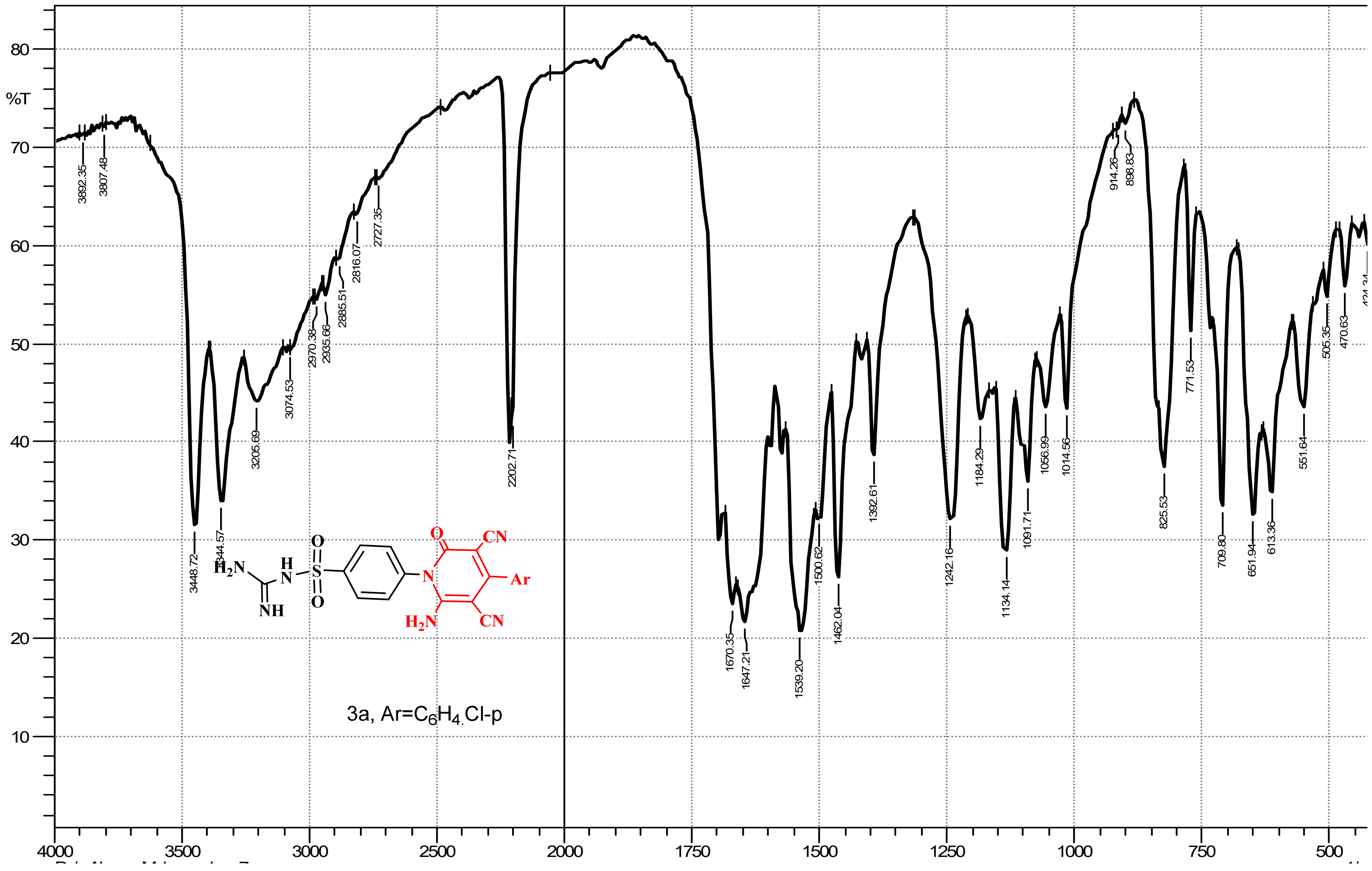
%T
3a, Ar=C6H4.Cl-p
3892.35
3807.48
3448.72
3205.69
3074.53
2970.38
2935.66
2885.51
2816.07
2727.35
2202.71
1670.35
1647.21
1539.20
1500.62
1462.04
1392.61
1242.16
1184.29
1134.14
1091.71
1056.99
1014.56
914.26
898.83
825.53
771.53
709.80
651.94
613.36
551.64
505.35
470.63

**[3a]**

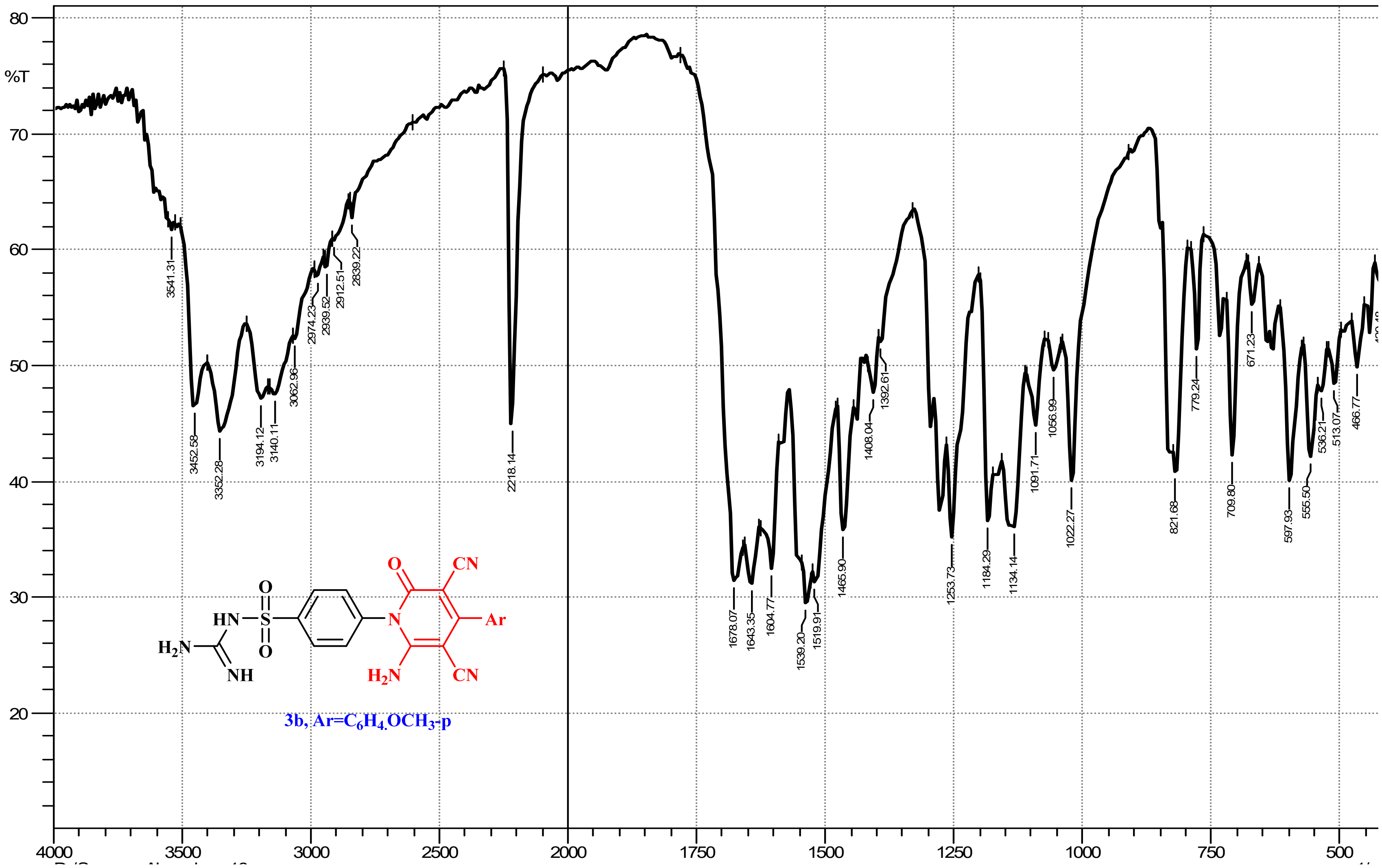
%T
80
70
60
50
40
30
20
4000
3500
3000
2500
2000
1750
1500
1250
1000
750
500
3541.31
3452.58
3352.28
3194.12
3140.11
3062.96
2974.23
2939.52
2912.51
2839.22
2218.14
1678.07
1643.35
1604.77
1539.20
1519.91
1465.90
1408.04
1392.61
1253.73
1184.29
1134.14
1091.71
1056.99
1022.27
821.68
779.24
709.80
671.23
597.93
555.50
536.21
513.07
466.77
O
CN
O
HN
S
O
N
Ar
H2N
NH
H2N
CN
3b, Ar=C6H4.OCH3-p

**[3b]**

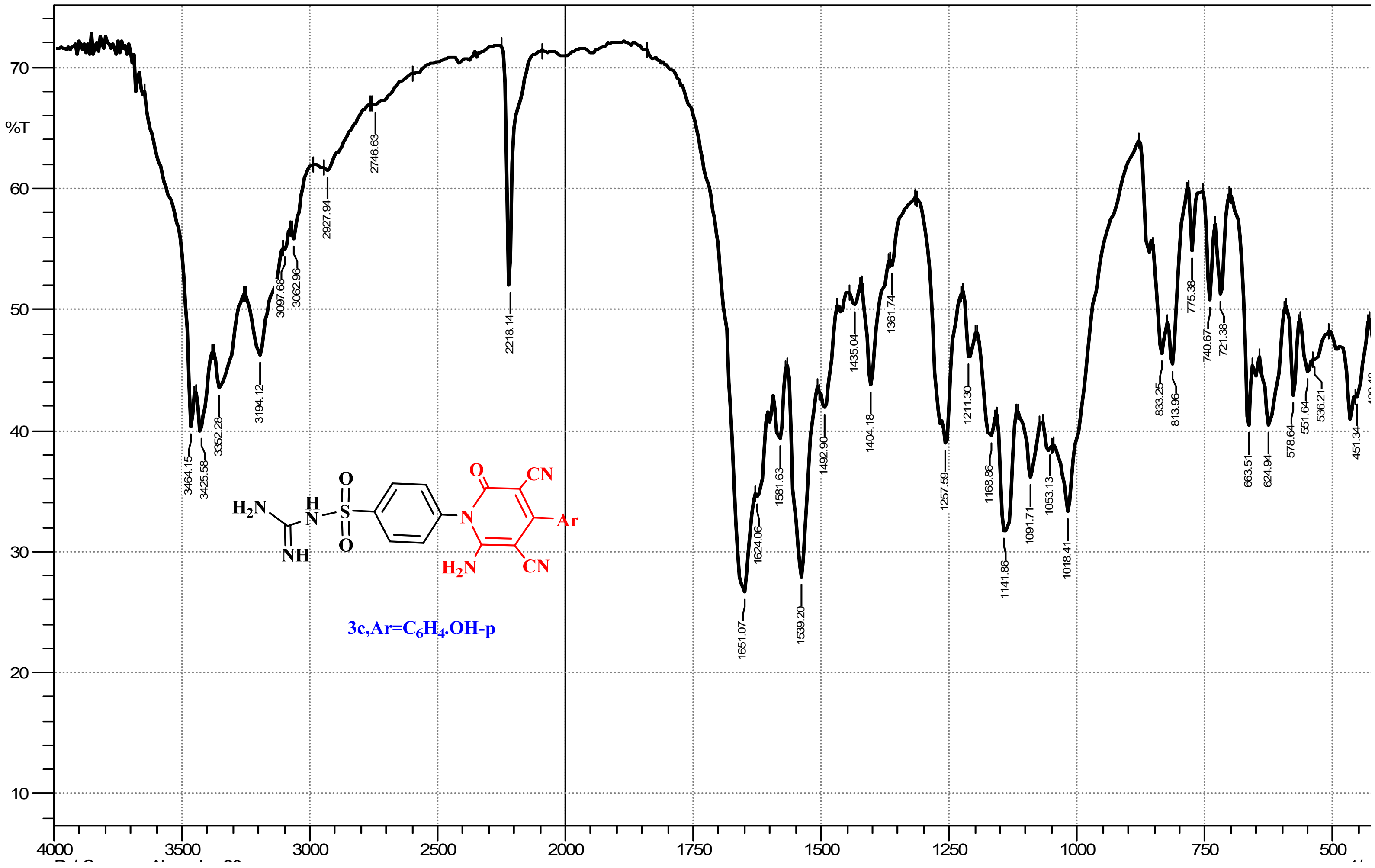
%T
70
60
50
40
30
20
10
4000
3500
3000
2500
2000
1750
1500
1250
1000
750
500
3464.15
3425.58
3352.28
3194.12
3097.68
3062.96
2927.94
2746.63
2218.14
1651.07
1624.06
1581.63
1539.20
1492.90
1435.04
1404.18
1361.74
1257.59
1211.30
1168.86
1141.86
1091.71
1053.13
1018.41
833.25
813.96
775.38
740.67
721.38
663.51
624.94
578.64
551.64
536.21
451.34
3c,Ar=C6H4.OH-p

**[3c]**

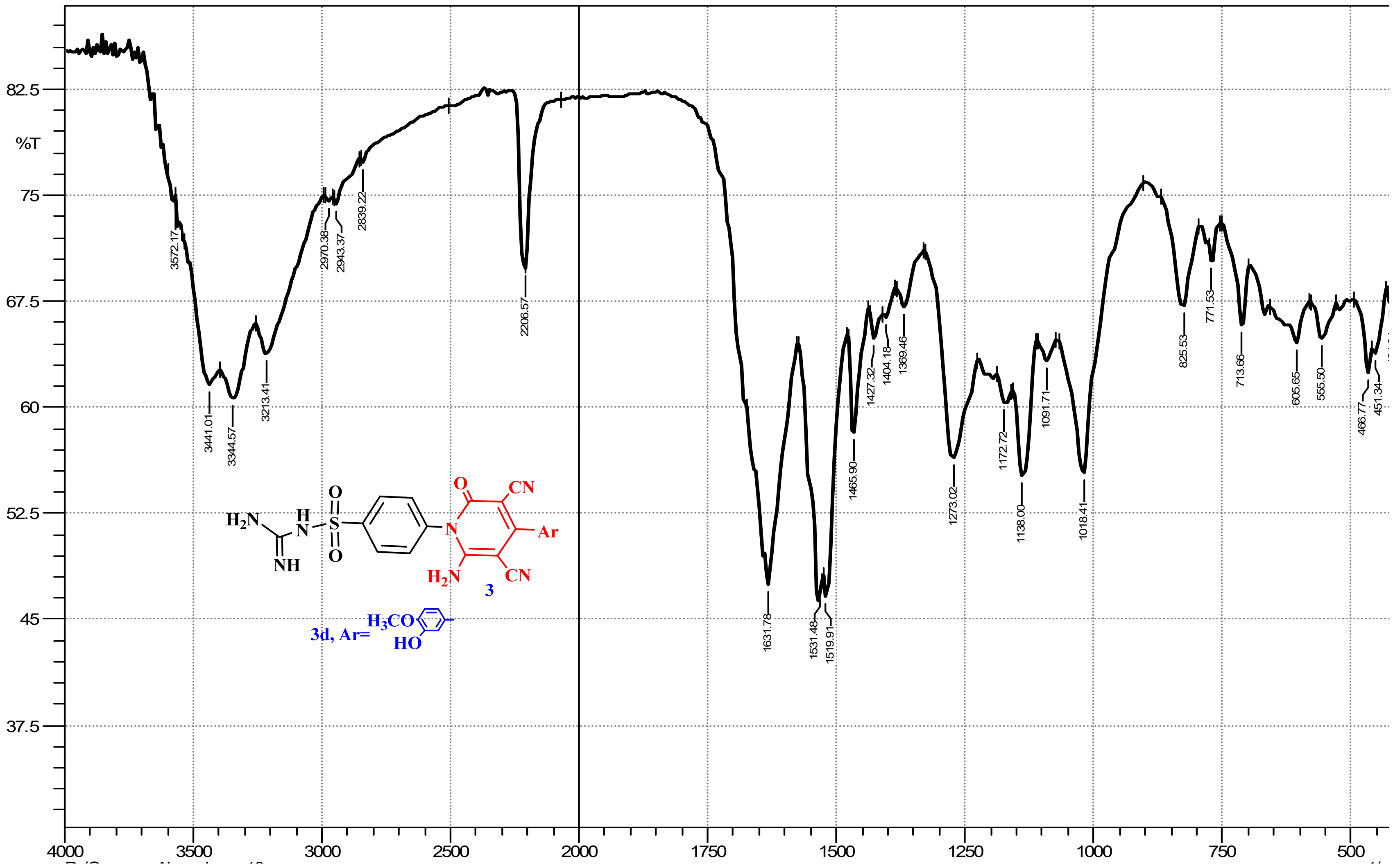

%T
82.5
75
67.5
60
52.5
45
37.5
4000
3500
3000
2500
2000
1750
1500
1250
1000
750
500
3572.17
3441.01
3344.57
3213.41
2970.38
2943.37
2839.22
2206.57
1631.78
1531.48
1519.91
1465.90
1427.32
1404.18
1369.46
1273.02
1172.72
1138.00
1091.71
1018.41
825.53
771.53
713.66
605.65
555.50
466.77
451.34
H2N
H
N
NH
O
S
O
N
O
CN
Ar
CN
H2N
3
3d, Ar=
H3CO
HO


**[3d]**

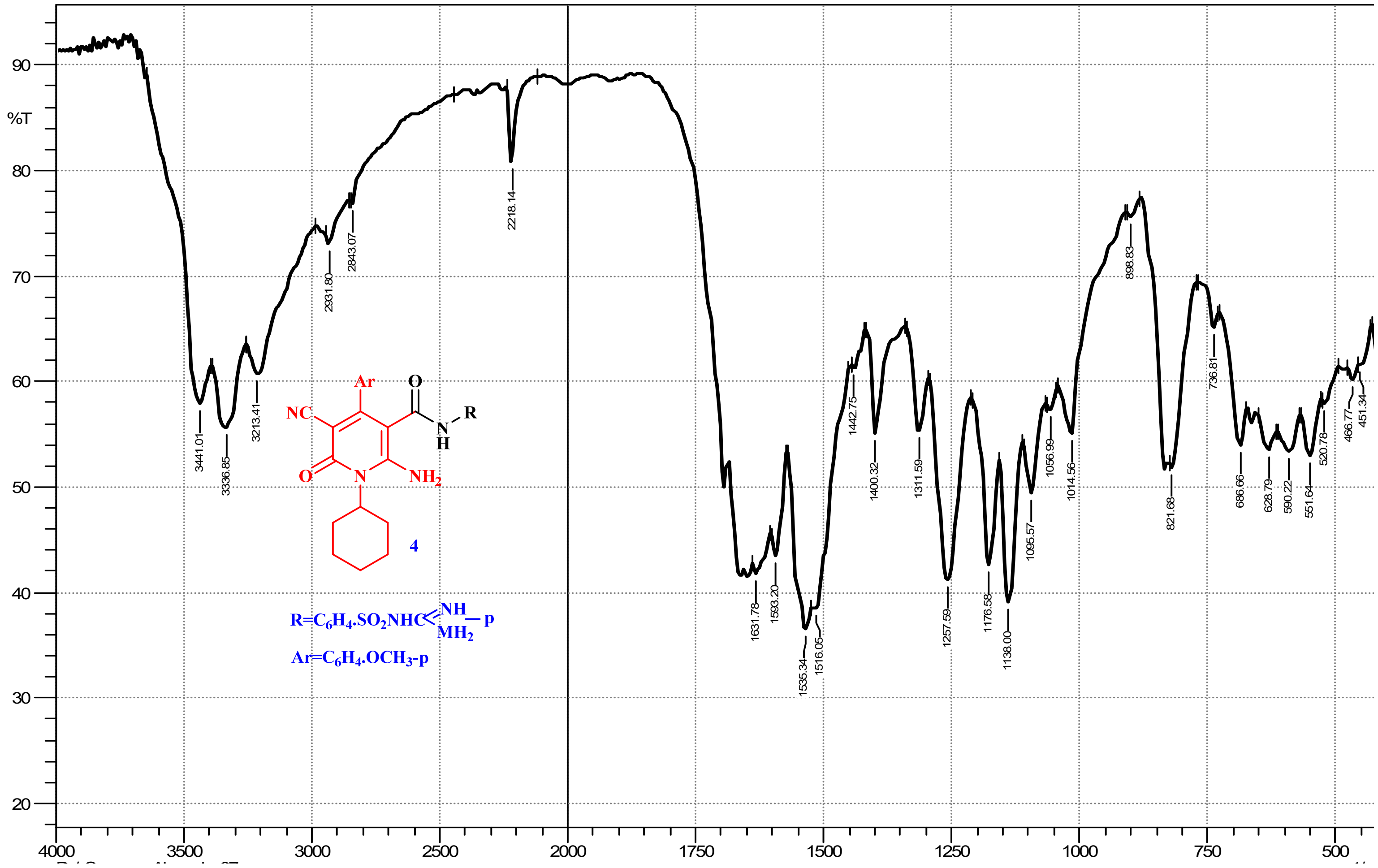
%T
90
80
70
60
50
40
30
20
4000
3500
3000
2500
2000
1750
1500
1250
1000
750
500
3441.01
3336.85
3213.41
2931.80
2843.07
2218.14
1631.78
1593.20
1535.34
1516.05
1442.75
1400.32
1311.59
1257.59
1176.58
1138.00
1095.57
1056.99
1014.56
898.83
821.68
736.81
686.66
628.79
590.22
551.64
520.78
466.77
451.34
Ar
O
NC
N
H
R
O
N
NH2
4
R=C6H4.SO2NHC NH — p MH2
Ar=C6H4.OCH3-p

**[4]**

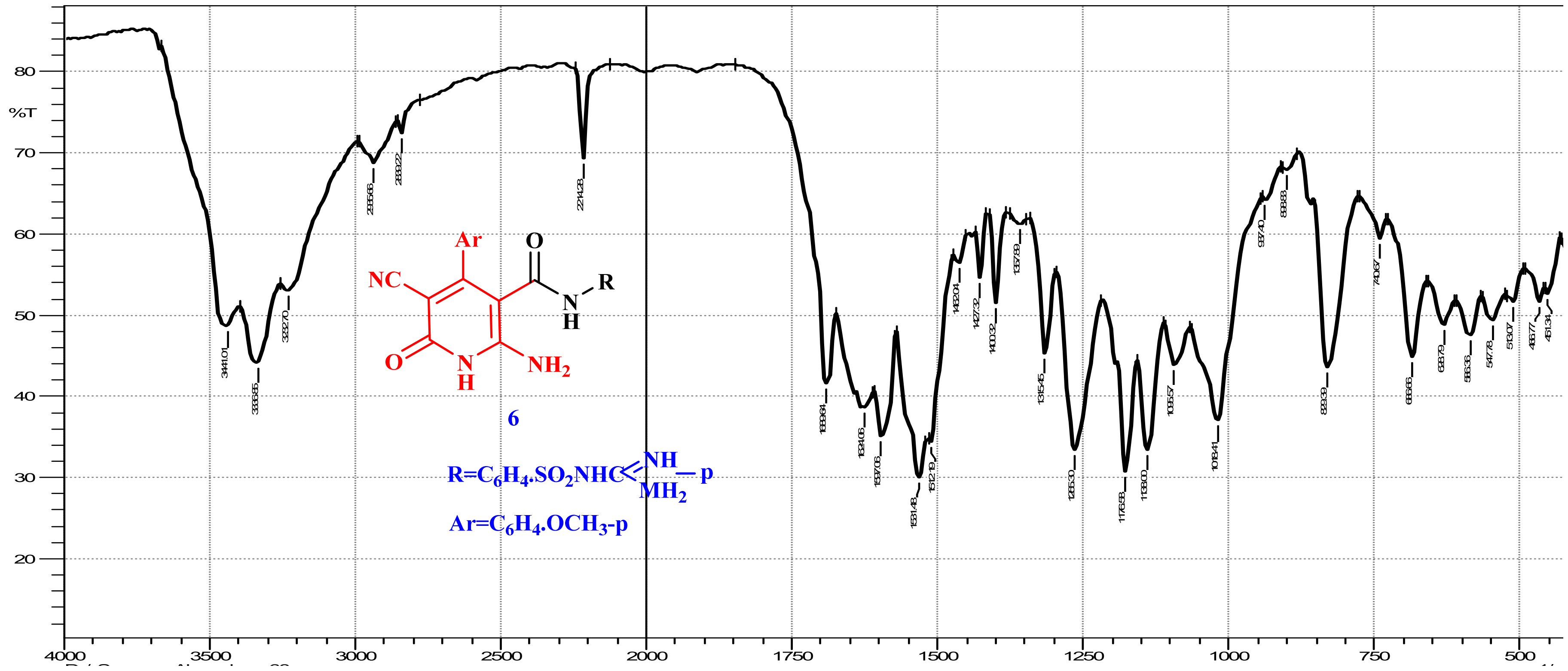


**[6]**

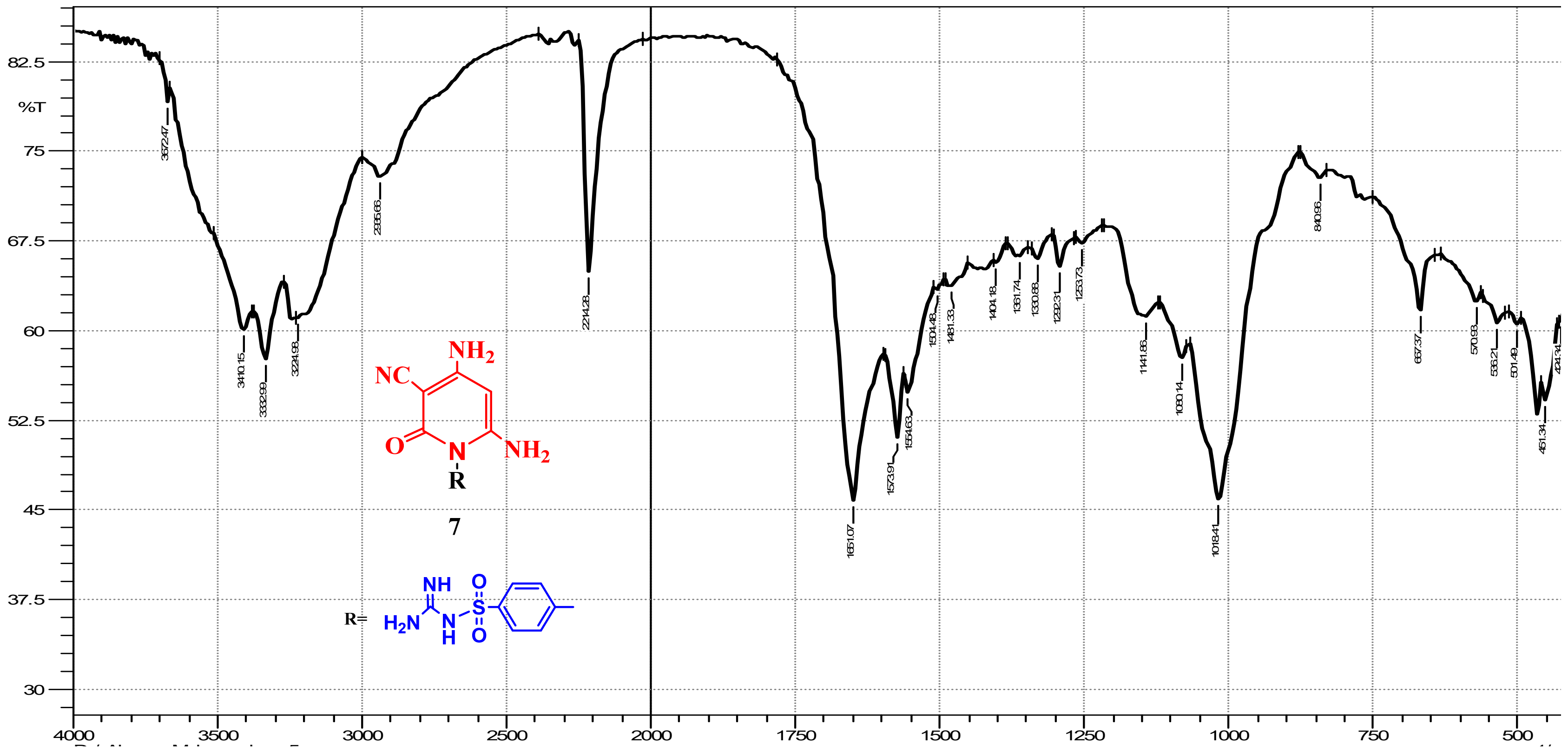

[7]

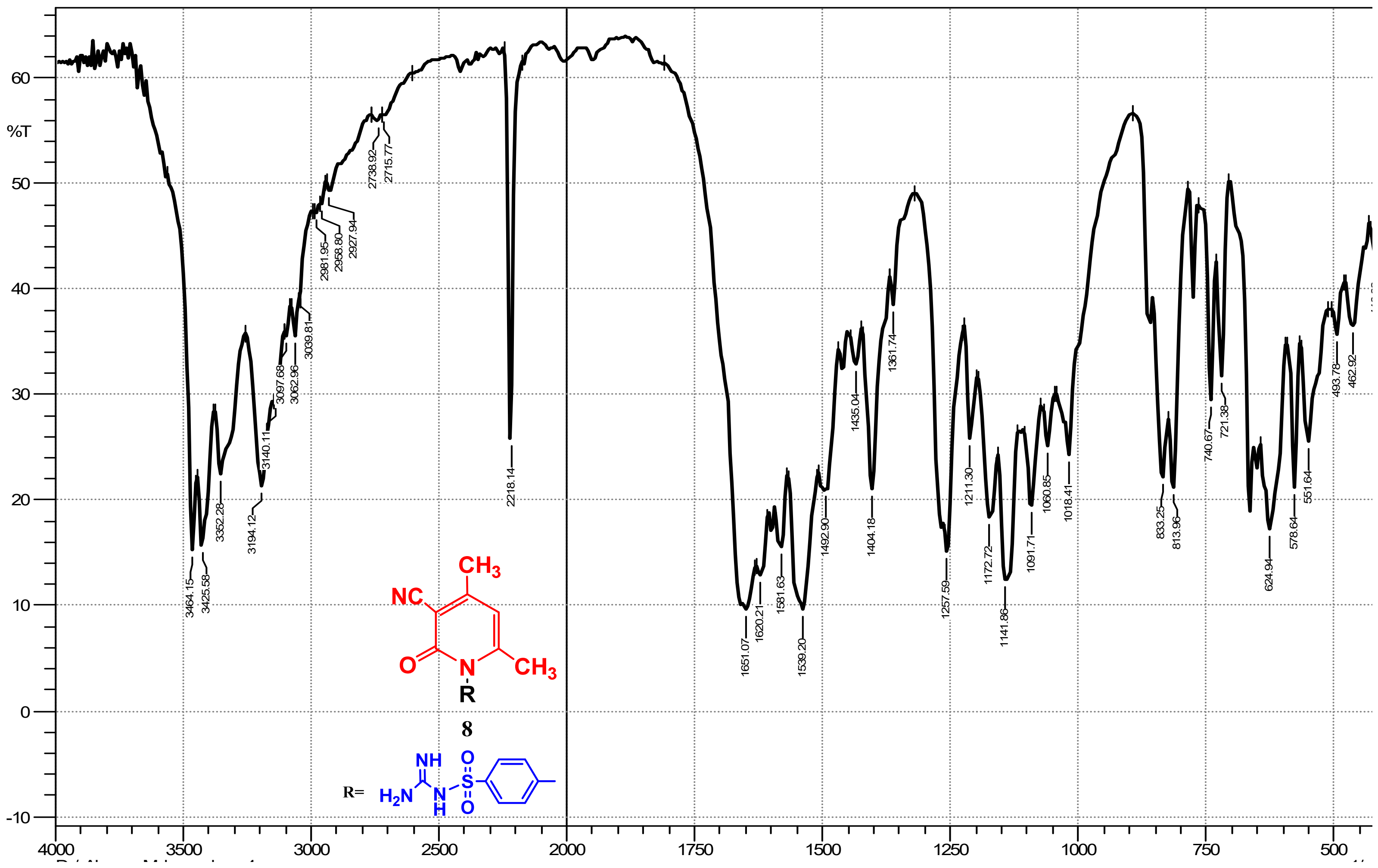
%T
60
50
40
30
20
10
0
-10
4000
3500
3000
2500
2000
1750
1500
1250
1000
750
500
3464.15
3425.58
3352.28
3194.12
3140.11
3097.68
3062.96
3039.81
2981.95
2958.80
2927.94
2738.92
2715.77
2218.14
1651.07
1620.21
1581.63
1539.20
1492.90
1435.04
1404.18
1361.74
1257.59
1211.30
1172.72
1141.86
1091.71
1060.85
1018.41
833.25
813.96
740.67
721.38
624.94
578.64
551.64
493.78
462.92
CH3
NC
O
N
CH3
R
8
R=
NH
O
H2N
N
H
S
O

**[8]**

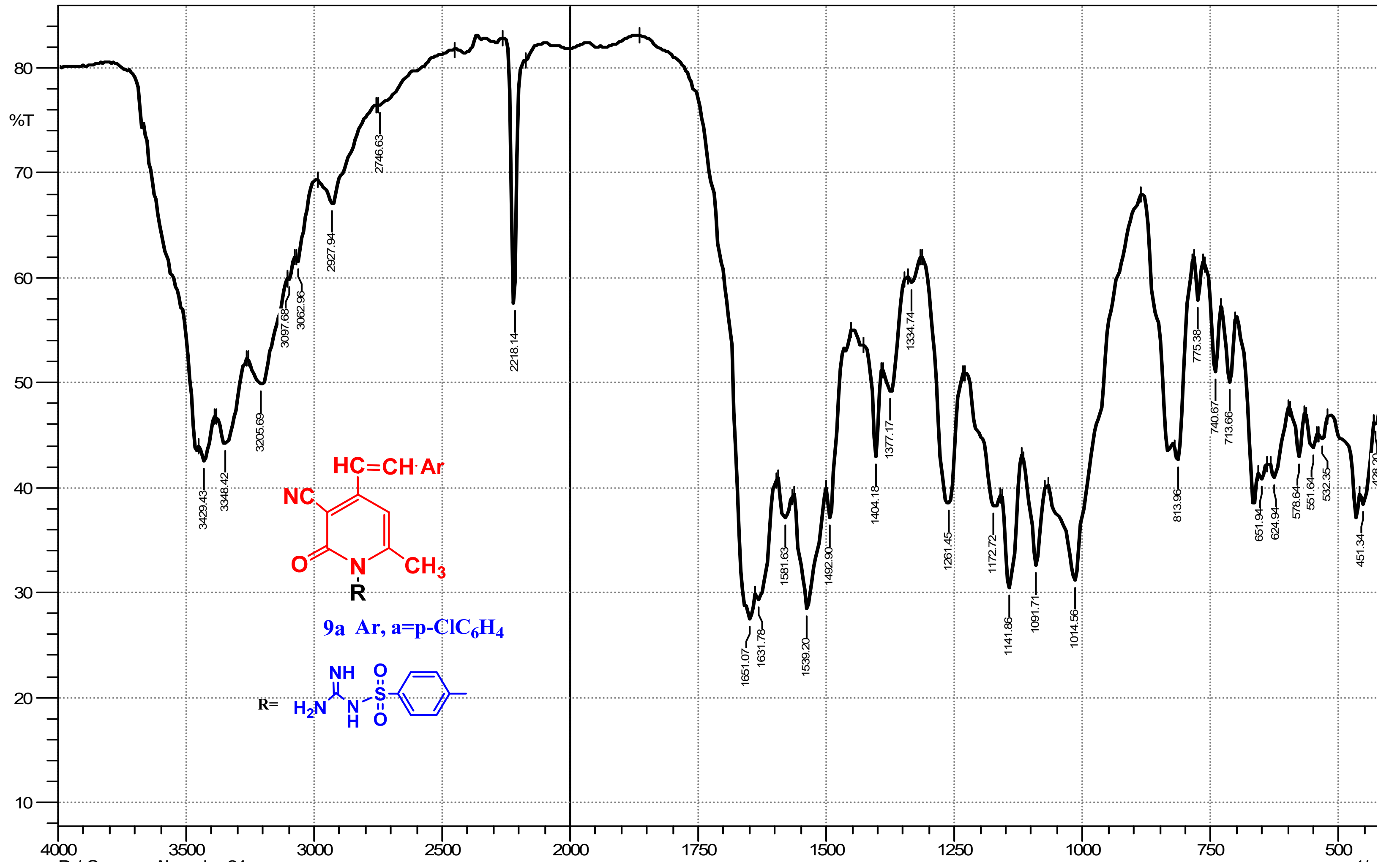


**[9a]**

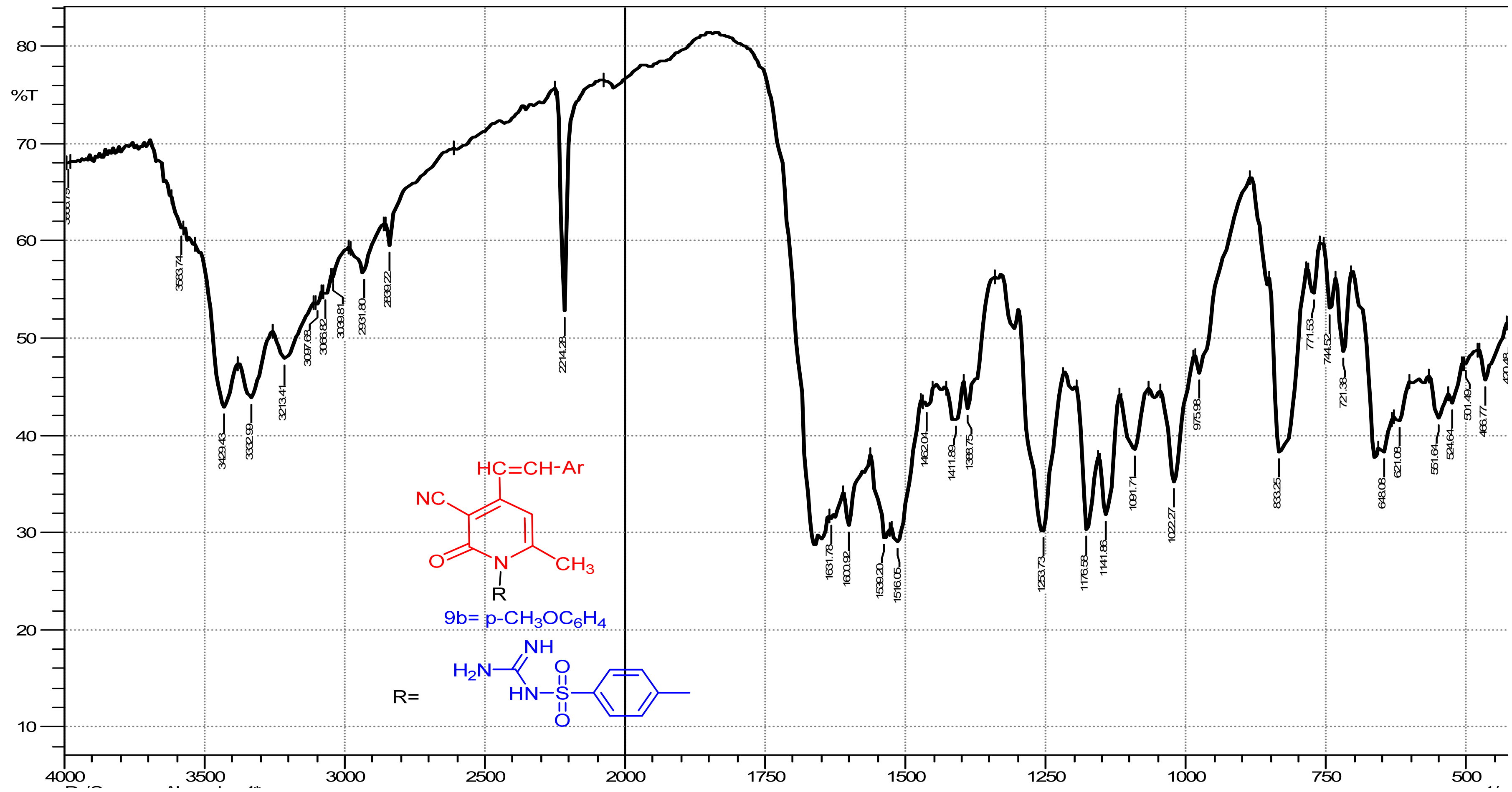

**[9b]**

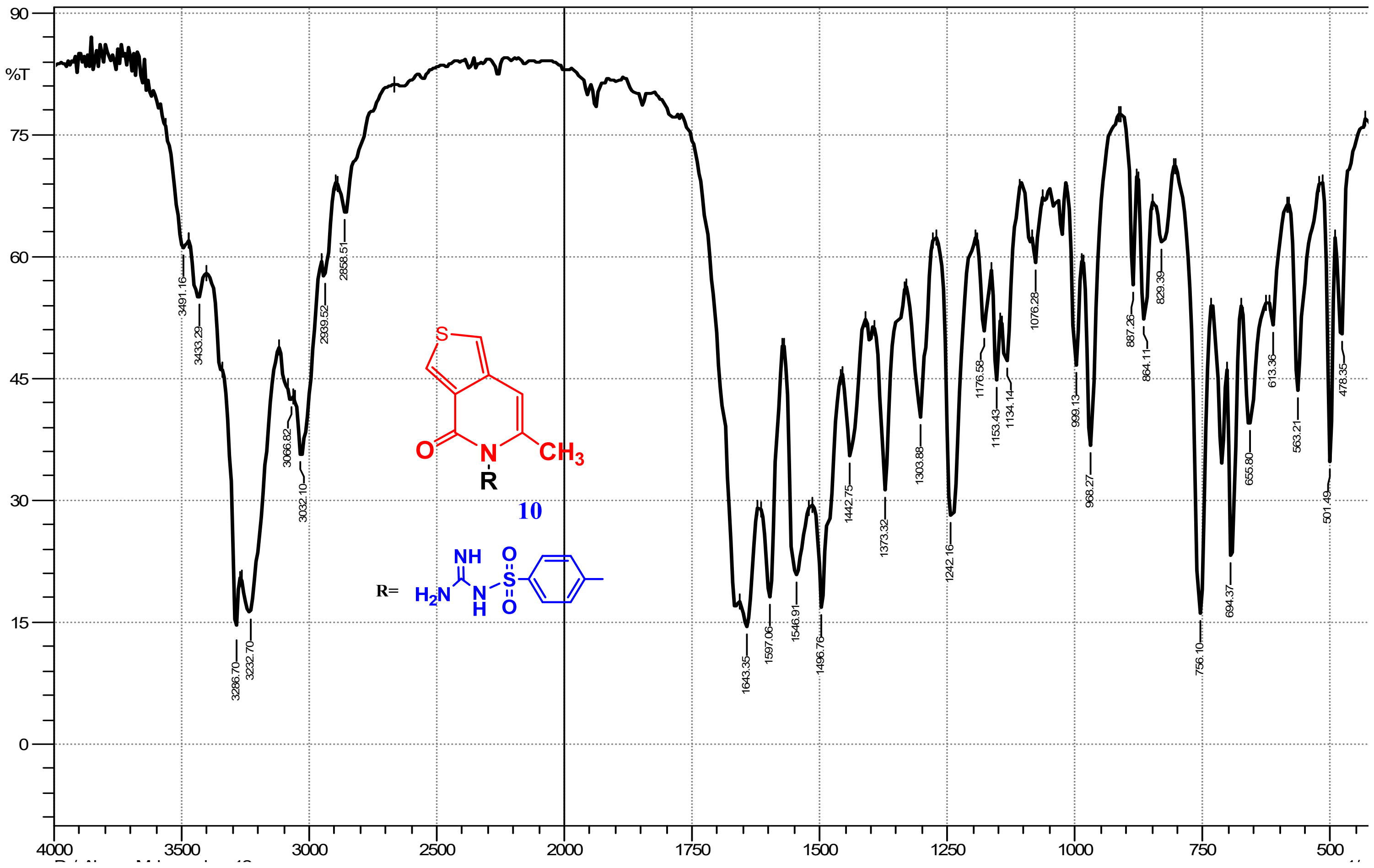
90
75
60
45
30
15
0
%T
4000
3500
3000
2500
2000
1750
1500
1250
1000
750
500
3491.16
3433.29
3286.70
3232.70
3066.82
3032.10
2939.52
2858.51
1643.35
1597.06
1546.91
1496.76
1442.75
1373.32
1303.88
1242.16
1176.58
1153.43
1134.14
1076.28
999.13
968.27
887.26
864.11
829.39
756.10
694.37
655.80
613.36
563.21
501.49
478.35
S
O
N
R
CH3
10
R=
NH
H2N
N
H
S
O
O

**[10]**

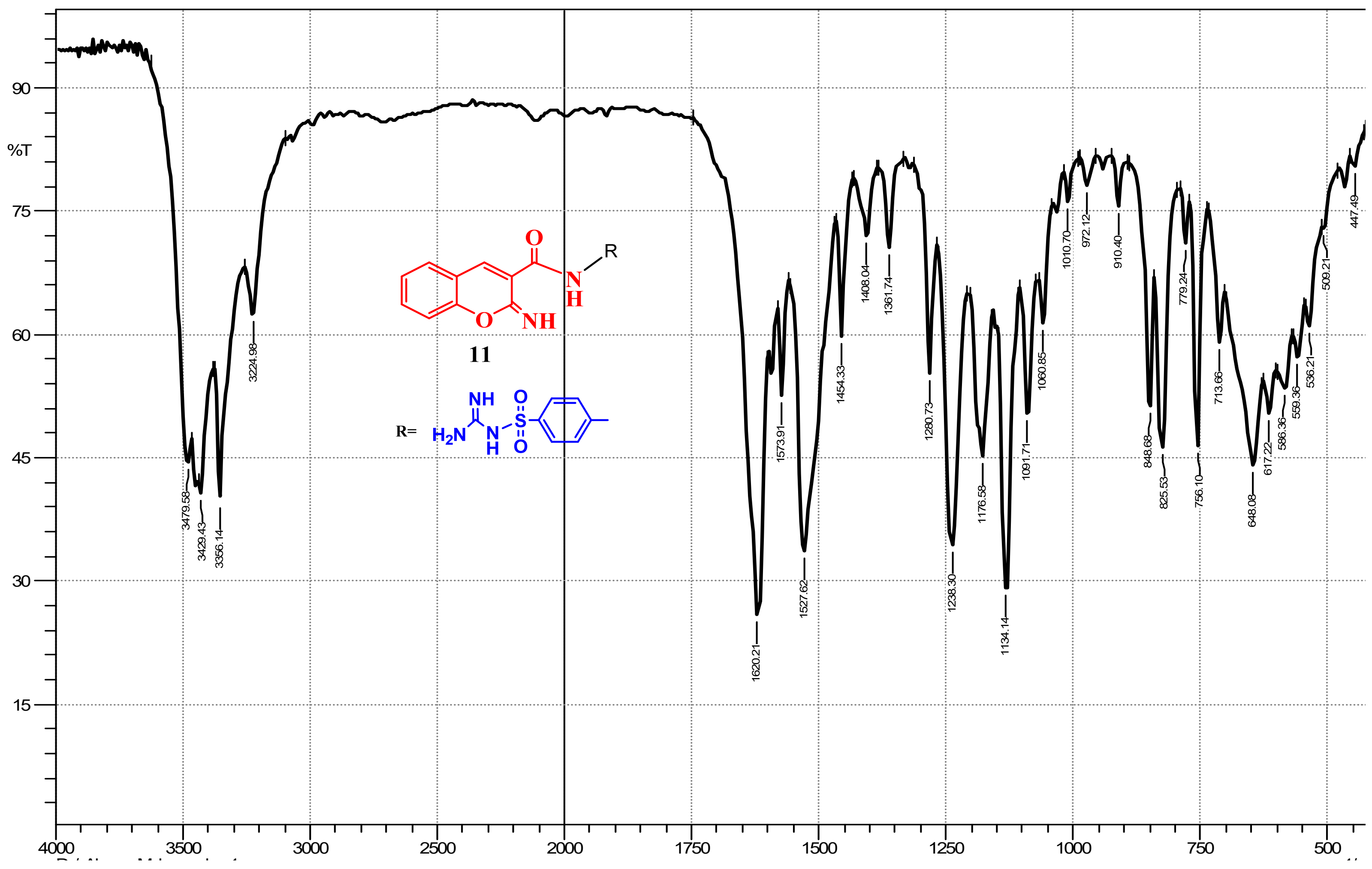
%T
90
75
60
45
30
15
4000
3500
3000
2500
2000
1750
1500
1250
1000
750
500
3479.58
3429.43
3356.14
3224.98
1620.21
1573.91
1527.62
1454.33
1408.04
1361.74
1280.73
1238.30
1176.58
1134.14
1091.71
1060.85
1010.70
972.12
910.40
848.68
825.53
779.24
756.10
713.66
648.08
617.22
586.36
559.36
536.21
509.21
447.49
O
N
H
R
O
NH
11
R=
NH
H2N
N
H
O
S
O

**[11]**

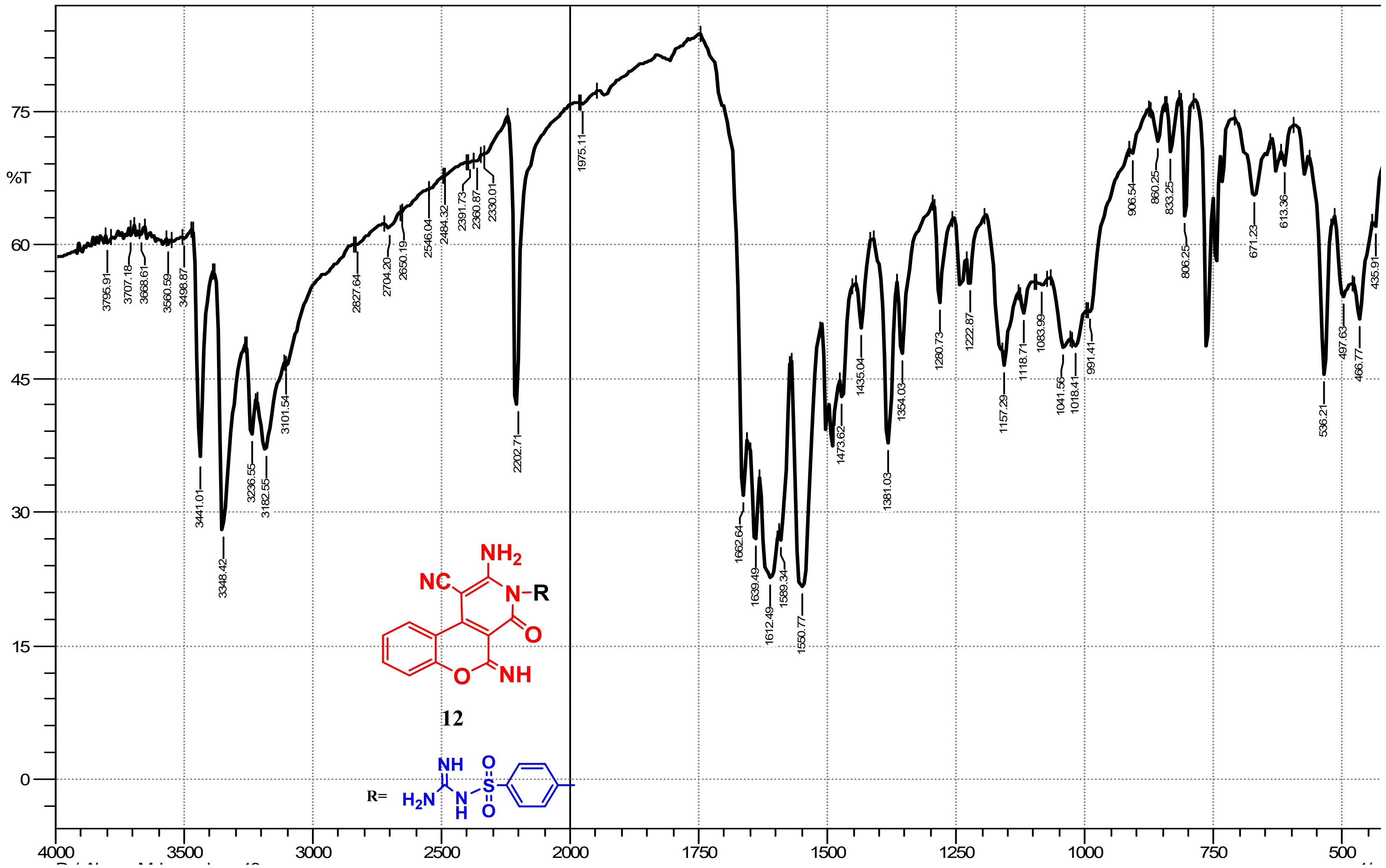


**[12]**

List of Chemical that used in this study for synthesis.

| Compound name | Company |
|---|---|

| | |
|---|---|
| Sulfaguanidine | AL Nasr Pharmaceutical Chemicals Company |
| Ethyl acetoacetate | Sigma-Aldrich |
| 4-chloro benzaldehyde | Merck |
| 4-methoxy benzaldehyde | Merck |
| 4-hydroxy benzaldehyde | Merck |
| 3-hydroxy-4-methoxy benzaldehyde | Merck |
| Cyanoacetamide | AL Nasr Pharmaceutical Chemicals Company |
| DMF | Sigma-Aldrich |
| Ethanol (HPLC) | Merck |
| Piperidine | Loba Chemical |
| Acetyl acetone | AL Nasr Pharmaceutical Chemicals Company |
| Amm. Acetate | AL Nasr Pharmaceutical Chemicals Company |
| Malononitrile | Loba Chemical |
| Salicylaldehyde | AL Nasr Pharmaceutical Chemicals Company |
| Sulphur powder | AL Nasr Pharmaceutical Chemicals Company |

**Biological Activity**

1.1.1. Determination of Inhibition Zones (mm)

The preliminary antimicrobial activity of the newly modified sulfaguanidine derivatives **2a-d**, **3a-d**, **4**, **6**, **7**, **8**, **9a-b**, **10**, **11** and **12** was evaluated against eight microbial pathogen that were divided as three Gram-positive stains, besides three Gram-negative as (*B. subtilis* ATCC 6633, *S. aureus* ATCC 29213 and *E. faecalis* ATCC 29212), (*P. aeruginosa* ATCC 27853, *E. coli* ATCC 25922 and *S. typhi* ATCC 6539), as well as two fungal strains *F. oxysporum* (RCMB 008002) and *C. albicans* (ATCC 10231). The antimicrobial activity performed at the bacteriology laboratory, Botany and Microbiology Department, Faculty of Science, Al-Azhar University, Cairo, Egypt. The antimicrobial potential of the newly synthesized organic compounds was investigated towards the test microorganisms and expressed as the diameter of the inhibition zones according to the agar plate diffusion method, where both tetracycline and amphotericin B were used as the positive controls. Briefly, 100 µL of the test bacteria/fungi were grown in 10 mL of fresh media until they reached a count of approximately 108 cells/mL for bacteria or 105 cells/mL for fungi. One mL of each sample (at 0.5 mg/mL) was added to each well (10 mm diameter holes cut in the agar gel). The plates were incubated for 24 h at 37 °C (for bacteria and yeast) and for 72 h at 27 °C (for filamentous fungi), each test was repeated three times. After incubation, the microorganism's growth was observed. Tetracycline was used as standard antibacterial drugs while amphotericin B was used as a standard antifungal drug. The resulting inhibition zone diameters were measured in millimeters and used as a criterion for the antimicrobial activity. Solvent controls (DMSO) were included in every experiment as the negative controls. DMSO was used for dissolving the tested compounds and showed no inhibition zones, confirming that it does not influence the growth of the tested microorganisms.

1.1.2. Determination the Minimal Inhibition Concentration (MIC)

A conventional technique termed paper disk diffusion was used to investigate the MIC of the active compounds through employing a 12.7 mm diameter filter paper (Whatman, Germany). Bacteria were grown in a media of nutrient agar, while fungi and yeasts were grown in a media of Sabouraud´s agar. The synthesized compounds were dissolved and loaded on paper disks with different concentrations. Loading the drying disks over the agar plates' surface inoculated with the selected microorganisms was carried out, and then growth inhibition was tested when incubated (at 37 °C for a day) for the bacterial strains and yeasts and fungi (at 27 °C for three days); also, the MIC was confirmed by using the broth micro-dilution procedure described in the Clinical and Laboratory Standards Institute (CLSI) guidelines, where repeating of every experiment for three-times was performed for reproducibility.

#### 1.1.3. Evaluation of the Minimum Bactericidal Concentration (MBC)

An MBC assay was conducted using the broth microdilution assay described in the above section. The MBC was determined by plating 10 μL of culture volume from the MIC assay onto TSB agar plate and colony formation was examined after 24 h at 37 °C. The MBC is defined as the lowest compound concentration resulting in a ≧ 3-log reduction in the number of colony-forming units (CFU).

#### 1.1.4. Multidrug-Resistant Bacteria (MDRB)

To evaluate the antibacterial activity of most potent compounds toward the multidrug-resistant Gram-positive strains (*Staphylococcus aureus* ATCC 43300 and *Staphylococcus aureus* ATCC 33591) and multidrug-resistant Gram-negative strains (*Escherichia coli ATCC BAA*-196 and *Pseudomonas aeruginosa* ATCC BAA-2111). Both of the inhibition zones and MIC were determined by the same methods as described previously. The MBC assay against MDRB was carried out according to the previously reported method. Norfloxacin was chosen to be the control antibiotic.

#### 1.1.5. Immunomodulatory Activity

In vitro intracellular killing activities

*Neutrophil isolation:* From three healthy volunteers (5 mL blood from each one), a sample of peripheral blood (15 mL) was taken and collected in preservative-free heparin followed by adding 4.5% dextran B in saline (2 mL). The mixed solution was shaken and kept at 37 °C for 1 h to form the erythrocytes sediment. Isolation of the neutrophils through centrifugation was performed. Hypotonic lysis was used to remove the residual erythrocytes followed by washing the neutrophils via Hank’s balanced salt solution (HBSS, Sigma-Aldrich (USA), followed by suspending the washed neutrophils in the HBSS with a concentration of $25 \times 10^6$ cells/mL to assess the killing efficiency toward the strains. Trypan blue exclusion was used to assess the neutrophils' viability.

*Studying the activity of the intracellular killing:* The activity of the intracellular killing was studied through a nitro blue tetrazolium (NBT) reduction through the developed Baehner and Nathan technique. In HBSS, the isolated neutrophils and flavonoids were incubated at 37 °C (for half an hour) followed by extracting the blue formazon (reduced dye) through pyridine and the spectrophotometric measurements were carried out at 515 nm. A negative control sample (which includes all the reagents without the neutrophil suspen-

sion) was used for comparative studies. Absorbance variation between the negative control and the cell cultures which actively phagocyte latex particles express the index of the neutrophils' intracellular killing activity. A sample's activity can be calculated by dividing the active compound's percentage ratio in the latex to the positively charged control latex.

*In vivo immunomodulatory study:* The immunomodulation activity of three synthetic compounds were evaluated in vivo. Male laboratory-bred Swiss albino mice (6–8 weeks old) ($n$ = 84), weighing 20 ± 2 g, were used. They were randomly divided into five groups, three groups receiving compounds **2d**, **8** and **11** besides the positive control, and normal groups receiving vitamin C and normal diet. Immunomodulatory potential was determined by immune organ indices, immune cell population levels using the immune organ weight and index, and flow cytometry.

*Relative immune organs weight:* After the period of treating, mice were sacrificed, then both the spleen and thymus weight and indices were measured and calculated by divided the weight of spleen or thymus (mg) to body weight (g).

*Immune cells population:* Male laboratory-bred Swiss albino mice (6–8 weeks old) ($n$ = 84), weighing approximately 20 g, were purchased from Theodor Bilharz Research Institute (TBRI), and were equally randomized into five groups. Each group was treated with the corresponding treatment according to the experimental design for 14 consecutive days. To determine the effect of the compounds on immune cell population, blood was drawn by retro-orbital puncture after a treatment period. T lymphocytes subsets ($CD4^+$ and $CD8^+$) from peripheral blood were measured and analyzed on FACS Calibur.

Briefly, whole blood was drawn into EDTA pre-coated 1.5 mL tubes by rectal orbital puncture and mixed, and 100 µL added to the bottom of a well labelled tube and the appropriate primary antibody added to each tube; to a 100 µL sample was added 0.25 µg of FITC Hamster Anti-Mouse $CD4^+$, and 0.25 µg of APC Rat Anti-Mouse $CD8^+$ (Biolegend Co. Ltd). The tubes were capped, vortexed and incubated in the dark for 25 min at room temperature, followed by the addition of 1.5 mL lysing solution vortexed, incubated at room temperature in the dark for 10–15 min and centrifuged for 5 min at 200x *g*. The supernatant was aspirated; cells fixed by resuspending in 2% paraformaldehyde buffer for 30 min at room temperature and washed. The cells were finally suspended in 500 µL wash buffer and stored for 24 h at 4 °C in the dark. The samples were then analyzed on FACS Caliber (Merck-Millipore, Bio-Rad Beijing) with InCyte software version 2.2.2.

*Ethics statement for both animal models and for using volunteer blood cells:* All experimental protocols were approved and performed in compliance with the guide for the care and use of laboratory animals, published by the National Institutes of Health (USA), and performed according to Institutional guidelines. On the other hand, all volunteers gave their consent by signing a consent form before they participated.

**Figures of the inhibition zone of the most promising derivatives**

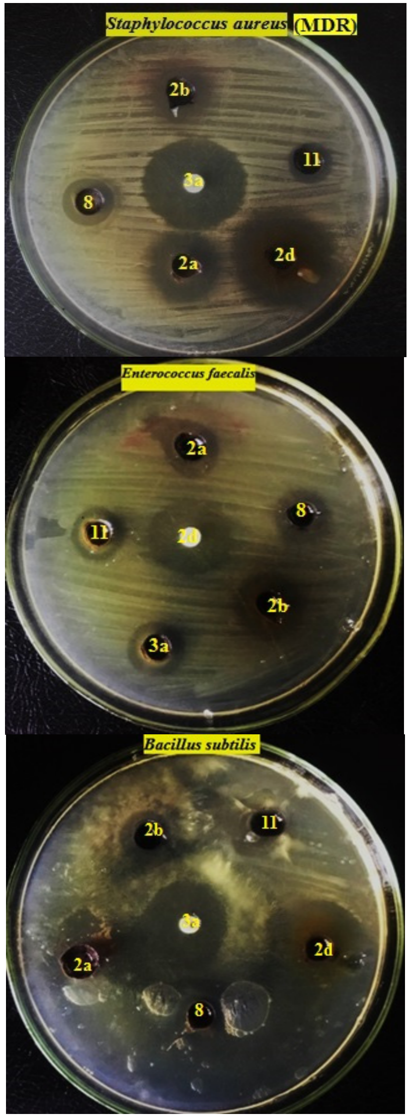

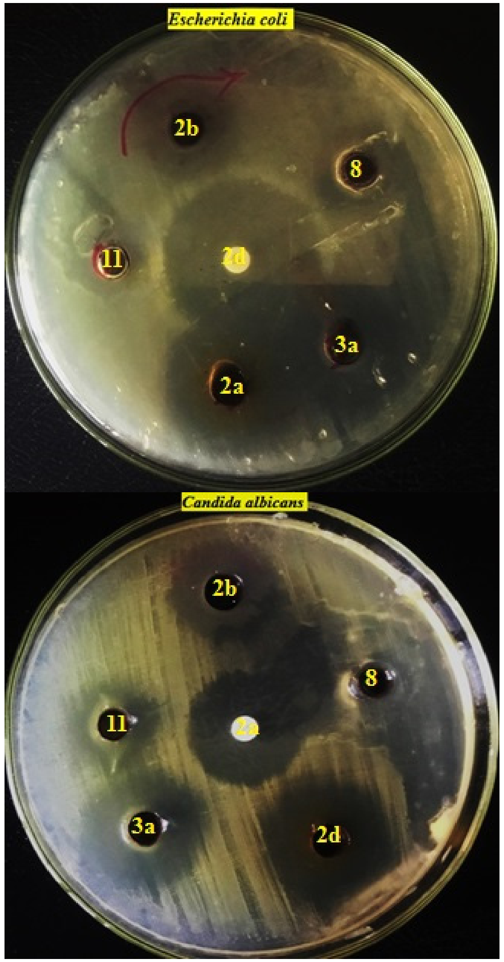
Escherichia coli
2b
8
11
2a
3a
Candida albicans
2b
8
11
3a
2d

**Molecular Docking Study**

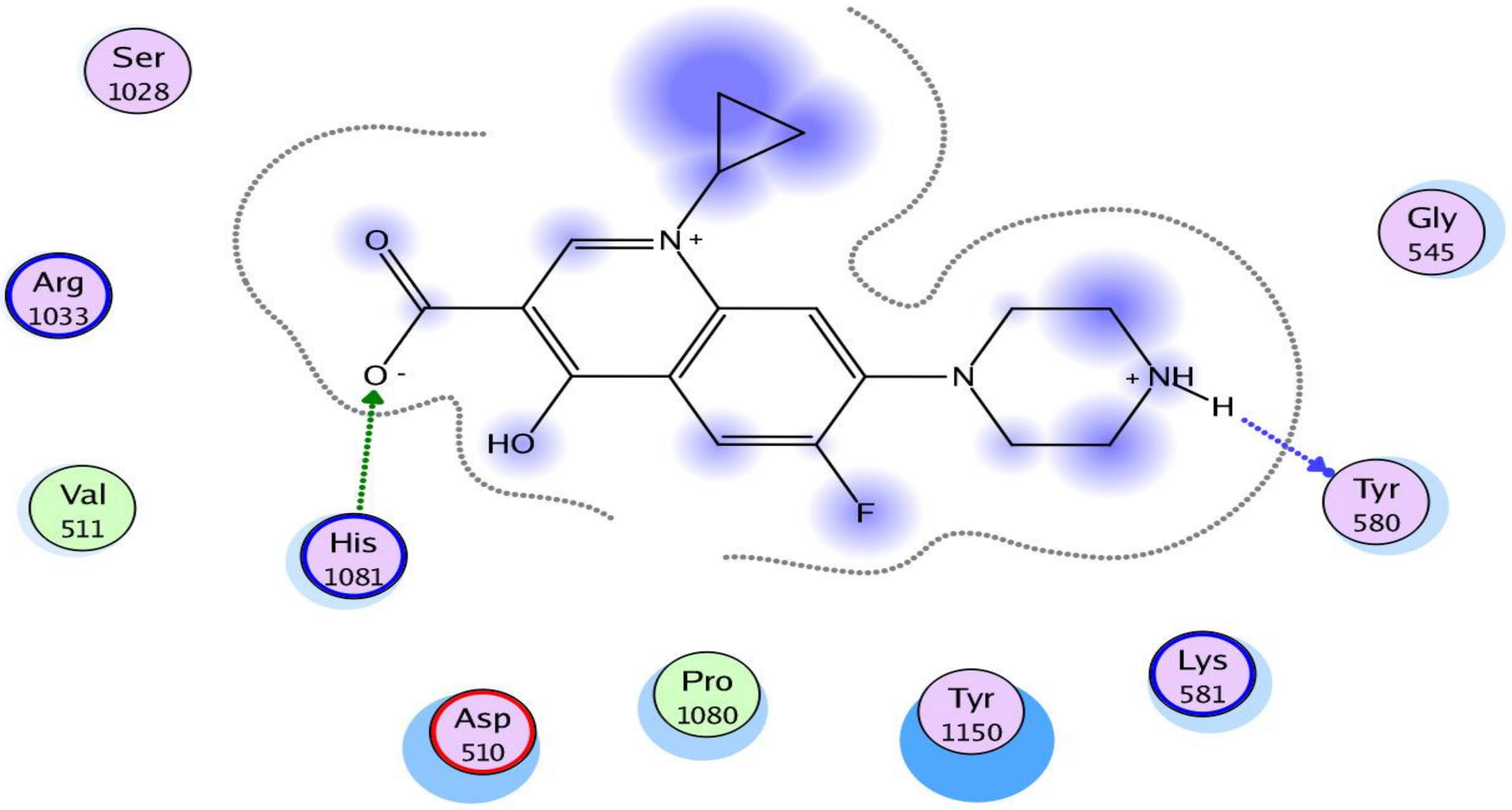


**2D structure of Ciprofloxacin inside the active site DNA gyrase (2XCT).**

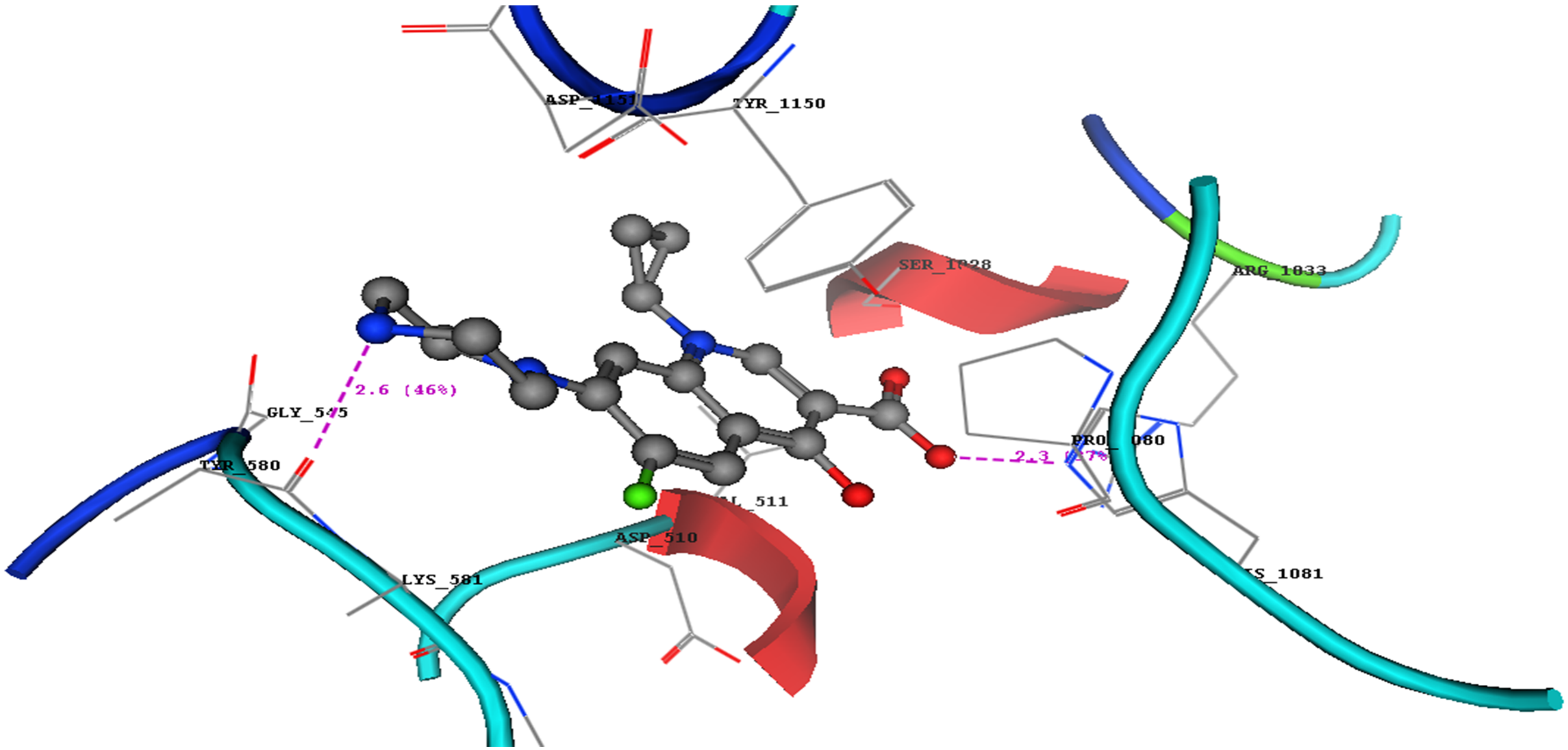


**3D structure of Ciprofloxacin inside the active site DNA gyrase (2XCT).**

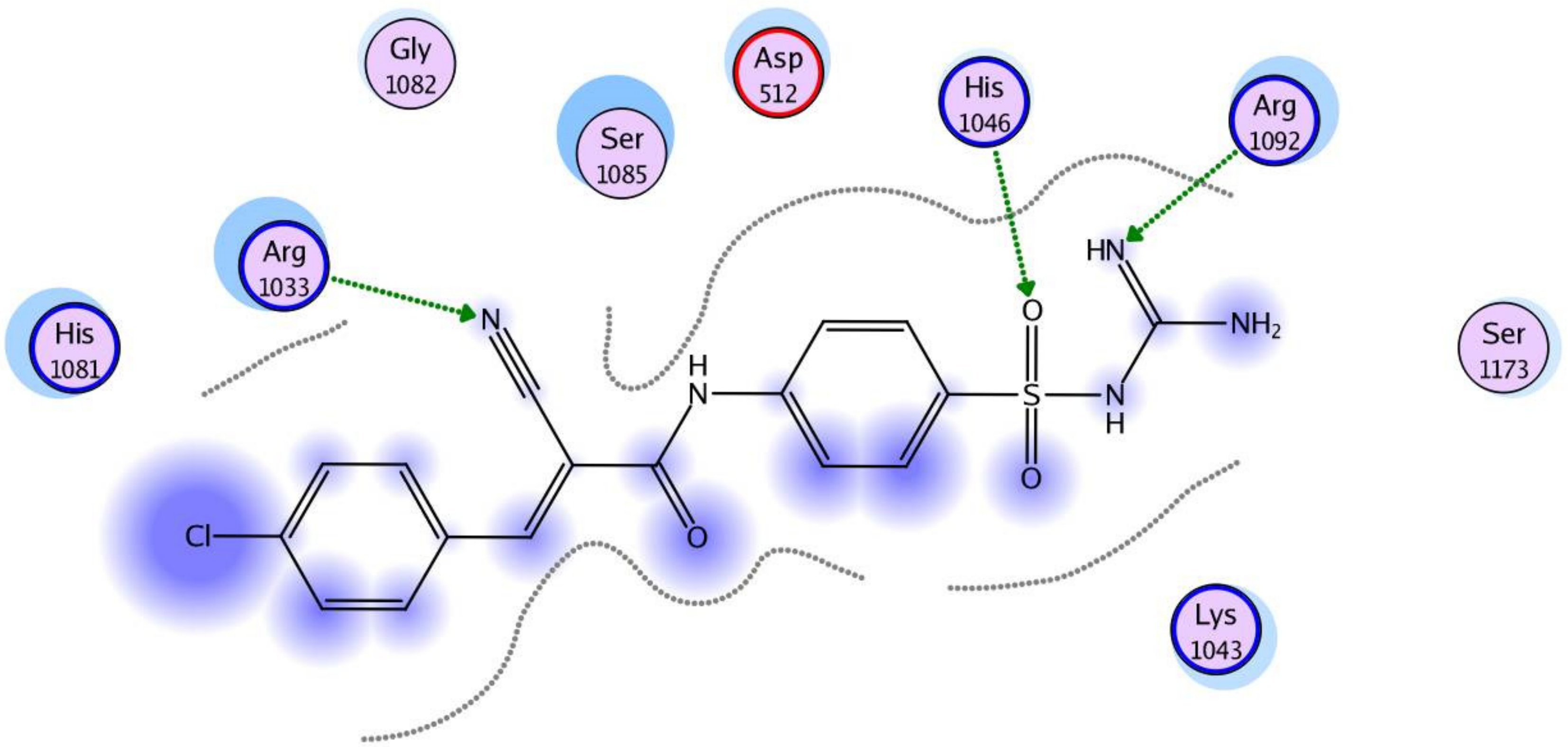


**2D structure of compound 2a inside the active site DNA gyrase (2XCT).**

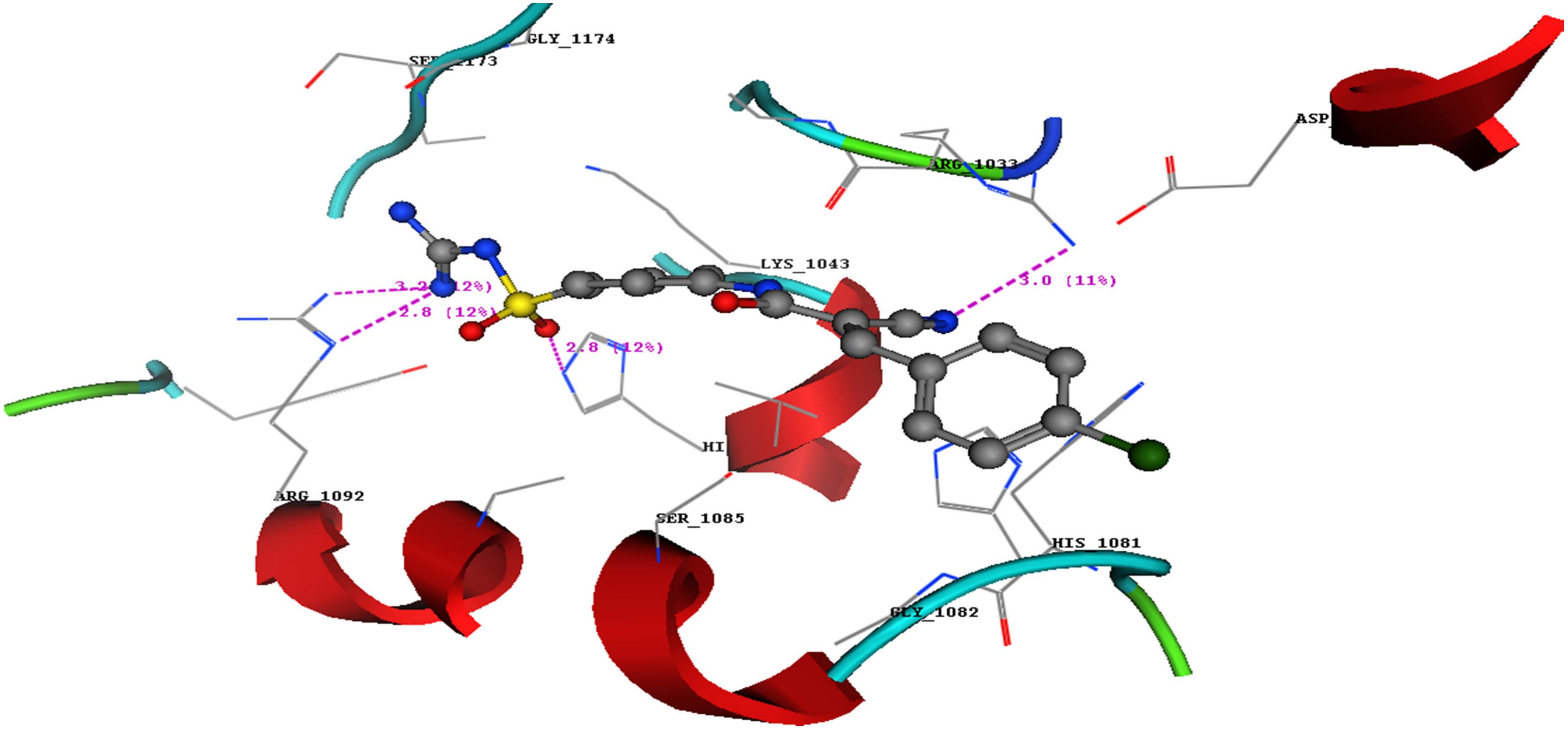


**3D structure of compound 2a inside the active site DNA gyrase (2XCT).**

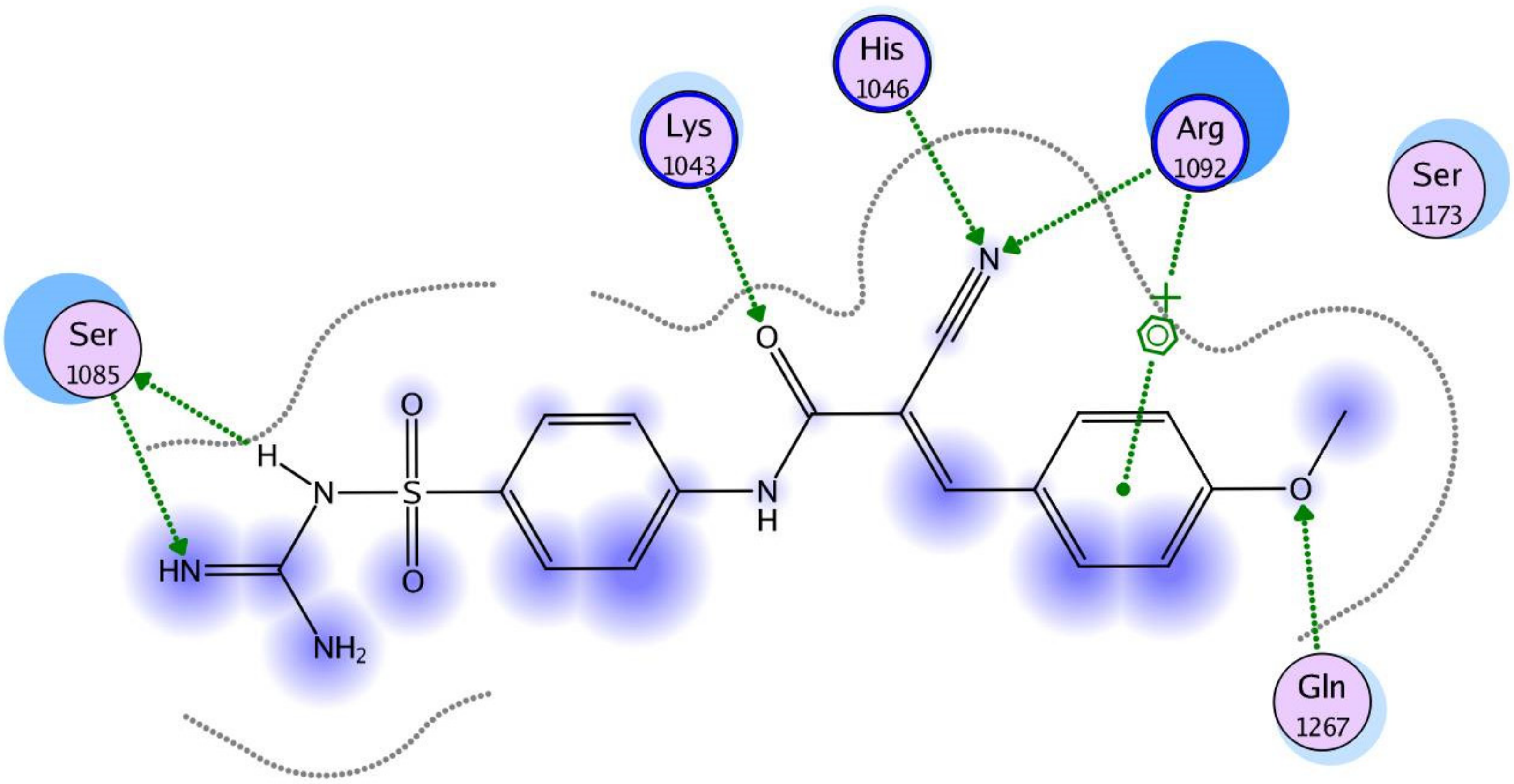


**2D structure of compound 2b inside the active site DNA gyrase (2XCT).**

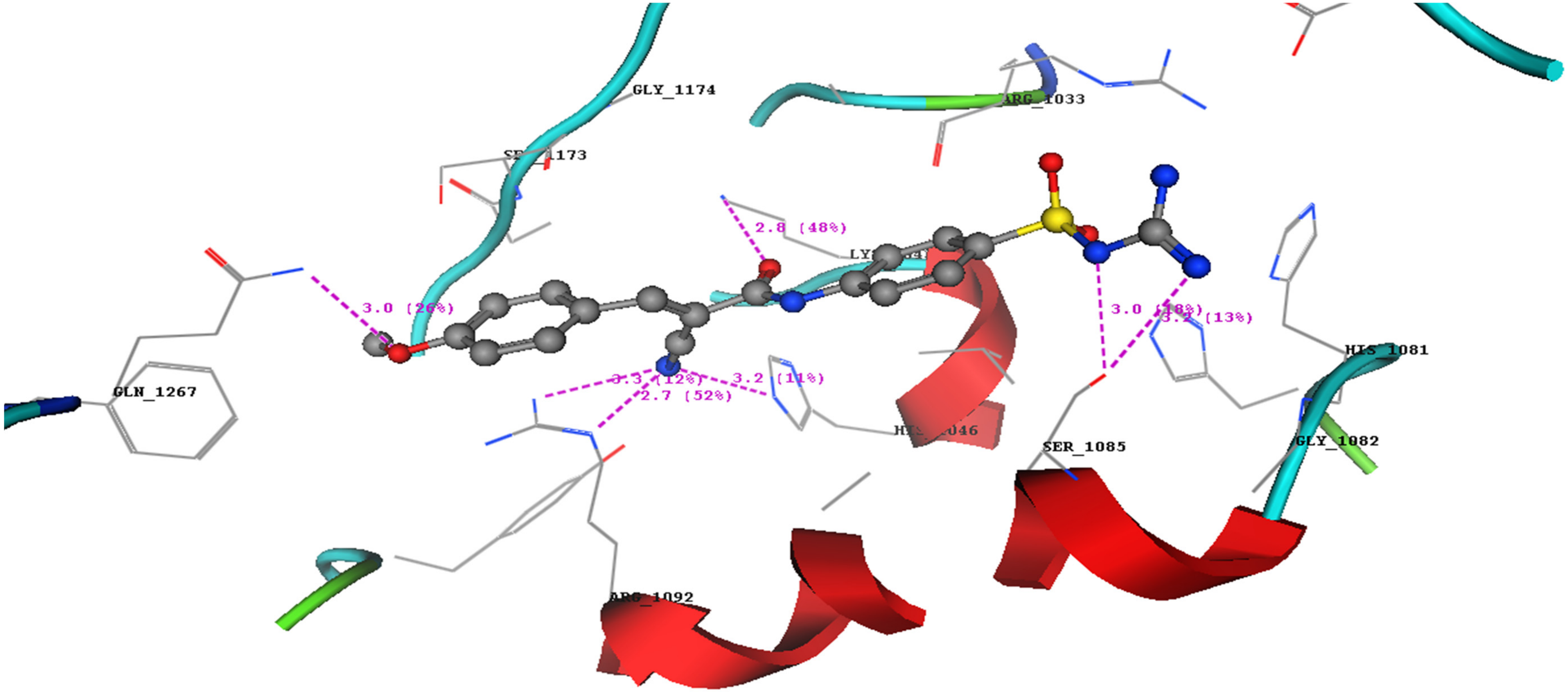


**3D structure of compound 2b inside the active site DNA gyrase (2XCT).**

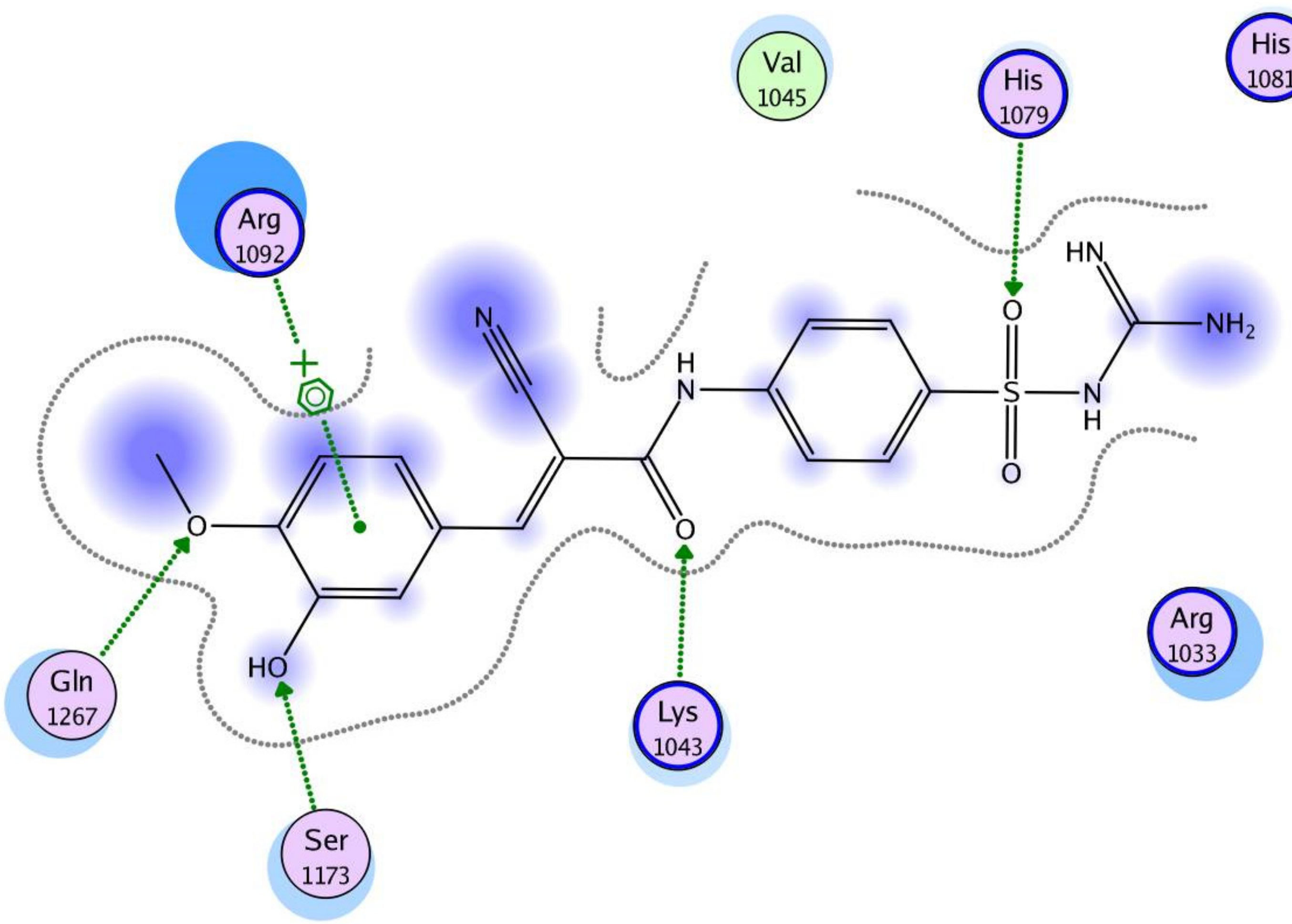


**2D structure of compound 2d inside the active site DNA gyrase (2XCT).**

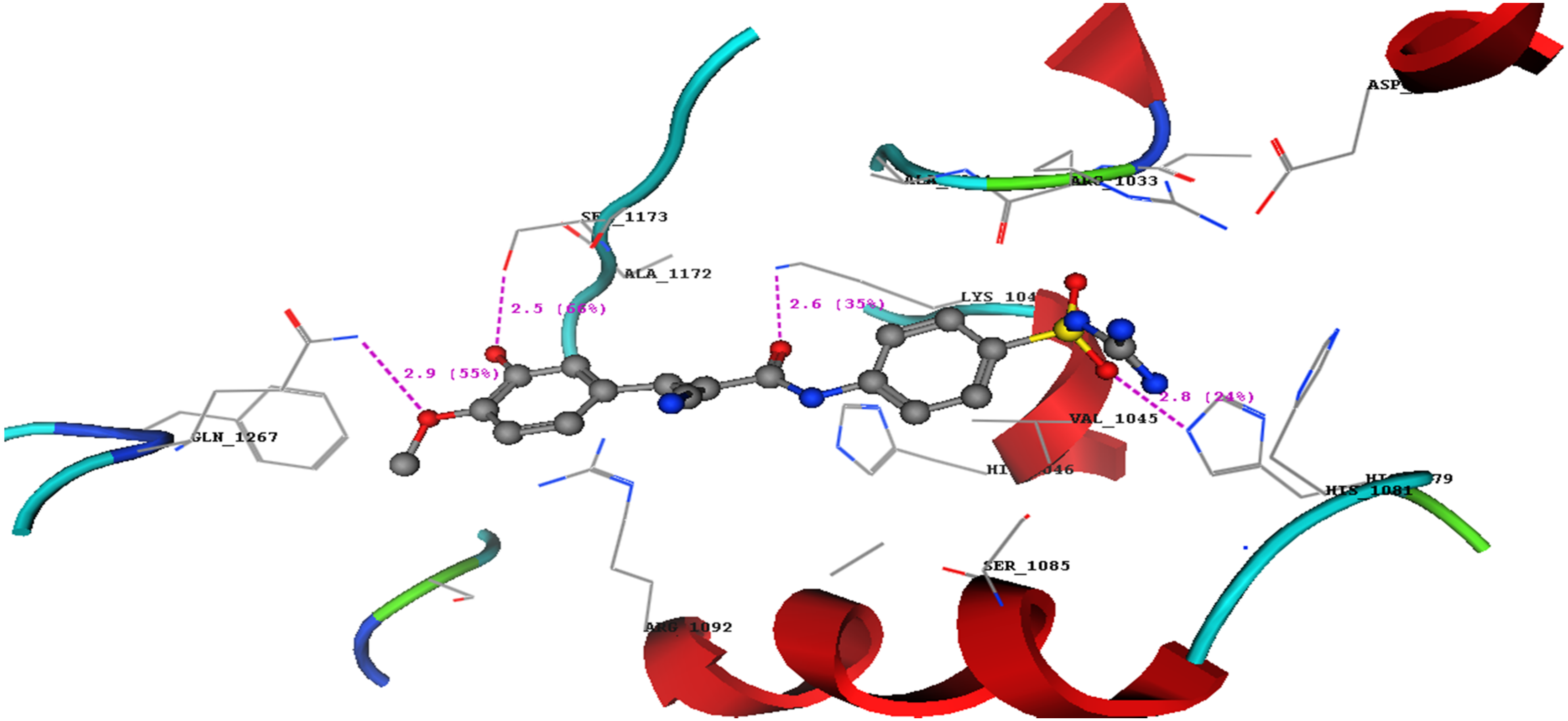


**3D structure of compound 2d inside the active site DNA gyrase (2XCT).**

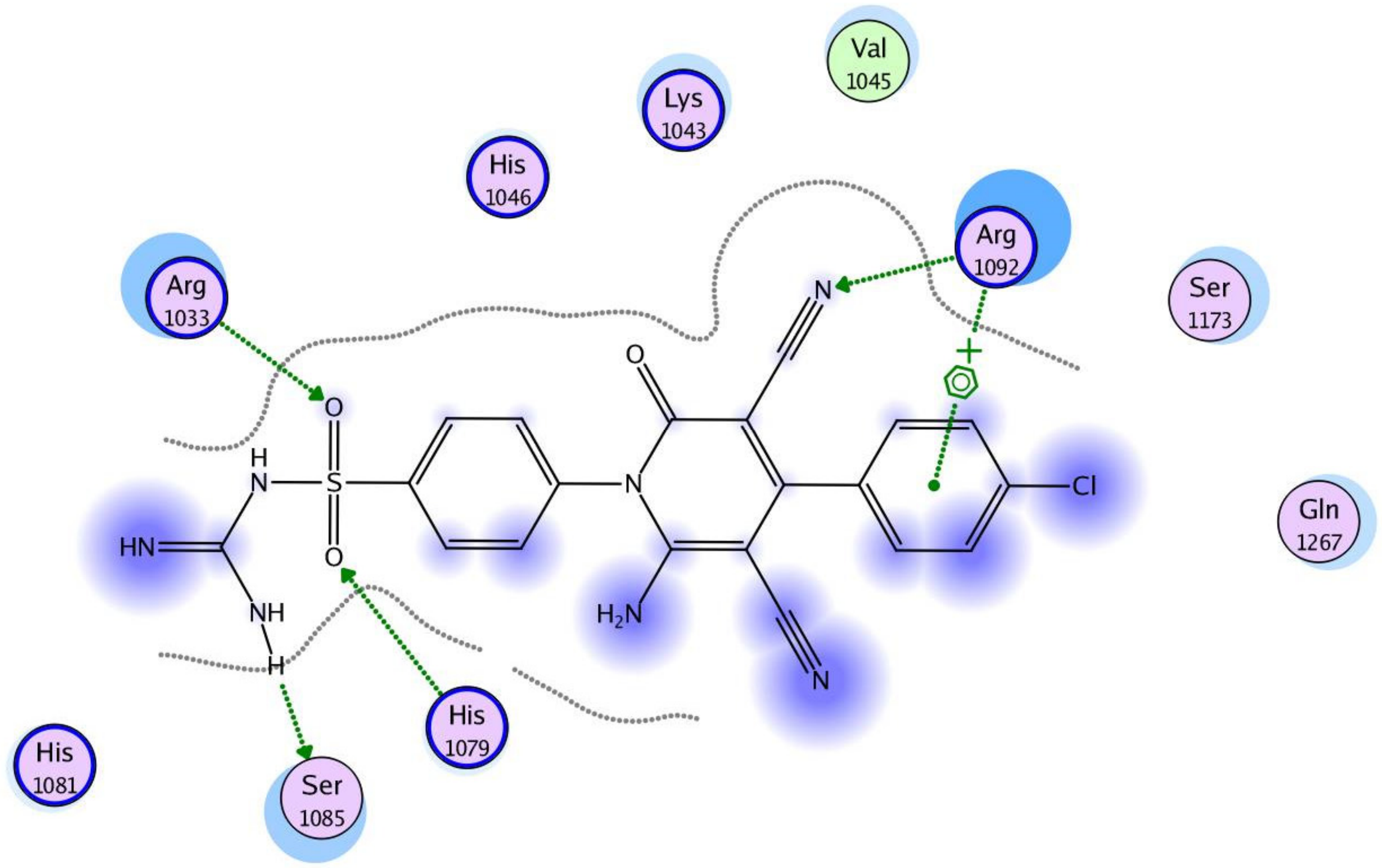


**2D structure of compound 3a inside the active site DNA gyrase (2XCT).**

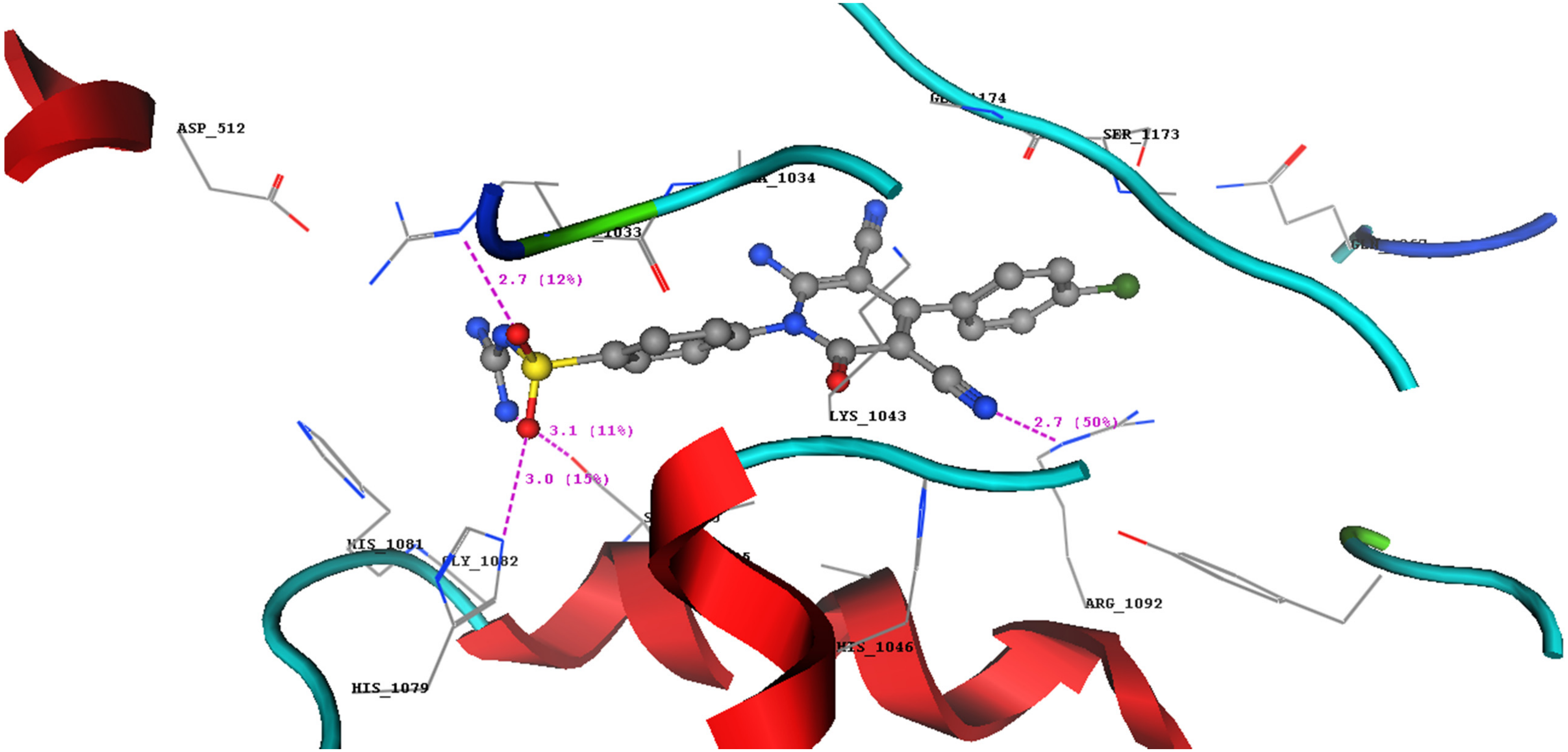


**3D structure of compound 3a inside the active site DNA gyrase (2XCT).**

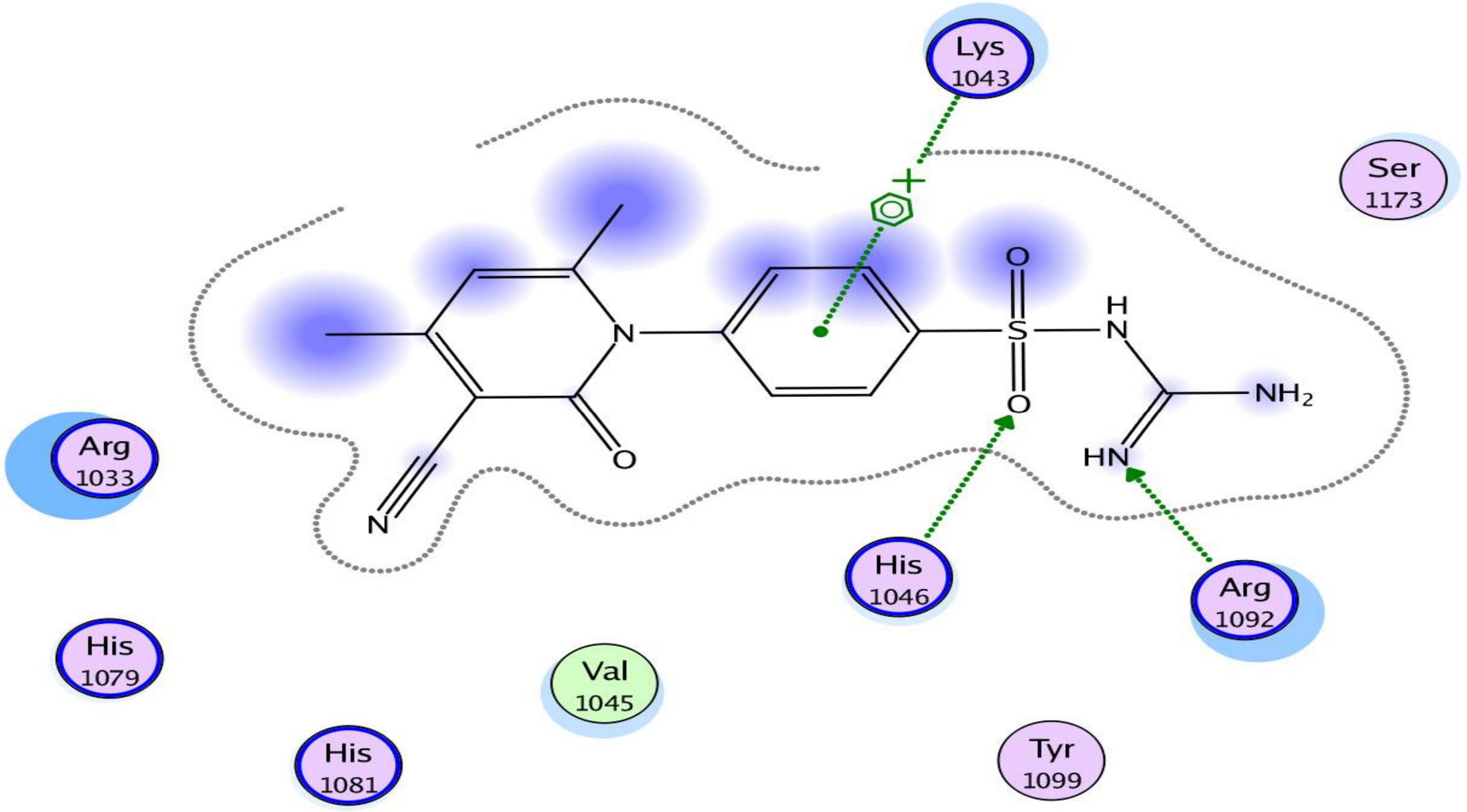


**2D structure of compound 8 inside the active site DNA gyrase (2XCT).**

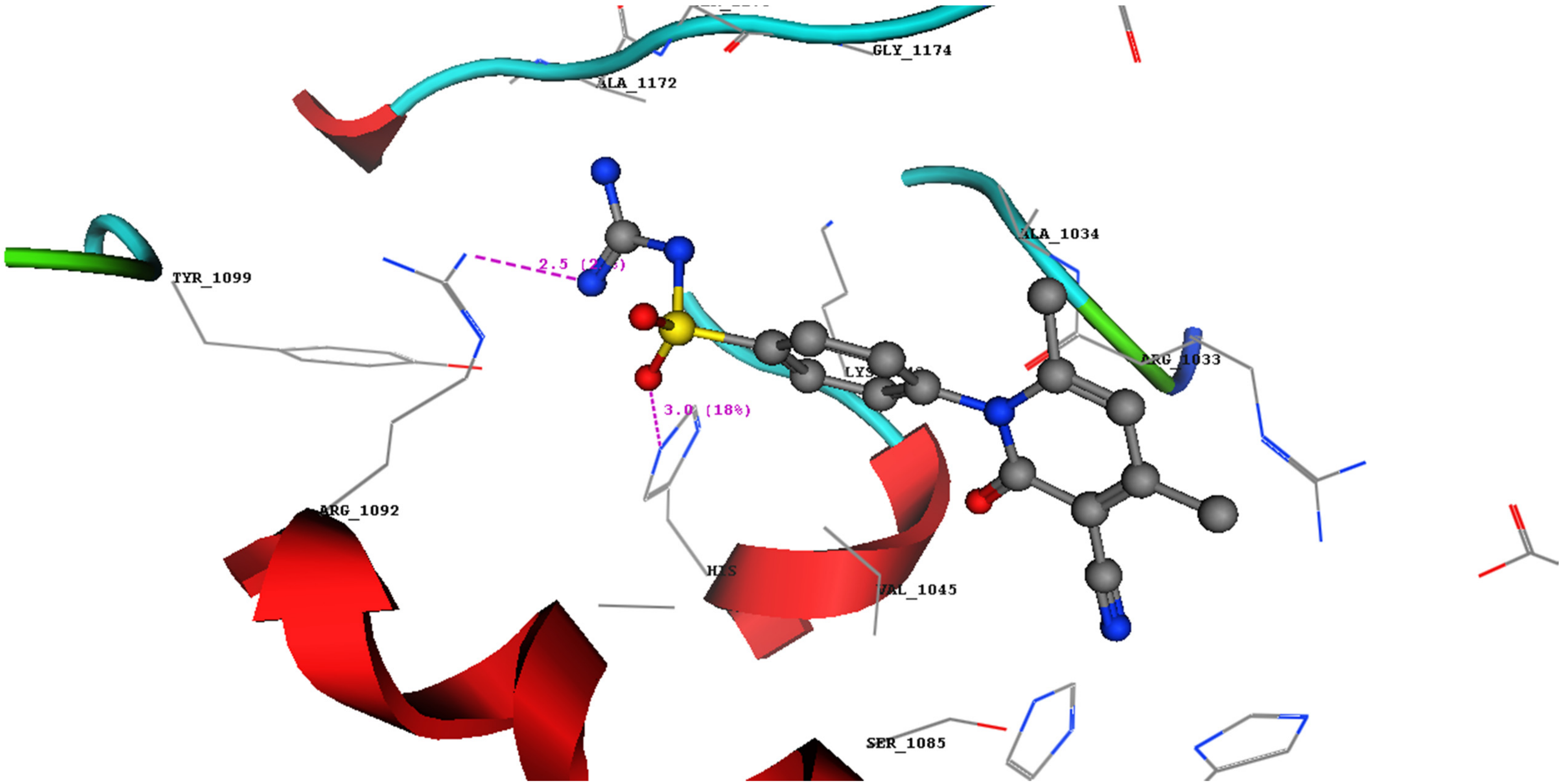


**3D structure of compound 8 inside the active site DNA gyrase (2XCT).**

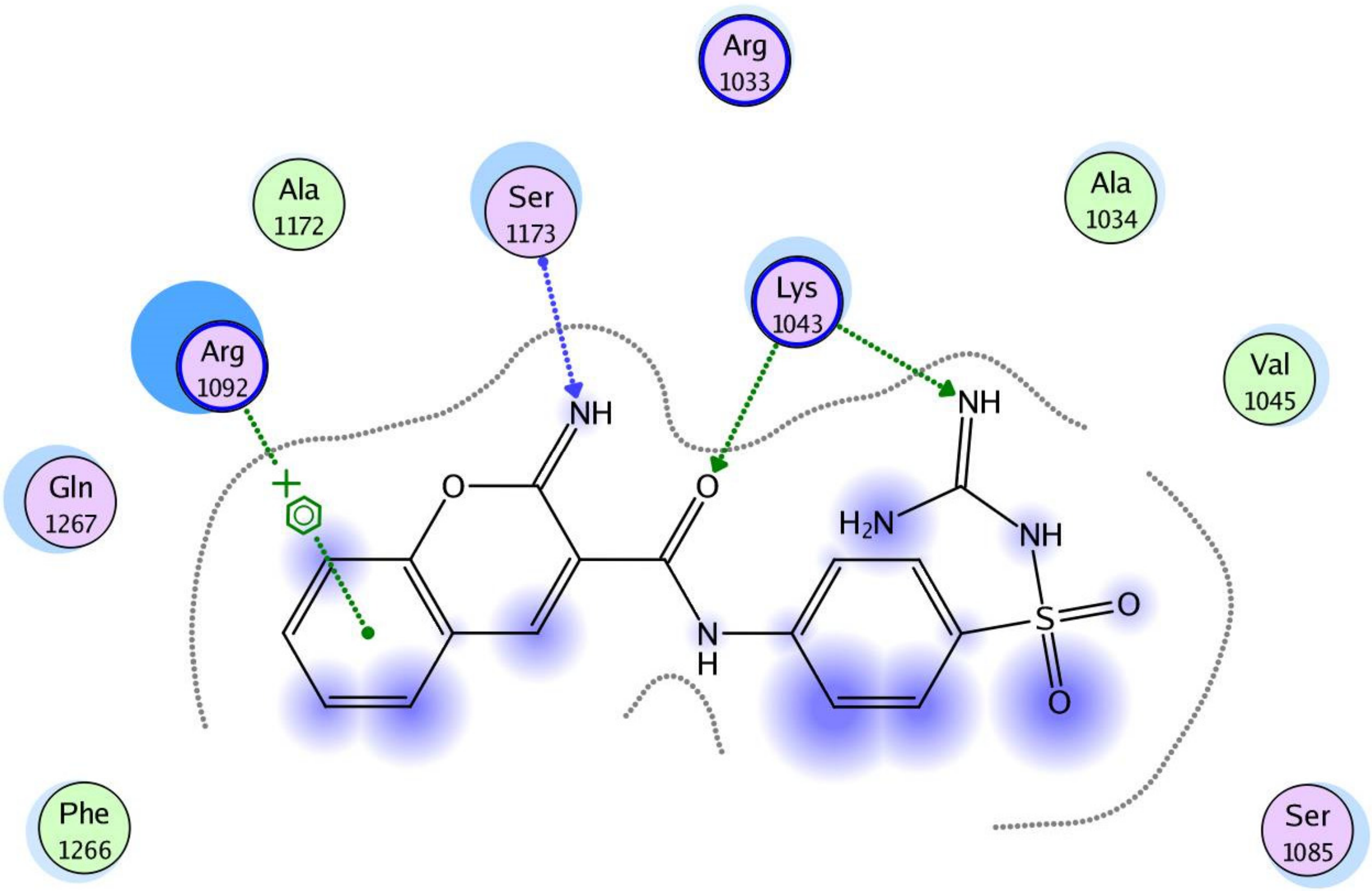


**2D structure of compound 11 inside the active site DNA gyrase (2XCT).**

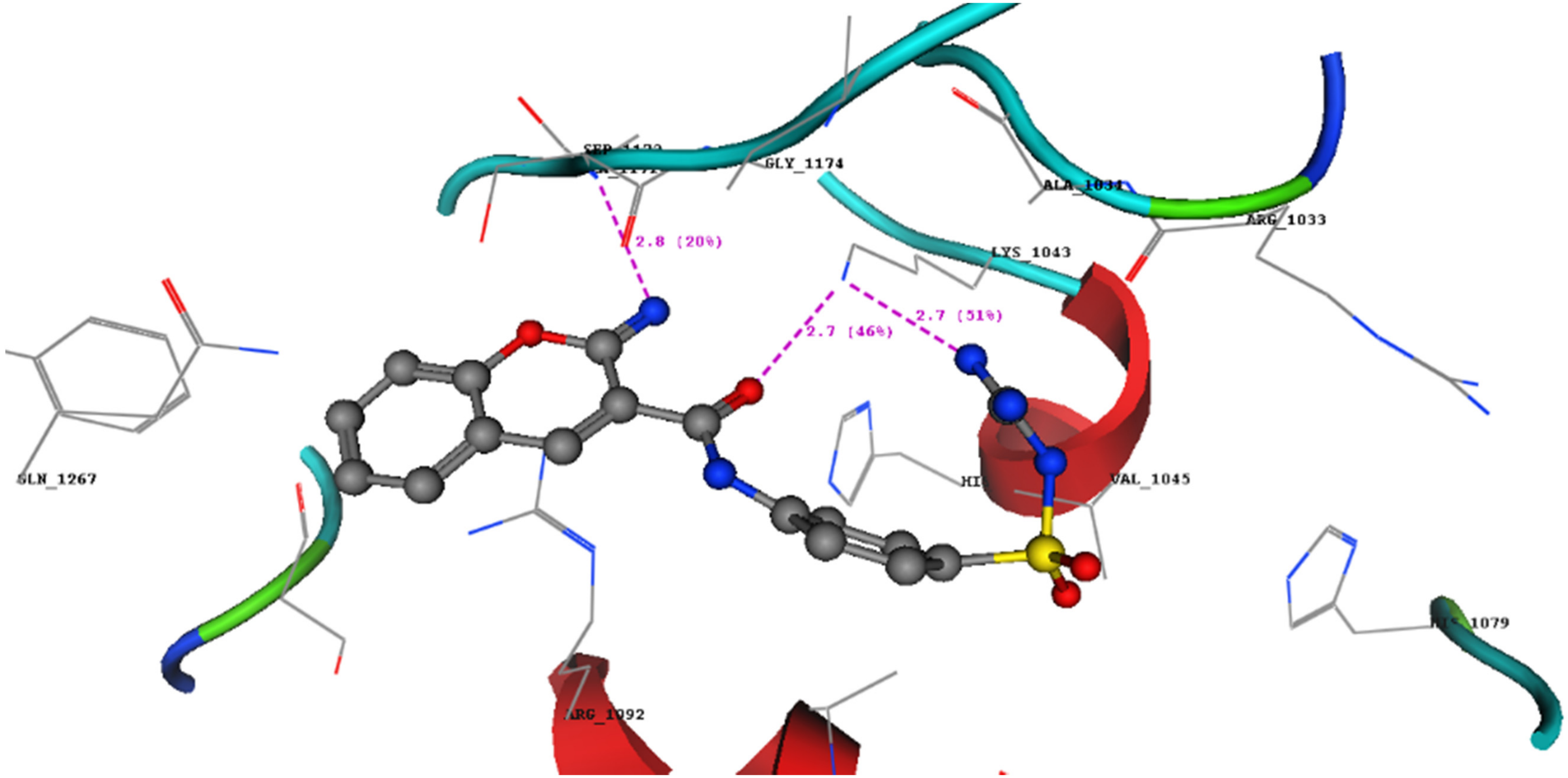


**3D structure of compound 11 inside the active site DNA gyrase (2XCT).**

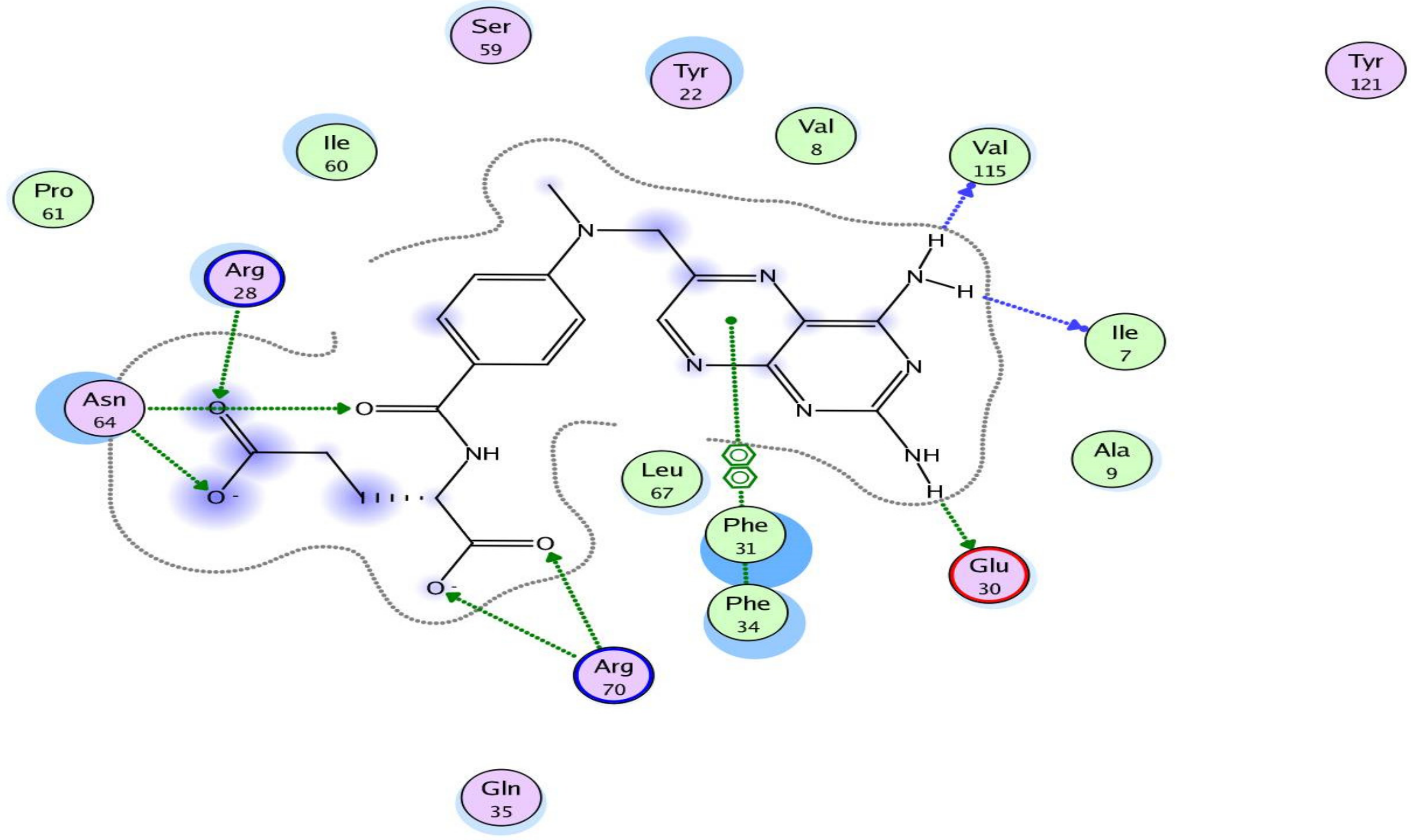


**2D structure of Methotrexate (MTX) inside the active site DHGR (1DLS).**

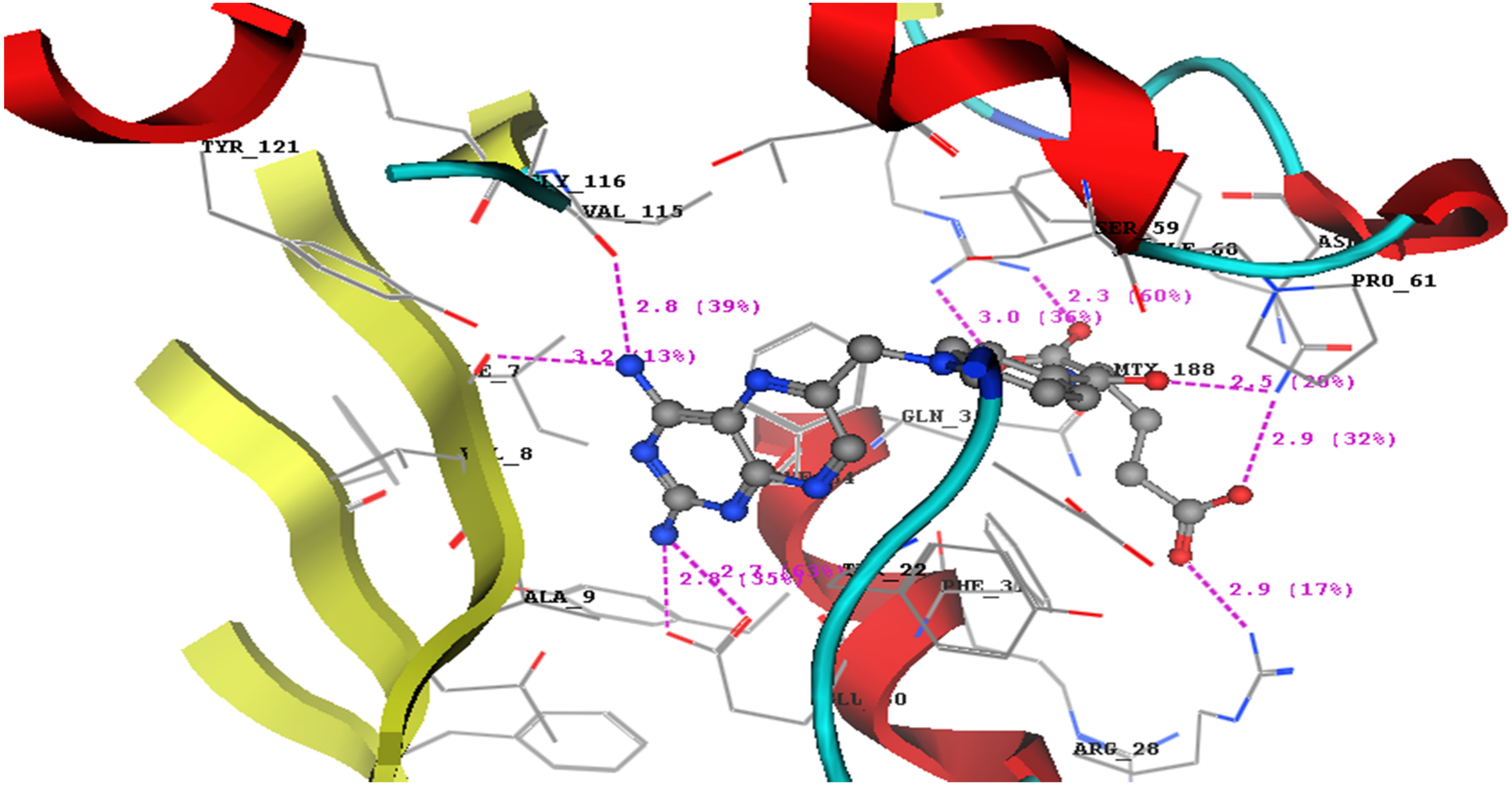


**3D structure of Methotrexate (MTX) inside the active site DHGR (1DLS).**

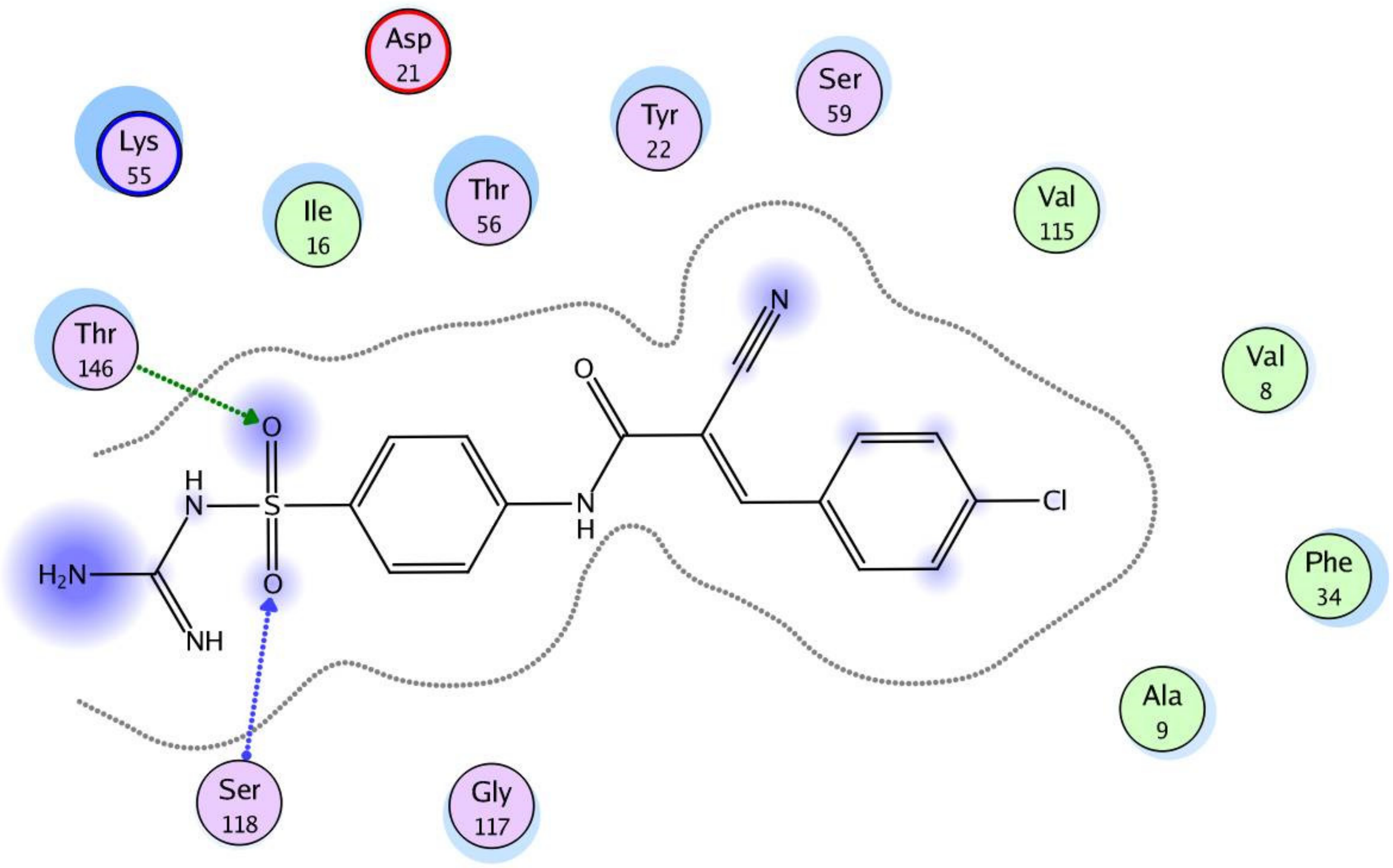


**2D structure of compound 2a inside the active site DHGR (1DLS).**

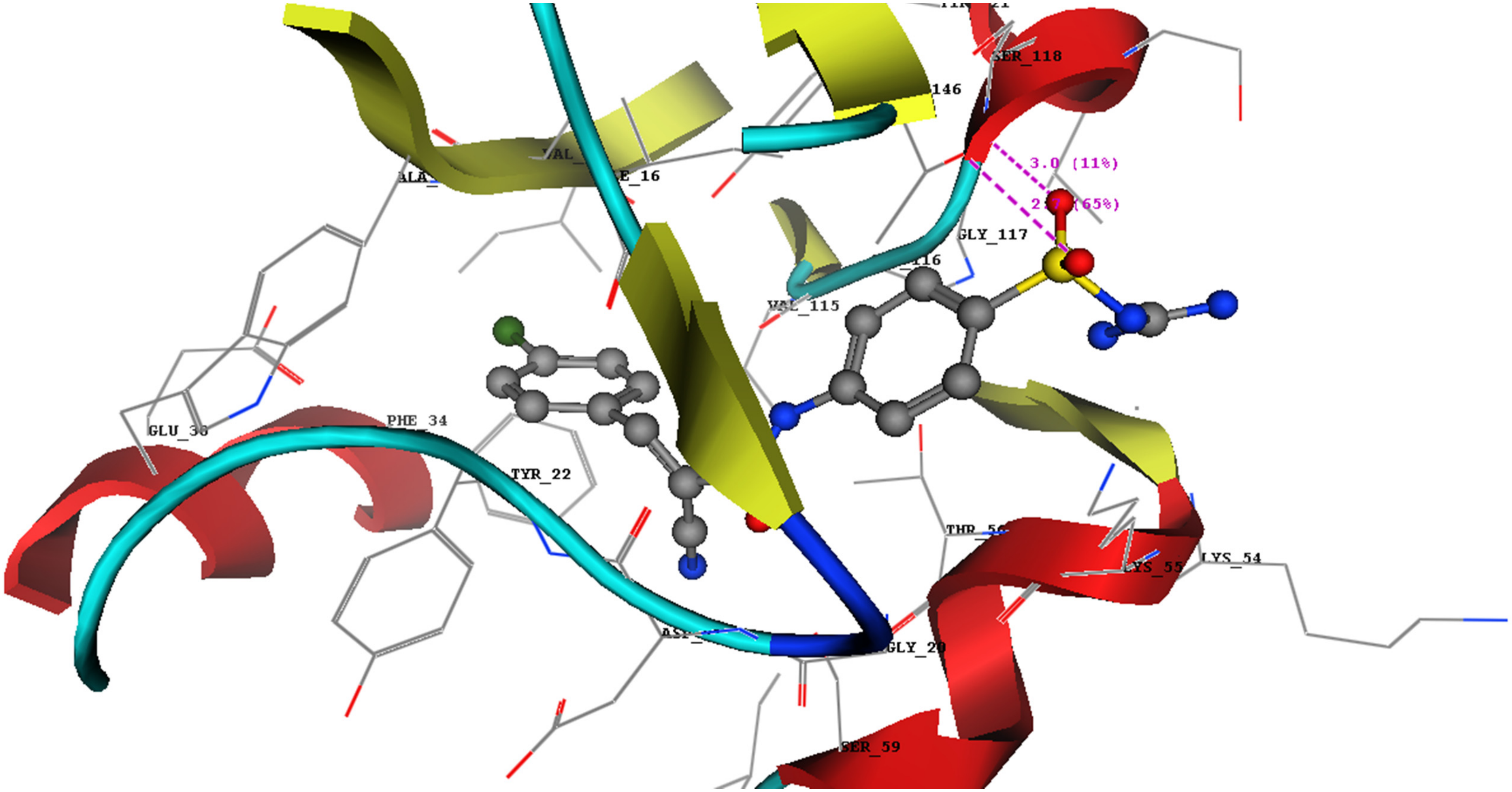


**3D structure of compound 2a inside the active site DHGR (1DLS).**

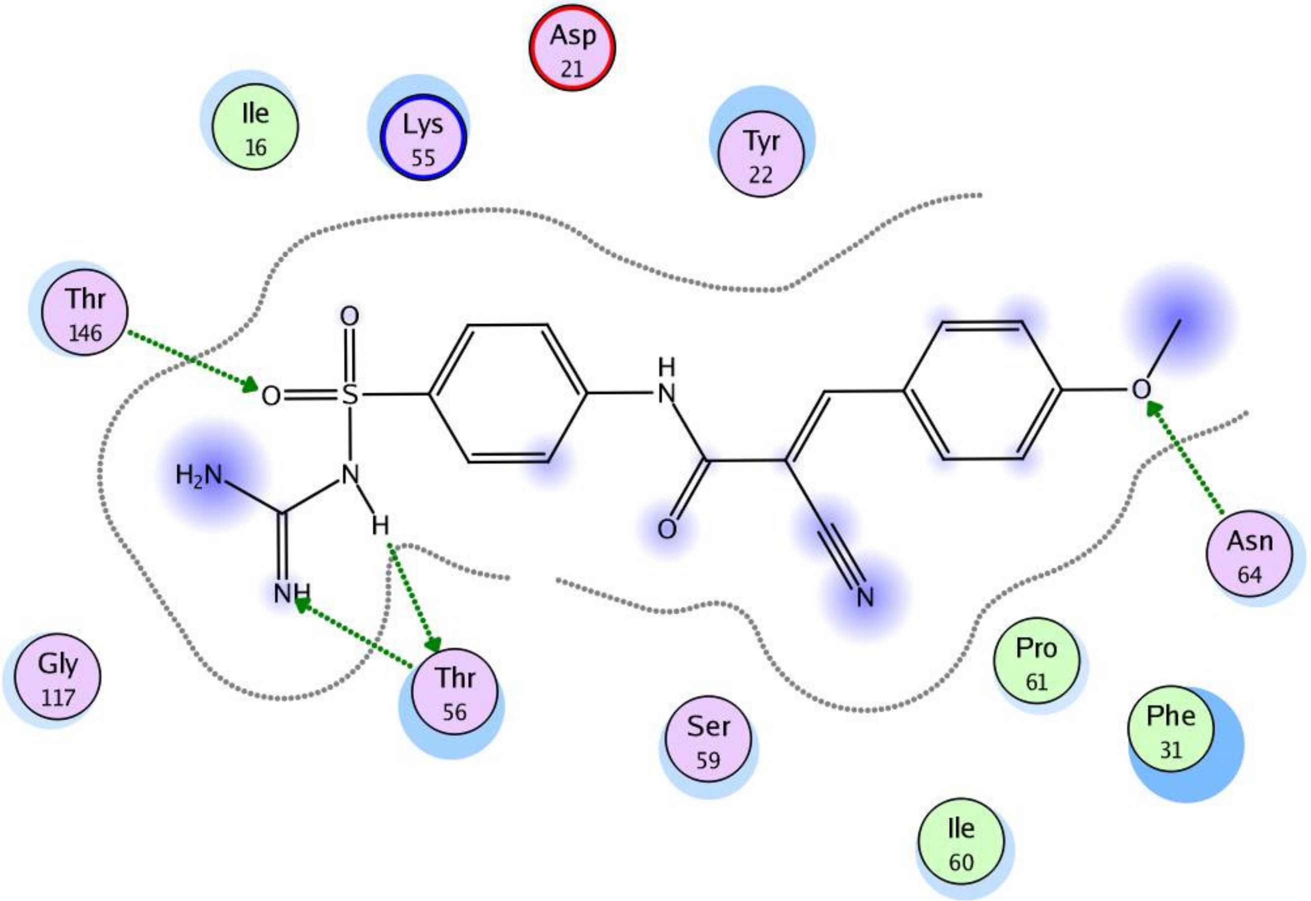


**2D structure of compound 2b inside the active site DHGR (1DLS).**

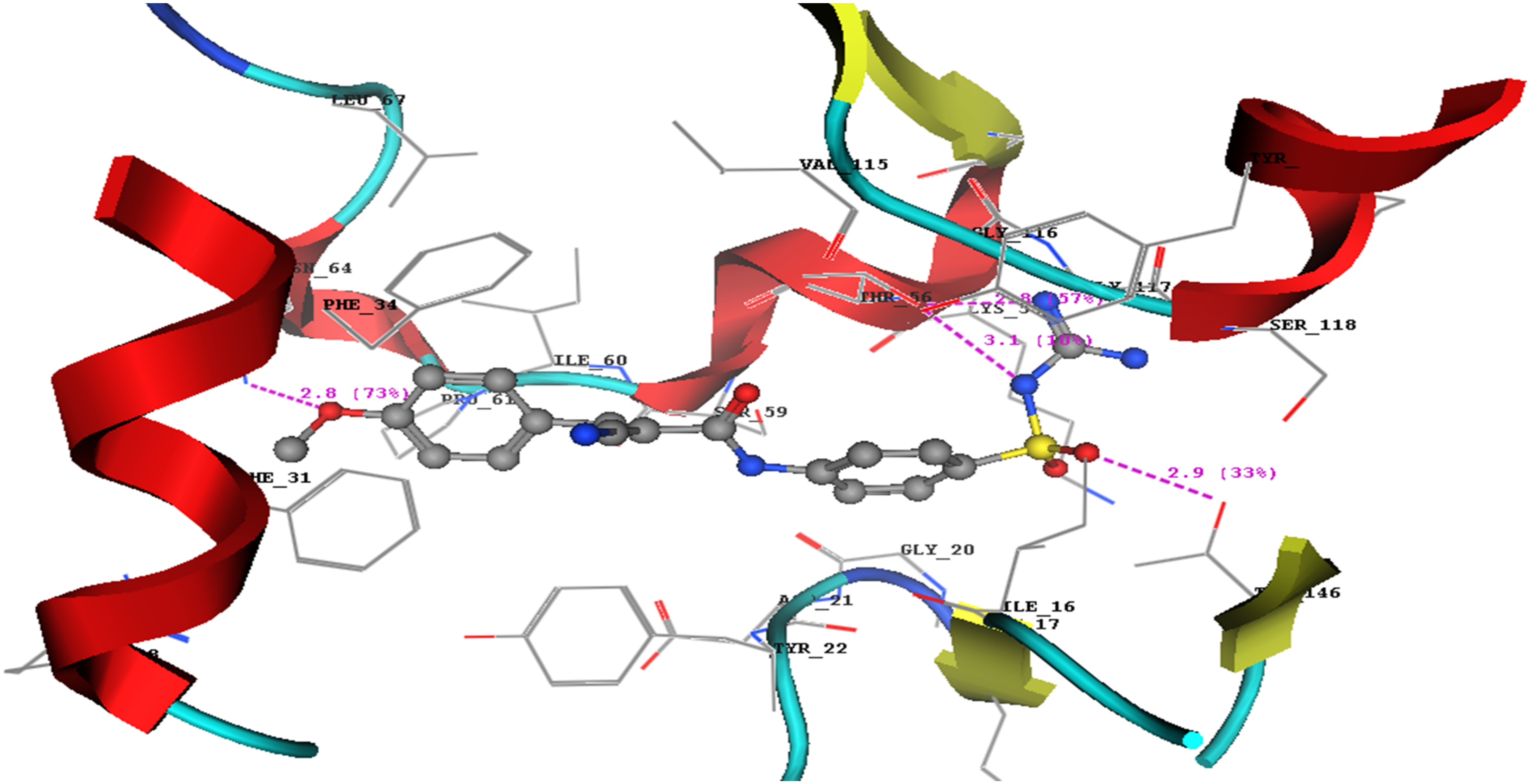


**3D structure of compound 2b inside the active site DHGR (1DLS).**

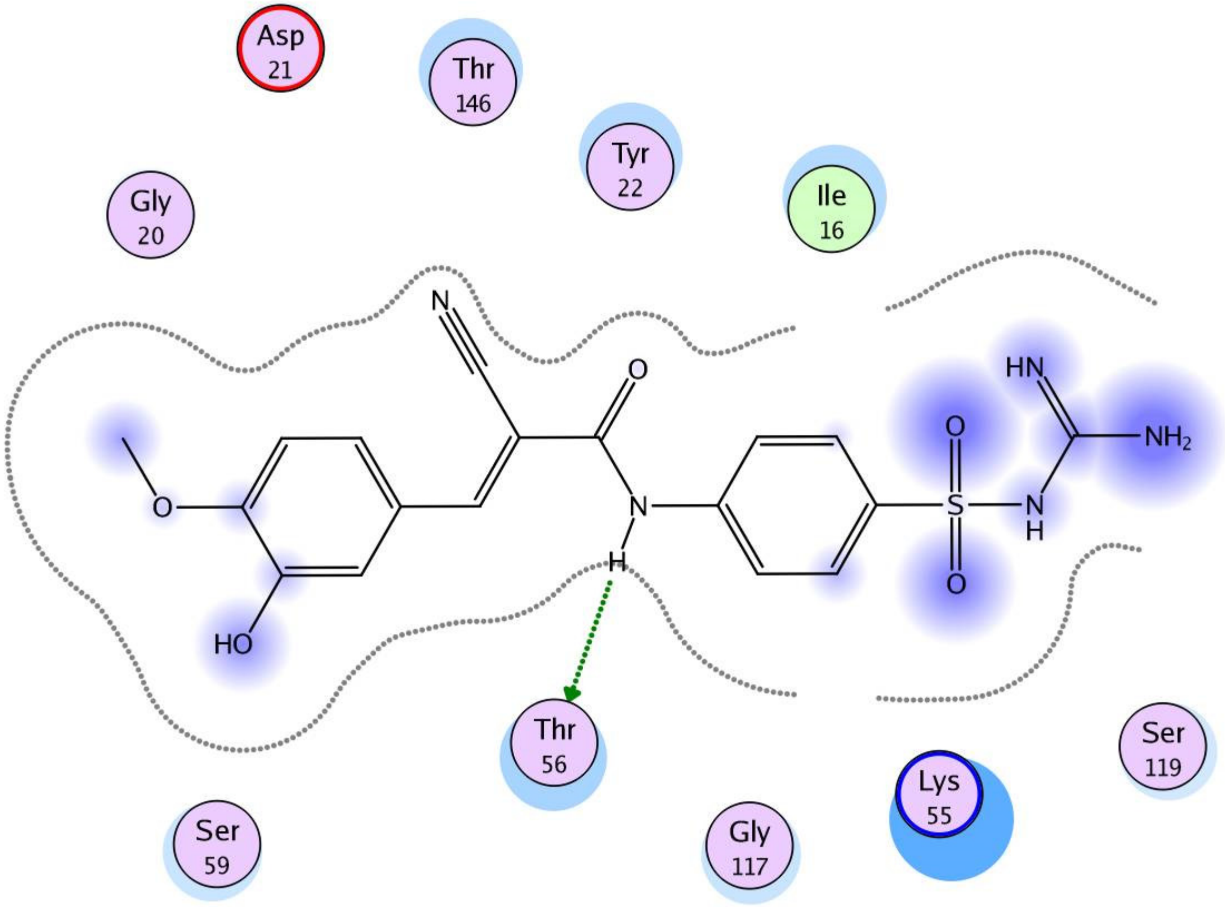


**2D structure of compound 2d inside the active site DHGR (1DLS).**

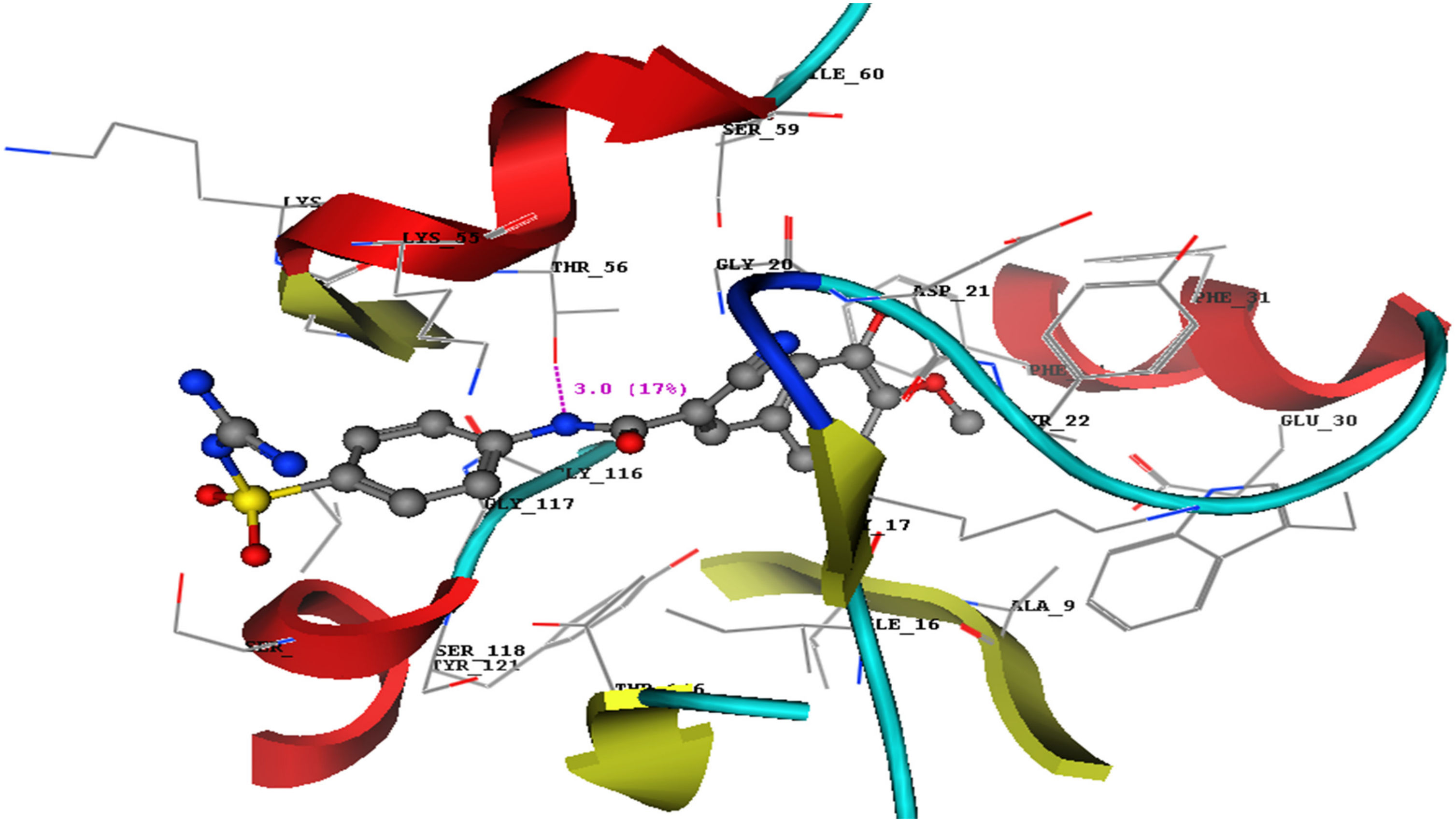


**3D structure of compound 2d inside the active site DHGR (1DLS).**

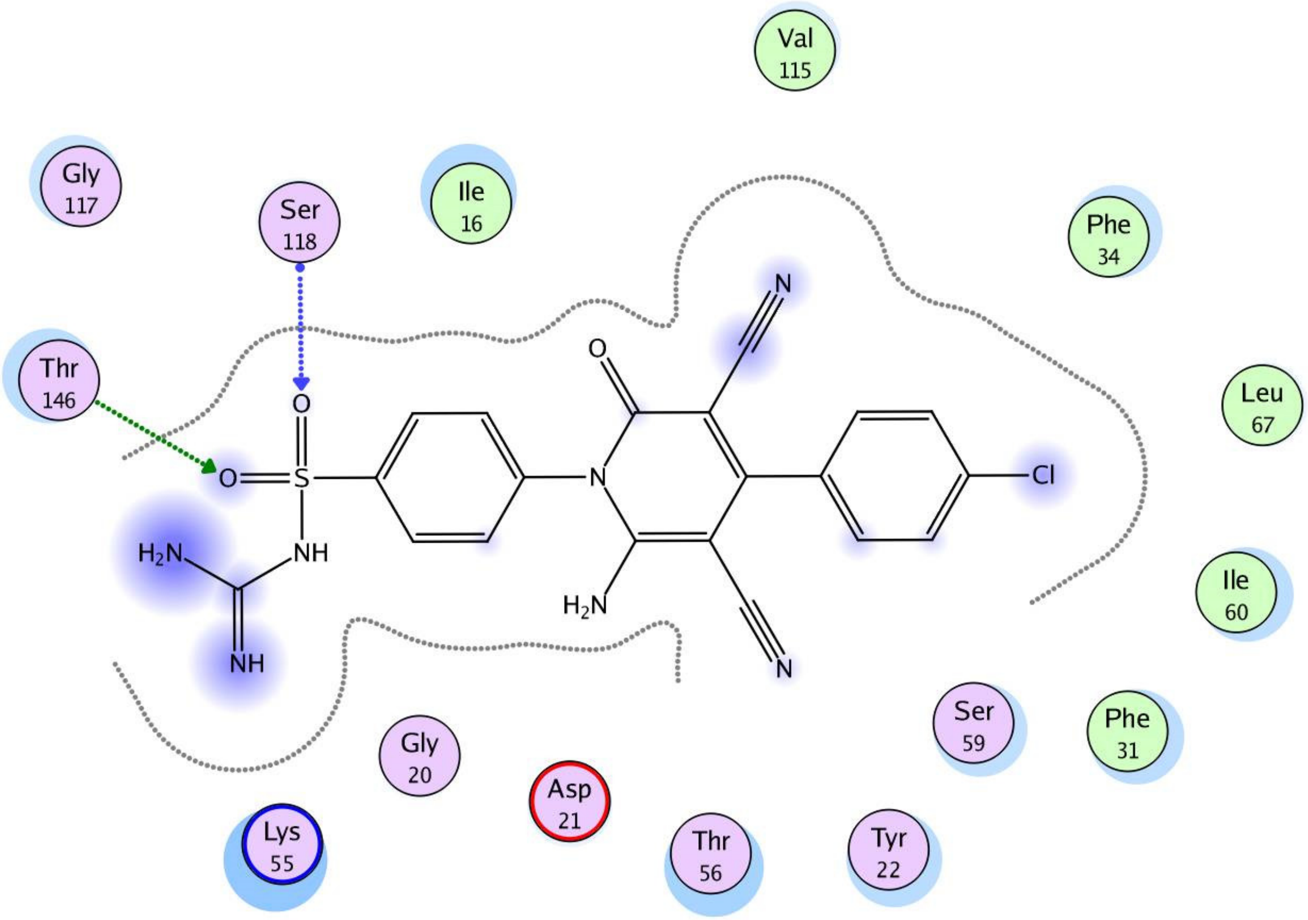


**2D structure of compound 3a inside the active site DHGR (1DLS).**

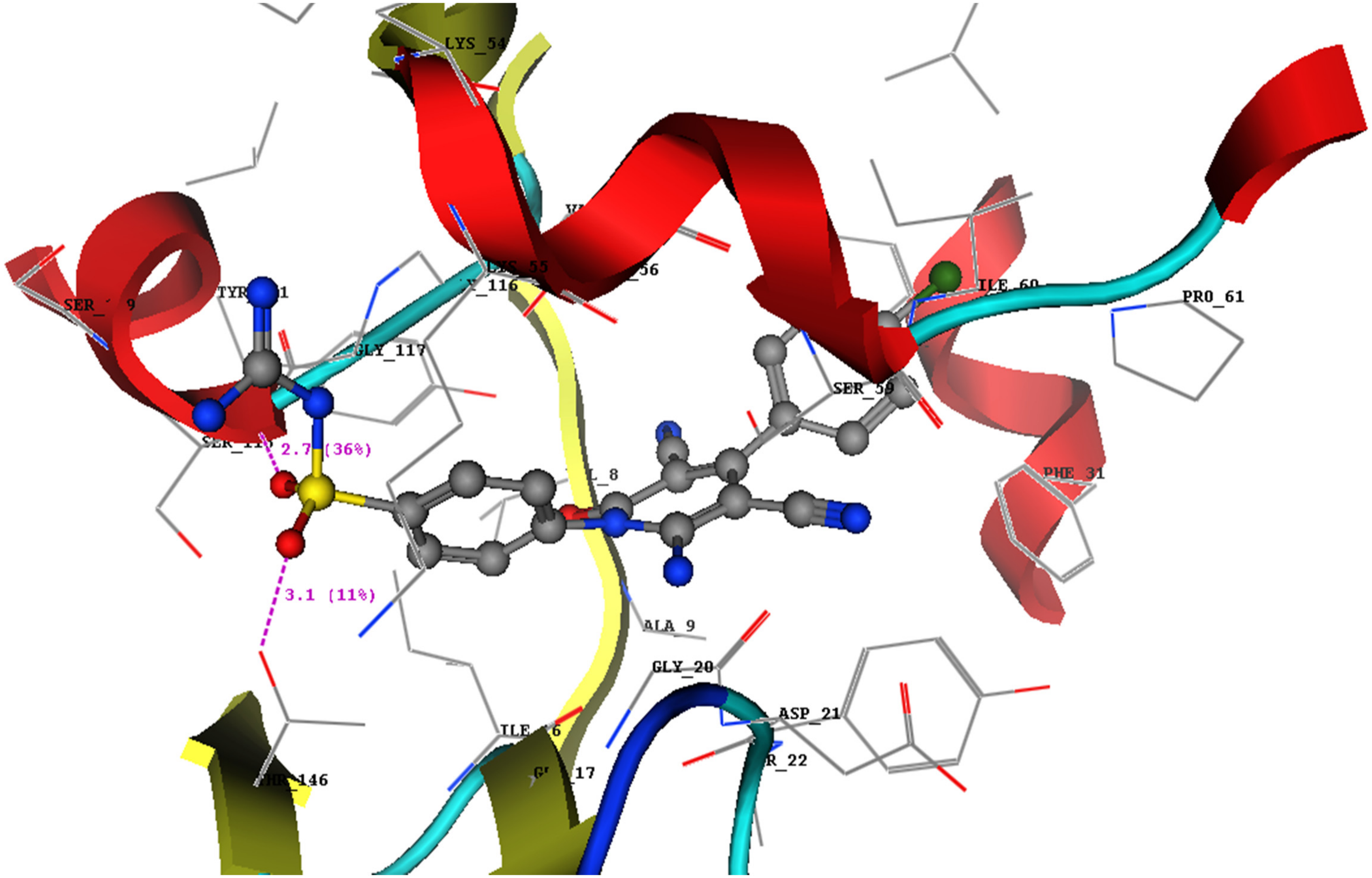


**3D structure of compound 3a inside the active site DHGR (1DLS).**

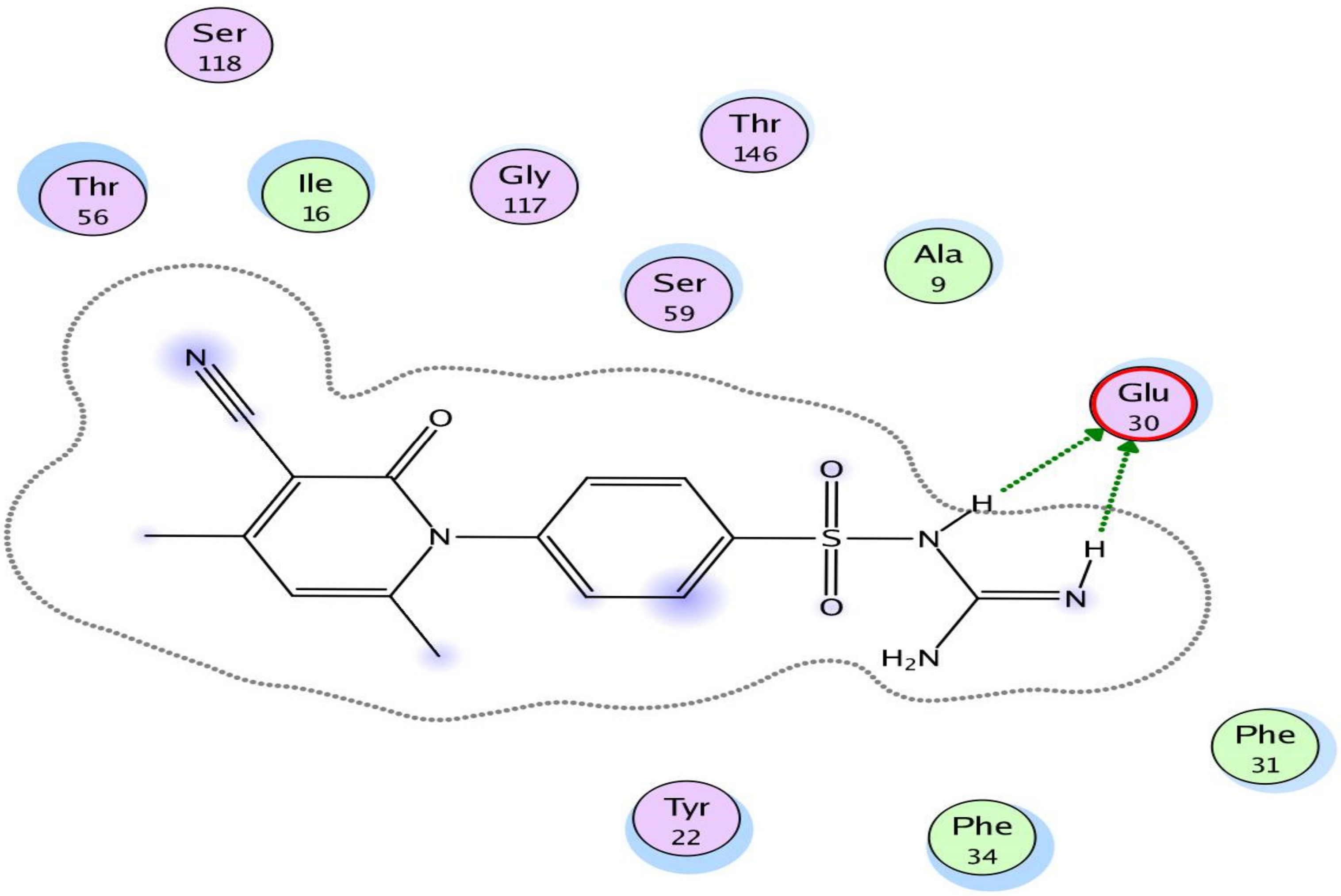


**2D structure of compound 8 inside the active site DHGR (1DLS).**

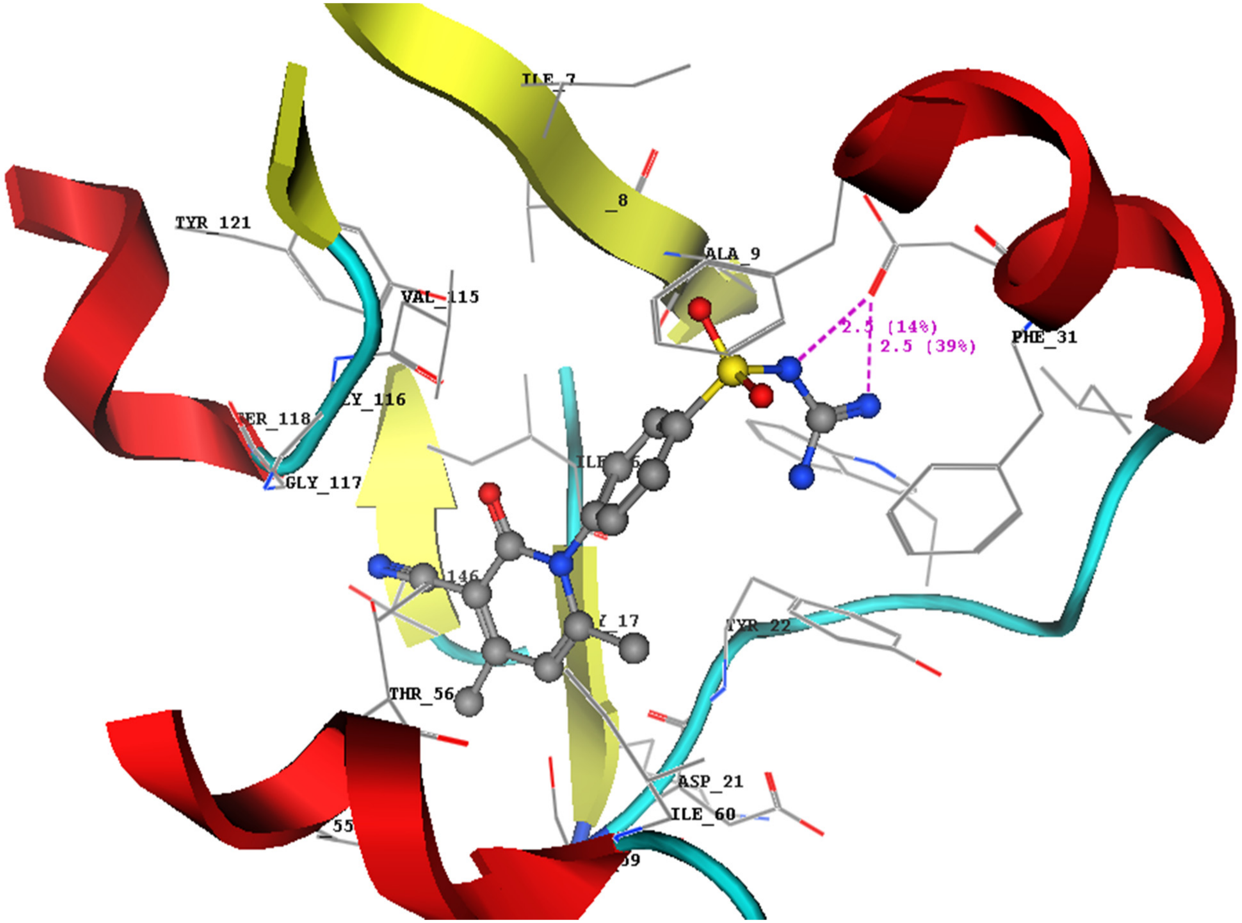


**3D structure of compound 8 inside the active site DHGR (1DLS).**

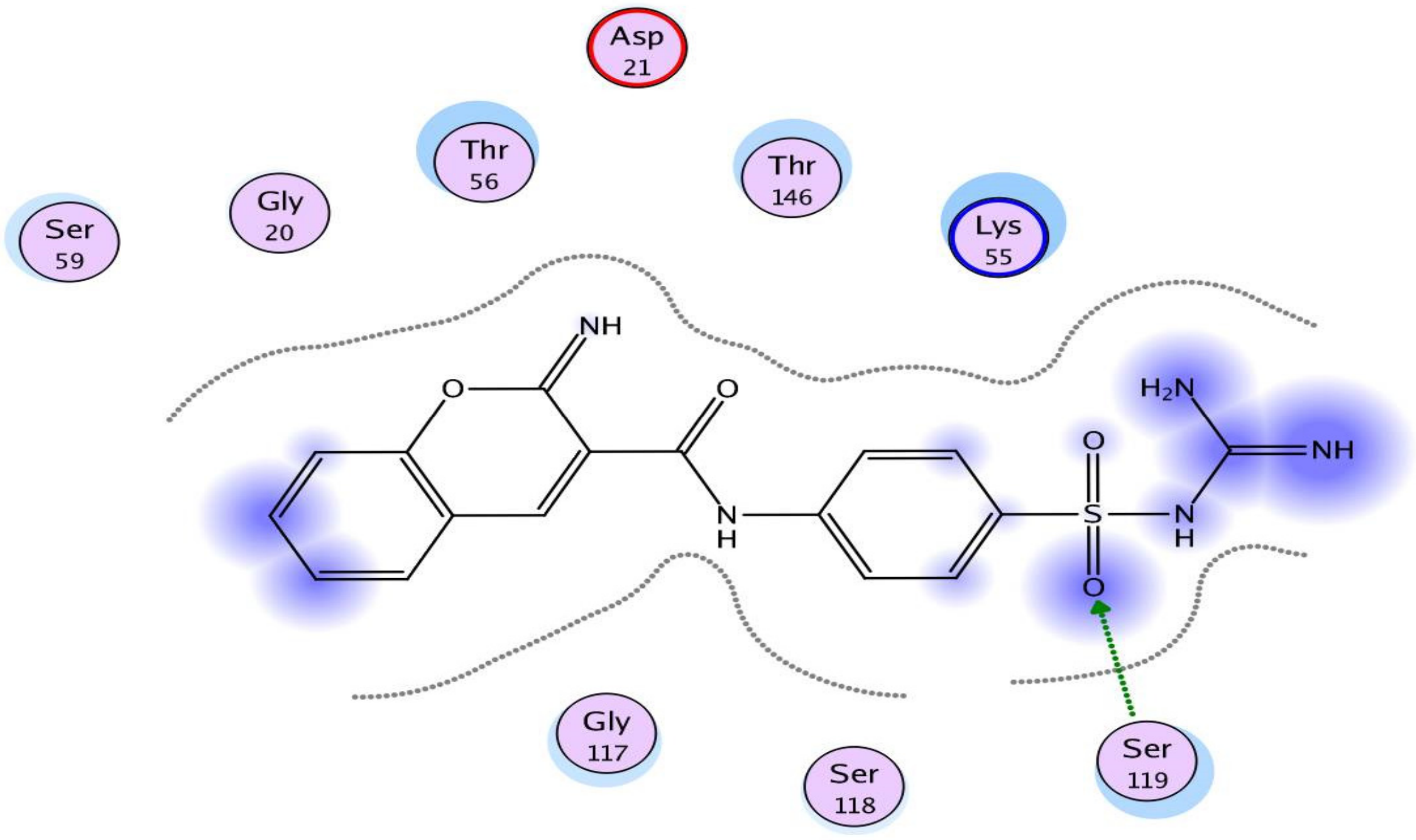


**2D structure of compound 11 inside the active site DHGR (1DLS).**

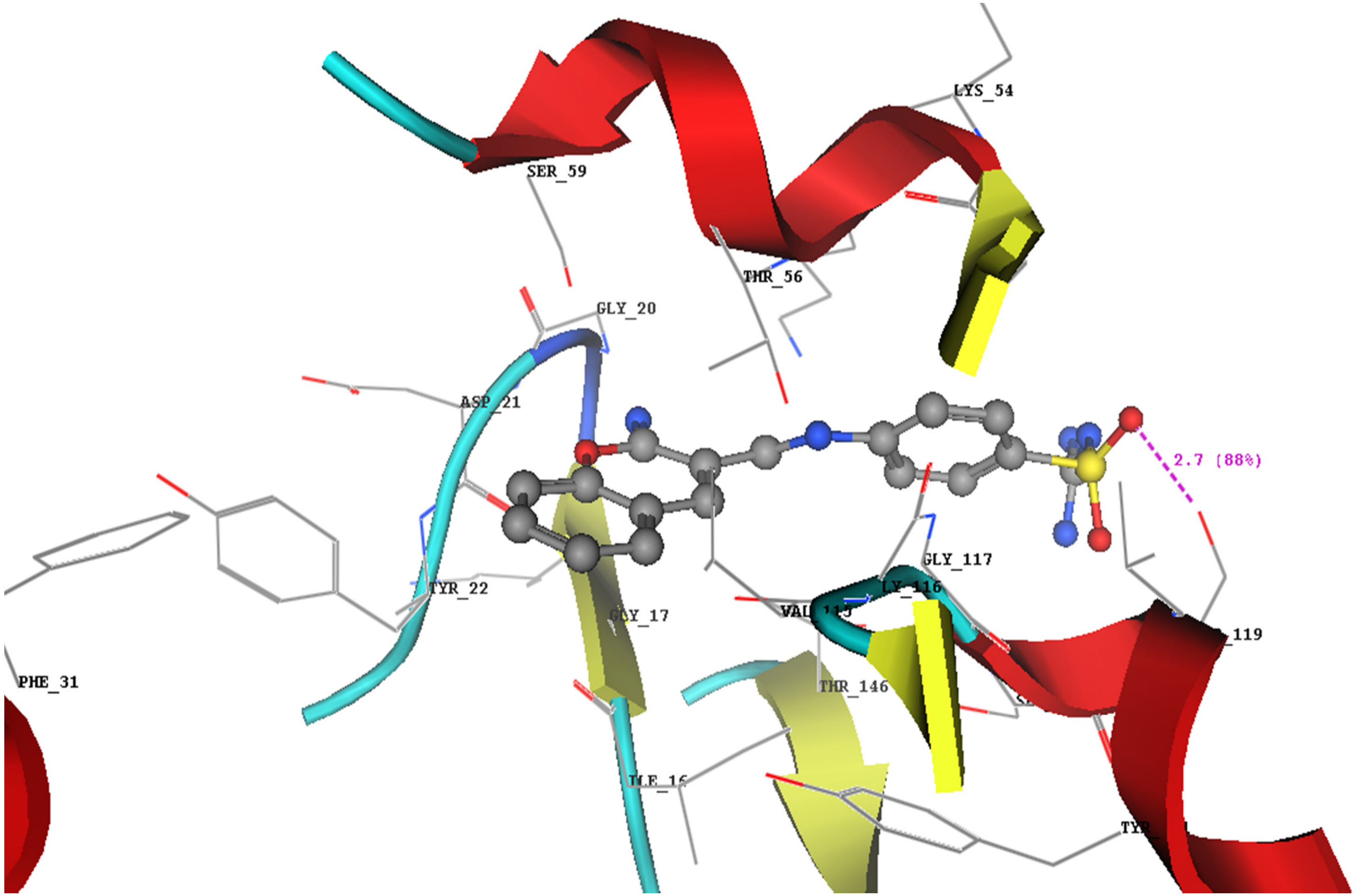


**3D structure of compound 11 inside the active site DHGR (1DLS).**
